# Anomalous Sodium Insertion in Highly Oriented Graphite: Thermodynamics, Kinetics and Evidence for Two‐Sided Intercalation

**DOI:** 10.1002/anie.8139859

**Published:** 2026-03-28

**Authors:** Chuanhai Gan, Chuanlian Xiao, Hongguang Wang, Peter A. van Aken, Rotraut Merkle, Sebastian Bette, Bettina V. Lotsch, Joachim Maier

**Affiliations:** ^1^ Max Planck Institute For Solid State Research Stuttgart Germany

**Keywords:** electrochemistry, graphite, kinetics, sodium intercalation, thermodynamics

## Abstract

The difficult intercalation of sodium (Na) into graphite is studied by systematic and long‐time investigations (of up to 2 years) using highly oriented pyrolytic graphite (HOPG). In this way a comprehensive picture of the thermodynamics, kinetics and the atomistic situation is arrived at. The results do not only allow us to draw conclusions on the applicability in Na‐based batteries, but also to understand the anomalous behavior of Na within the alkali metals as regards open circuit voltage (OCV), storage capacity, storage kinetics, and atomistic storage pattern. The round picture requires including entropic effects and space charges into the established discussion. Such considerations even give new insight into the staging mechanism as such. The storage was performed both chemically and electrochemically over a wide temperature range. Our analyses show that at room temperature higher Na concentrations may be thermodynamically possible, but they are kinetically out of reach. The sodiated samples were investigated by electrochemical tools, by chemical analysis as well as by advanced electron microscopy. The latter reveals an unexpected, striking storage pattern: Unlike the other alkali metals, Na enters HOPG predominantly in the form of bilayers before it forms larger aggregates and finally staging compounds.

## Graphical abstract

Sketch for chemical Na bilayer (a) aggregation to higher aggregates (b), resulting in loss of space charge zones. The electrons compensating for the Na^+^ are partly in the space charge layers and partly in‐between the Na‐layers.

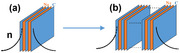

## 1 Introduction

Lithium (Li)‐based batteries have quickly penetrated our daily life owing to their large storage capacities combined with sufficient power densities. These features rely on the availability of high‐performance electrode materials. Currently, the material of choice for the negative electrode is graphite. It is inexpensive and allows for rapid and extensive intercalation and deintercalation of Li. The significance of Li‐based batteries ranges from powering commodities to enabling electromobility or efficient grid storage. This ubiquitous demand is in conflict with the limited resources [1]. From that point of view, Na‐based batteries are advantageous, and their electrochemical properties have long been underestimated [2]. Various positive electrode materials are available that quickly store significant amounts of Na [2]. Nonetheless, the negative electrode remains a severe problem and the low Na storability in graphite still an unsolved topic.

The observation of Na insertion into carbon materials dates back to as early as 1925 [3]. Na‐intercalated graphite compounds were firstly reported in 1958 [4], while potassium (K)‐ and Li‐ intercalated graphite compounds had been described earlier, in 1926 [5] and 1955 [6], respectively. Owing to the interest in Na‐based batteries, there have more recently been numerous investigations of Na insertion in graphite [7, 8, 9, 10, 11, 12, 13, 14, 15]. All authors agree on observing very low if not immeasurably small insertion at room temperature. Higher solubilities have only been reported for high pressure and high temperature (HT) chemical insertion [16]. Relatively high Na concentrations have been reported when the insertion goes along with solvent co‐intercalation (in particular for glymes) [14, 15, 17, 18, 19]. The larger solubility in “hard carbon”, the canonical negative electrode material for Na‐based batteries, appears to be largely due to stabilizing Na at higher‐dimensional defects, going along with an OCV vs. Na that is close to zero and does not stabilize Na enough to avoid the danger of dendrite formation [20, 21, 22]. The reason why Na intercalation in graphite is not favored has been inspected by various authors [16, 23, 24, 25, 26, 27, 28]. In spite of the multitude of investigations, systematic long‐time experiments have not been reported, so that the saturation concentrations are unclear. Even the decision between thermodynamic control and kinetic control could not be made, let alone a decision on diffusion or interfacial control. Beyond that, a microscopic picture of Na intercalates has not been given.

The present work distinguishes itself from works presented in the literature in essentially three respects: (i) It presents a systematic and comprehensive long‐time study (up to 2 years) of chemical and electrochemical insertion into pure and coated HOPG at various temperatures, allowing us to discriminate between thermodynamic control, diffusion control, and reaction control, and moreover to measure or derive equilibrium intercalation potentials. (ii) The combined use of advanced transmission electron microscopy (TEM), x‐ray diffraction (XRD) and chemical analysis yields the surprising but clear information that the intercalation pattern is dominated by two‐sided intercalation at HT rather than monolayers, and also by higher aggregates at low temperature (LT). (iii) Our thermodynamic considerations explicitly include entropy effects which proved to be key to understand the intercalation pattern. The explicit taking account of space charges and space charge repulsion allows for new insight into and for providing a round picture of the staging mechanism of alkali metal intercalation.

**FIGURE 1:**
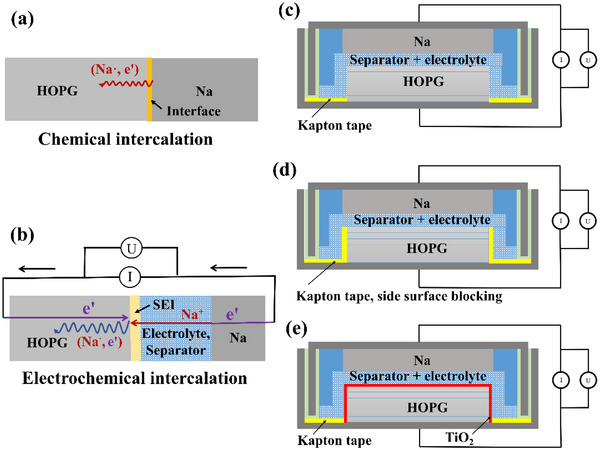
Sketches of the cells for Na intercalation into HOPG via chemical method (a) and electrochemical method (b), schematic set‐ups for CV measurements of pure HOPG (c), of side surface blocked HOPG (d) and of TiO_2_ coated HOPG (e).

Since in the literature neither the graphite samples used have been well characterized nor waiting times have been sufficiently long, (i) literature results are contradictory, (ii) Na concentrations reported are not based on reliable analyses, (iii) and to reiterate, no conclusions on thermodynamic or kinetic control could be drawn, (iv) let alone on the structural insertion features. Here, we report on systematic chemical and electrochemical (Figure 1a,b) insertion into HOPG on time scales of hours, months, and years accompanied by careful reference experiments (Figure SIII‐1) in order to arrive at a comprehensive picture.

## 2 Results and Discussion

### 2.1 Experimental Details for Chemical and Electrochemical Insertion

**FIGURE 2:**
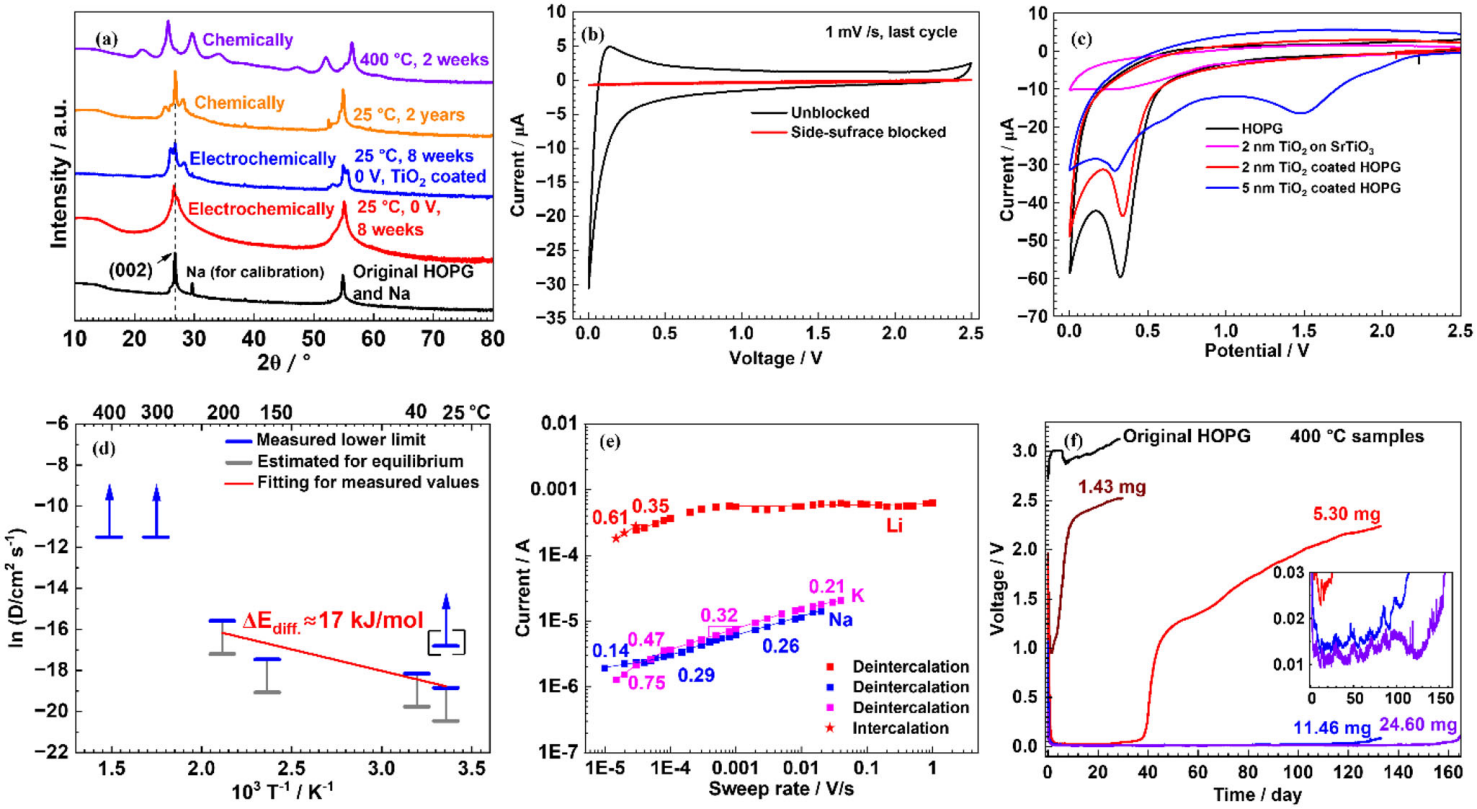
(a) XRD patterns for chemical and electrochemical intercalation of Na into HOPG (TiO_2_ coated‐5 nm TiO_2_ coating on HOPG). CV measurements for Na (de)intercalation into / from (b) pure and side‐surface blocked and (c) TiO_2_ coated HOPG at 25°C; Nb doped SrTiO_3_ was used as a zero‐storage comparison. (d) Apparent chemical diffusivities as a function of temperature. Solid‐arrow data points indicate lower limits under thermodynamic control or interfacial control. The other data refer to HOPG for which sloped profiles are observed and lower and upper limits could be estimated (profiles, see Figure SIII‐6d, and Table SII‐1). Apart from the 25°C and 40°C experiments (which are from electrochemical intercalation into 2 and 5 nm TiO_2_ coated HOPG, respectively), the data stem from chemical intercalation of uncoated samples. The black bracketed 25°C value (electrochemical intercalation in uncoated HOPG) suffers from co‐intercalation which seems to accelerate the process. The apparent activation energy for the roughly linear LT part is ∼17 kJ/mol (red fitting line). (e) Current vs potential sweep rate at 25°C. Note that the LT experiments are interfacially controlled; this explains why the areas of the cycles for Na and K are more similar than deduced from the equilibrium situation (Figure SIII‐15). (f) OCV for Na intercalated HOPG at 25°C (sample masses are indicated; inset is zooming into the plateau range; for more details, see Figure SIII‐18).

The chemical insertion was done by direct contact to solid or liquid (or even gaseous) Na and characterized by XRD and chemical analysis. XRD investigation gives qualitative information on whether or not perceptible amounts of Na have entered HOPG (appearance of new peaks at lower and higher diffraction angles compared to original (002) peaks of HOPG as depicted in Figure 2a) or polycrystalline graphite powder (Figure SIII‐2, also see detailed XRD data analysis in Part SIV). Chemical analyses by titration [29] and inductively coupled plasma optical emission spectroscopy (ICP‐OES) deliver quantitative information on the average composition, provided the molar Na fraction is higher than 5×10^−5^  (NaC_20000_). For chemical insertion, a wide temperature range from 25°C to 500°C was covered (Figure SIII‐3a), and waiting times of up to 2 years were allowed for (Figure SIII‐3). For 25°C, 90°C, and 110°C (below and above the Na melting point of 98°C), also coatings with TiO_2_ or amorphous carbon were employed.

The electrochemical insertion experiments were performed (Figure 1b) using the cell Na / liquid electrolyte (1 M sodium hexafluorophosphate (NaPF_6_) (or sodium bis(fluorosulfonyl)imide (NaFSI))‐EC/DMC) / HOPG, whereby pure and coated HOPG (for TiO_2_ coating by atomic layer deposition as well as amorphous carbon coating [30, 31], see Materials Synthesis, Supporting Information) were used. Here, the temperature was around 25°C and the waiting times ranged from 1 week to 8 months (Figures SIII‐3d–4). While coating in the chemical experiments was rather done for the purpose of comparison, it was necessary in the electrochemical experiments in order to block co‐intercalation. As regards usefulness of TiO_2_, or better Na_x_TiO_2_, as a stationary Na‐permeator see the Part SII‐2.

Unsurprisingly, we found in targeted experiments (Figures 1c,d) that insertion only occurs perpendicular to the *c*‐axis (parallel to the carbon layers), so that the edge regions (side‐surface) of our samples must be available for insertion as shown in Figures 2c and SIII‐5. The results justify the neglect of higher‐dimensional defects such as dislocations as far as perpendicular long‐range transport is concerned. Insertion of Na perpendicular to the *c*‐axis should, kinetically speaking, represent the upper limit of insertion into HOPG samples.

### 2.2 Chemical and Electrochemical Insertion in HOPG: Kinetic Picture

#### 2.2.1 Chemical Insertion

Let us first discuss the results of chemical insertion. At 400°C and 300°C waiting times of 2 days and 2 weeks, respectively, are enough to reach saturation (for Na activity *a*_Na_ =  1 with compositions on the order of NaC_100_ (Table SII‐1, Figure 4a, Figure SIII‐6). (Polycrystalline graphite powder behaves similarly according to XRD measurement, Figure SIII‐2). In all these cases Na is present in liquid form. Na vapor intercalation into HOPG at 400°C for 2 days yields significantly lower values, which is expected due to the lower Na activity (low Na vapor pressure, Figure SIII‐7). Figure 2a and Table SII‐1 summarize the insertion results.

Insertion experiments at LT (< 100°C) suffer from the typical problems of solid–solid contacts. Samples synthesized at 25°C show distinctly lower Na contents (∼NaC_13000_) for a waiting time of 2 weeks, and not very different values (∼NaC_14000_) for a waiting time of 1 year (whilst Li and K can intercalate easily, see Figure SIII‐3e,f). Comparison with the electrochemical insertion (see below) indicates that it is still very far from saturation and that even 2 year‐long waiting times are too short to reach saturation (also indicated by XRD data, Figure SIII‐3c,d). Chemical insertion at LT does not perceptibly benefit from coating (∼NaC_13700_, Table SII‐1, Figure SIV‐3c) by TiO_2_ or by amorphous carbon.

At 400°C, coating by thin layers of TiO_2_ and amorphous carbon show similar results to uncoated HOPG (whereas a thicker coating, e.g., 10 nm TiO_2,_ slows down Na intercalation (Figure SIII‐8a). Whilst this is expected as at these temperatures interfacial kinetics is comparatively fast, the negligible influence of coating at LT should be ascribed to the difficult solid–solid contact (Na/solid storage medium). These coating results give us the confidence to use such coatings as Na filters in the electrochemical experiments to be discussed now.

Worthwhile information on the incorporation kinetics comes from cutting the samples and comparing inner (far from the Na source) and outer parts (close to the Na source, see Figure SI‐1(d) using XRD or titration measurements. For the HT (400°C and 300°C) samples, after a waiting time of 2 days, no difference was observed which is in line with the fact that 2 days were sufficient to reach equilibrium (Figure SIII‐6a,b). For 200°C, where a waiting time of 2 days was shorter than the equilibration time (Figure SIII‐6c), a significantly higher Na content in the outer part was found when compared to the inner part indicating diffusion control. At 25°C, from 2 weeks to 2 years, again no perceptible difference could be observed (Figure SIII‐3c,d), indicating that now interfacial control has taken over. This transition from interfacial control to diffusion control and finally thermodynamic control on increasing temperature is not unexpected in view of the typical activation energies for interfacial and diffusion kinetics. In agreement with this, coating does not change the HT values, while at LT it can have a significant impact on the kinetics (even at 150°C, Table SII‐1). The comparison between electrochemical intercalation for coated and uncoated samples is very revealing in this context (Figure SIII‐3d). While the results for the uncoated samples are the same as for the chemical intercalation and are definitely interfacially controlled (horizontal profile), the values for electrochemically intercalated coated samples show a diffusion profile indicating that the coating could substantially reduce the interfacial resistance.

#### 2.2.2 Electrochemical Insertion

As far as electrochemical insertion is concerned, we meet different interfacial problems. The difficult solid–solid contact is absent, but the Na‐liquid electrolyte contact is chemically unstable resulting in SEI formation, characterized by XPS (Figure SIII‐9), EDX (Figures SIII‐10, ‐11, ‐12) and CV (Figure 2c). Accordingly, we face—for kinetic reasons—higher storage (at 25°C) than for LT chemical insertion, but still distinctly smaller storage than observed at HT. For uncoated HOPG, the highest Na content was ∼NaC_300_ for electrochemical insertion (for a waiting time of 8 weeks, Table SII‐1). Yet, these values are not reliable as evidenced by the observation of severe sample expansion that we attribute to co‐intercalation most probably of the solvent (Figure SIII‐13 shows exfoliation for the uncoated HOPG while no expansion for the TiO_2_ coated HOPG). For these reasons we rely on TiO_2_‐coated samples for the electrochemical intercalation, yielding ∼NaC_251_ (for a waiting time of 4 months), and higher Na content may be realizable at much longer time. At 40°C, the electrochemically intercalated TiO_2_‐coated samples (4 months, Table SII‐1) show a similar storage as observed at HT. Coating has various advantages: (i) The parasitic SEI formation is less marked [32] potentially facilitating the proper interfacial kinetics (Figure 2c), and (ii) very importantly, solvent co‐intercalation is suppressed (Part SII‐2, and Figure SIII‐13e,f). Impedance spectroscopy indeed shows a more stable behavior for the coated sample (Figure SIII‐14). No further conclusions about the coating's impact on kinetics could be drawn.

#### 2.2.3 CV, OCV, and Overpotential Experiments

Cyclic voltammetry (CV) using voltage ramps of various rates performed on electrochemically inserted HOPG confirms the picture. For Na, CV clearly shows that insertion requires diffusion parallel to the carbon layers (Figure 2b). The slope of logarithms of peak current versus sweep rate is less than 0.5 (Figure 2e) indicating surface control for the Na insertion into uncoated samples (for more details, see Part SII‐3). Li and K show under the same conditions diffusion control which changes to surface control only at high voltage‐sweep rate; notably the kinetics worsen drastically from Li to Na and then improve only slightly toward K. This explains the rather similar areas of Na and K in the CV experiments (Figures SIII‐15a–c and 16), (similar diffusion coefficients and similar interfacial rate constants). Indeed, for graphite powder with its smaller particle size, the available capacity of K insertion is greater than Na, and comparable to Li (Figure SIII‐15d). The CV measurements indicate positive de‐intercalation potentials (cf. intercalation peak potentials close to 0 V) for Na being as small as 0.05 V compared to 0.45 V for K, 0.36 V for Li (Figure SIII‐16) as more accurately measured by cell potential discussed below. CV results on TiO_2_ coated material confirm lower interfacial resistance and hence faster rates which eventually would be limited by diffusion (Figures 2e and SIII‐17).

More detailed information stems from targeted OCV measurements on (Na*_y_* C)_sat._/electrolyte/Na (sample (Na*_y_* C)_sat._ synthesized at 400°C). The transient in Figure 2f shows approximately exponential decay toward an end value which we attribute to the reversible cell voltage vs. Na (OCV) for this special composition. These end values are small indeed but positive (10–15 mV), much smaller than for Li (≥ 0.20 V) or K (≥ 0.40 V) (see Figures 2f, SIII‐18, and 19). The OCV values are consistently in between the intercalation and de‐intercalation peak potentials of CV (Figure SIII‐16). Such OCV values are not influenced by electrolyte characteristics, such as solvation free energies, as those effects cancel in the cell reaction. Thus, we can conclude that—in agreement with literature—the free energy of accommodating the alkali metal into HOPG (which should not significantly differ for usual graphite), shows a minimum for Na, when the atomic number of the alkaline metals is varied. A similar sequence is seen for the incorporation kinetics: Li can be easily intercalated at 25°C electrochemically; this also holds for K but at significantly smaller rates (CV, Figures SIII‐16, SIII‐20). Na intercalation had to be done at higher temperatures to obtain a significant alkaline amount inside. In the case of 25°C storage, we can check the distance to equilibrium by following the decrease of OCV toward zero.

The OCV values in Figure 2f show after a certain period (which decreases with decreasing sample mass) significant rises reflecting Na‐loss by SEI formation. We see a qualitatively similar behavior with Li and K but connected with larger OCV values (Figure SIII‐19c). In all cases the voltage increase occurs the later the higher the sample mass. This we can interpret as sluggish alkali metal removal from the HOPG by reaction with the electrolyte. The fact that the time until the rise starts depends roughly linearly rather than quadratically on the sample size, is consistent with interfacial kinetic control [33]. The mechanisms of interfacial control can be manifold and range from hindered phase transfer to phase formation (e.g., SEI in the electrochemical experiments) [34, 35] or may be simply governed by point contact problems (solid–solid contacts in the case of chemical experiments). The just mentioned onset of corrosion sets a time limit to our storage experiments.

The small OCV has also an important consequence on the kinetics. As we are thermodynamically close to equilibrium, the flux can be viewed as being proportional to the chemical potential gradient of Na whereby the chemical potential directly reflects the OCV. Thus, a sluggish kinetics is indeed expected. In fact, the striking qualitative parallelism between OCV (which for Na is at least one order of magnitude smaller than for the other alkali metals) and incorporation rate when the alkali metal is varied, may thus find a simple explanation. For Na OCV < RT/F, linearization is possible so that the rate is proportional to OCV. Certainly, non‐linear effects for the other alkali metals as well as variations in the kinetic parameters play additional roles.

Realizing the importance of the cell voltage for the kinetics, we deliberately apply overvoltages. If anodic overvoltages (in fact negative voltages) are imposed on polycrystalline graphite powder, we expect thermodynamically a stronger sodiation such as plating and kinetically a higher driving force for the sodiation (and after release of the bias a stronger driving force for desodiation toward the equilibrium OCV). XRD allows us to separate Na plating and Na intercalation. In fact, we observe already for voltages of −0.01 V, and even more so for −0.40 V, not only plating but also an acceleration of the kinetics. For −0.50 up to −2.00 V no further increase of the values beyond Na values being on the order of the values obtained for chemical intercalation at 400°C, was observed (Figure SIII‐21, XRD patterns are similar to equilibrium situation at 400°C). Such value fits well the thermodynamic considerations referred to below and in Figure 4a.

#### 2.2.4 Effective Chemical Diffusivities of Na in HOPG

Let us now consider the chemical diffusion coefficients (Figure 2d). For the samples that do not show concentration profiles (Figure SIII‐6a,b), we can only give lower limits. The reason for giving lower limits is that at higher temperatures the waiting times become too quick to be analyzed given the heating/cooling procedure, while at lower temperatures interfacial control has taken over. For 400°C and 300°C samples no concentration profiles remain after 1 h, meaning that $D_{\mathrm{Na}}^{\delta }$ would be higher than 10^−5^ cm^2^/s. These values are rather high and may not be exceedingly far away from Li and K diffusivities [36, 37]. In the cases where profiles could be observed, we may roughly assess upper limits. Such estimate is seconded by the fact that these values yield a reasonable activation energy (∼17 kJ/mol, similar to values for Li and K cases) [24]. At room temperature we thus may estimate a diffusion coefficient of 10^−9^ cm^2^/s. This refers to electrochemical intercalation into coated HOPG. The higher value obtained for uncoated HOPG (see Figure 2d) can be ascribed to co‐intercalation, as mentioned earlier. We must however be aware of the fact that the diffusion scenario itself is complex and cannot be identified with a simple chemical diffusion process. In particular the diffusivity along the layers is highly non‐linear. In the next section we can give a clearer account of this issue. Nonetheless we might conclude that for an insertion into HOPG to be battery‐relevant, nanocrystalline samples are necessary for which then the necessary depression of the interfacial resistance becomes very severe.

The section after the next one primarily deals with the equilibrium values (summarized in Table SII‐1). The thermodynamic conclusions given there are not in contradiction with first principles calculations in the literature that report energetic instability of NaC_6_ or NaC_8_ [26, 27]. The reasons are the neglect of entropic effects there as well as the fact that we here deal with low Na concentration values and an unexpected insertion pattern revealed by TEM which is going to be described in the following.

### 2.3 Microscopic Picture

#### 2.3.1 Electron Microscopy

**FIGURE 3:**
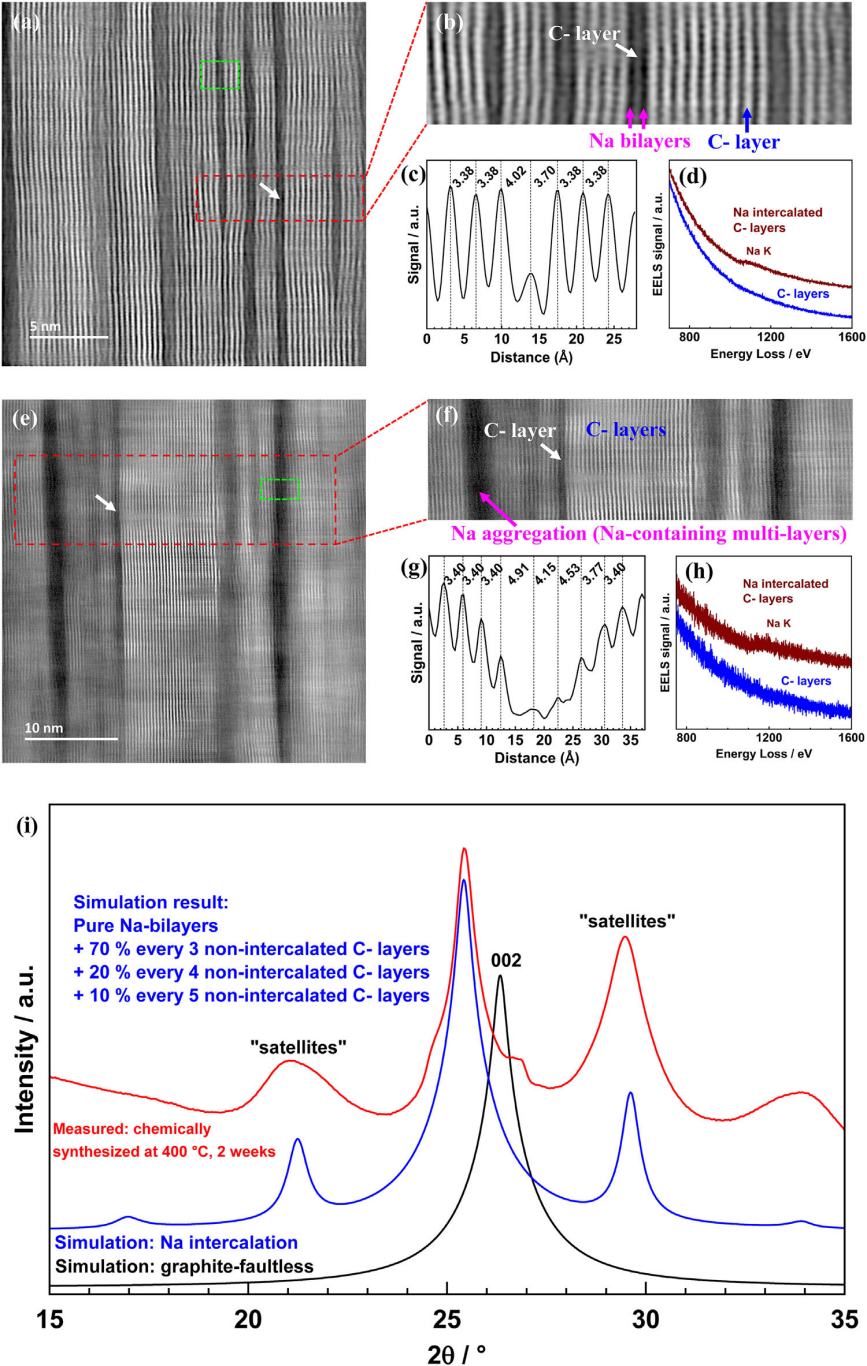
Na intercalated HOPG chemically synthesized at 400°C for 2 weeks: (a and b) HAADF‐STEM images, (c) interlayer distances for the green dashed square region and (d) Na‐K edge EELS spectra for the red arrow marked region in (a) and for the non‐intercalated carbon layers. Na intercalated HOPG chemically synthesized at 25°C for 10 months: (e and f) HAADF‐STEM images, (g) interlayer distances for the green dashed square and (h) Na‐K EELS spectra for the red arrow marked in (e) and for the non‐intercalated carbon layers. The TEM images combined with the interlayer distances and the EELS data can only be explained by dominance of Na bilayers. (i) Na intercalated HOPG chemically synthesized at 400°C for 2 weeks: Comparison of the measured with the simulated patterns.

For HT‐equilibrated samples (cooled to 25°C), TEM results show, contrary to expectations, predominant two‐sided intercalation (Figure 3a–d), similar to alkali metal adsorption on both sides of a single graphene layer [38, 39]. These bilayers sandwiching single carbon layers are separated by 4–10 pure carbon‐layers. (In order to not confuse these “chemical Na bilayers” with electrostatic double layers, or with layers discussed for superdense sodiated graphite obtained by high pressure synthesis where two Na layers are directly neighbored [16], we use the term “bilayer”) The spacing between the pure carbon layers is ∼3.40 Å (Figure SIII‐22), not perceptibly varying with distance from the Na‐layer while the carbon–carbon layer spacing in the Na intercalated bilayers is 3.60–4.02 Å. Some bilayers show even varying spacings within the same bilayer (Figure 3c) which we explain by varying under‐occupancies (next section). In contrast to other alkali metals, we never observed monolayers in the TEM images. At HT we observed prevalent Na bilayers (more than 70%, see statistical TEM images, Figure SIII‐23), only rare occurrences of trilayers. While at LT, the vast majority of bilayers have disappeared and higher aggregates dominate (Figure 3e–h, for additional TEM images on 25°C chemical and electrochemical samples, see Figures SIII‐24 and 25). We make sure by using different cooling rates that no time effects falsify our results and thus the quenching is sufficiently quick to avoid aggregation during quenching (Figure SIII‐8b). We are also certain that we can neglect Na loss stemming from the impact of the electron beam. The necessary support is given in the SI (Appendix, Consecutive STEM imaging).

#### 2.3.2 XRD Simulations and Analyses

XRD analyses and simulations (Part SIV) were performed for samples chemically synthesized at HT, 25°C, as well as electrochemical intercalation samples. We performed XRD simulations of the 400°C chemically intercalated sample for two different Na intercalated interlayer distances. (Note that the Na intercalated interlayer distances and the number of empty carbon layers are variable, as mentioned above.) The first is for a representative mean value of 3.80 Å. The result (Figure 3i) is identical to the TEM finding that only bilayers and no monolayers are present. To check the reliability range, we also performed a simulation with the extreme distance value of 4.02 Å. Then monolayers could coexist but would still be in minority compared to bilayers (Figure SIV‐2). For the chemically intercalated LT‐samples (25°C) a rather mixed structure with various kinds of intercalated layers is confirmed, even admixture with non‐intercalated phases is possible (00l’ peak, Figure SIV‐3b); for electrochemical intercalation at 25°C the results are similar, but only if coating is used (Figure SIV‐3c,d). Otherwise, solvent co‐intercalation occurs significantly changing the diffraction pattern.

#### 2.3.3 Distribution of Na Layers in HOPG

Comparison of the microstructure with chemical analysis (Table SII‐1) suggests an under‐occupation of the Na bilayers. This would also explain the distribution of the unequal carbon–carbon distances of the Na bilayers (Figure 3c). (Should there be remainders of adsorbed Na left or Na stored at higher dimensional defects, then the calculated occupation would be even less.) It is relevant to mention that for the other alkali metals the reduction of staging degree up to the AC_6_ or AC_8_ (A = alkali metals) composition is reported to be accompanied with increasing occupation within the layers [29, 40, 41].

As entropy contributions caused by under‐occupation of that magnitude are small (Part SV) the following hypothetical picture suggests itself: Instead of occupying a layer fully (“fully” refers to the maximum occupancy of the interlayer space in a AC_6_ or AC_8_ structure) these Na ions are dispersed over the two layers more or less orderly so that the Na ions are not directly above each other. The energetic benefit of this configuration is explained below. The expected objection that there is no atomic mobility through the carbon layers, is not relevant, as the distribution can be established by in‐diffusion along the layers. The kinetics may be substantially facilitated by domain formation as discussed in Refs. [42, 43] (cf. also Figure 3a). (Higher‐dimensional defects could also provide leakage in the perpendicular direction.) The perceptible electronic mobility should be sufficient to allow for extended delocalization also out‐of‐plane [44].

A clearer picture of the in‐diffusion kinetics is provided by TEM at an early stage at 25°C (Figure 3e–h). For samples chemically sodiated at 25°C, we are definitely referring to the regime controlled by (interfacial) kinetics. Accordingly, the Na content is small. TEM still reveals—apart from dominant two‐sided intercalation (bilayers)—to a significant degree also trilayers, quadruple layers and some even more highly aggregated layers, separated by pure carbon‐layers in between. The separation by pure carbon‐layers is far beyond space charge repulsion (range of elastic fields is controversially discussed but it is less than 10 carbon layers in non‐equilibrium, and very narrow in the ordered state [45, 46]) and indeed no ordering can be detected. Owing to our reasoning, ordering should start when the relevant interaction length scales are reached. How much repulsion—and hence which proximity—can be afforded, is a matter of Na activity (“chemical pressure”). The fact that after an initially expected random distribution, we see homogeneous bilayers right in the beginning and no diffusion profile (Figure SIII‐3d) within the intercalation layers, means that the diffusivity is highly non‐linear, more precisely self‐amplified. It seems that a critical presence of the alkali element—probably because of the local expansion—accelerates diffusivity and thus favors further incorporation in these layers explaining the meaningfulness of the staging concept. (A similar acceleration is known for FeCl_3_ intercalation) [47, 48]. At a first glance it may be difficult to figure that the neighboring layer is favored also in the transient state in view of local expansion. But note that we in reality deal with a two‐dimensional situation where this problem does not critically arise and the above energetic preference predominates. For very fast diffusion within the layer, only filled or partially butstatistically filled layers ought to be observed; the progress of insertion will then consist in realizing more of the occupied layers being initially isolated and eventually aggregated. The fact that we observe a homogeneous bulk situation (in the direction of Na flux) is a consequence of the interfacial reaction control. On a large scale and at times on the order of the diffusion time constant, one should of course also observe non‐percolating layers. (In this context, it must also be borne in mind that domain formation can be helpful in fastening the kinetics [42, 43].) As will be shown in the next section, aggregation is energetically favored at LT, and the small aggregates hence must be seen as kinetic intermediates toward more massive aggregates. In the diffusion‐controlled regime, “growth” of such layers should be observable (parallel to the increase of bilayer density) giving rise to the concentration profiles already described above.

### 2.4 Thermodynamic Picture

**FIGURE 4:**
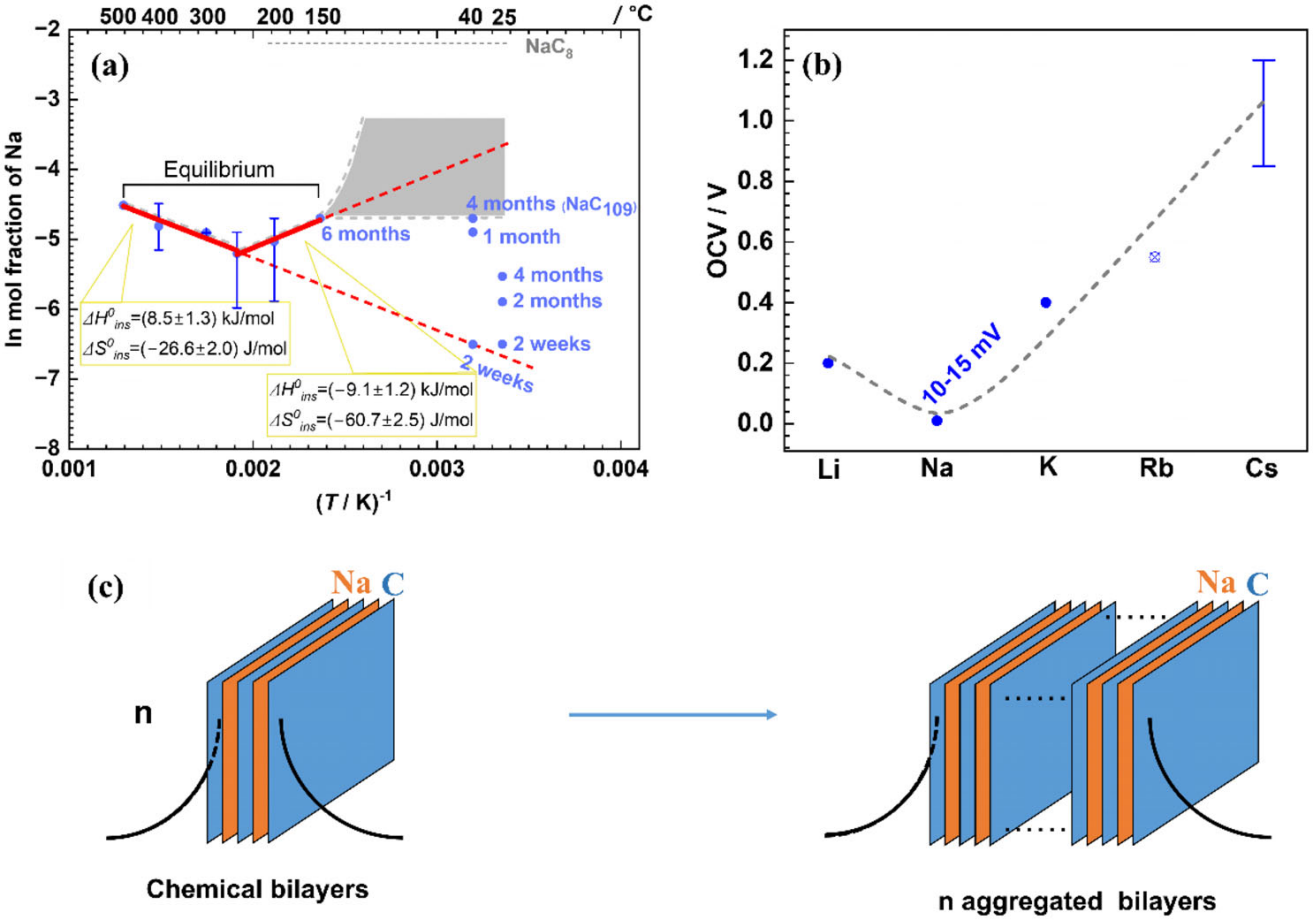
(a) Na concentration versus temperature plot (gray area indicates the range of equilibrium values that is expected from our thermodynamical analysis; bold‐red solid line indicates regime of thermodynamic control). Data at 25°C and 40°C were obtained from electrochemically intercalated samples (TiO_2_ coated). The bars include not only the error of analysis, but also the distribution within a given batch. For 2‐weeks and 2‐months intercalation experiments (25°C), 2 nm TiO_2_ coated HOPGs were used, and 5 nm coated samples were utilized for the longer time (longer than 2 months) or 40°C experiments. Data at other temperatures (above 40°C) were acquired from chemically intercalated samples. For detailed concentrations, see Table SII‐1. (b) OCV at 25°C (OCV for NaC_∼100_ synthesized at 400°C, and AC_40‐100_ for the other alkali metal intercalated graphite compounds. The Rb value is from room temperature electrochemical data of ref. [49]. while the Cs value is estimated from 600°C chemical data of ref. [53]. Dashed curve is a guide to eye.). (c) Sketch for chemical bilayer aggregation to higher aggregates, resulting in loss of space charge zones. The electrons compensating for the Na^+^ are partly in the space charge layers and partly in‐between the Na‐layers.

The thermodynamic analysis shall conclude our comprehensive study. For this, we primarily concentrate on the samples for which we can assume dissolution equilibrium. In Figure 4a, the solubility values are logarithmically plotted versus 1/*T*. At HT the graph shows roughly a van't Hoff behavior while on lowering the temperature it increases again. Comparing the Na values with our values for Li and K we find much lower equilibrium content for Na than for the other alkali metals (∼NaC_100_). The OCV values follow the sequence (Na) < (Li) < (K) < (Rb) < (Cs) (Figure 4b). This is in accordance with literature [26, 27, 49] where similarly low or even significantly lower if not zero values have been reported for Na. The OCV values (corresponding to the negative free enthalpies of reaction) are slightly above zero (This is consistent with Ref. [12]. claiming 2 mV at 300°C.), in other words a high activity is required for obtaining this rather modest Na content.

When evaluating the 1/*T*‐dependence of the logarithms of the HT equilibrium contents, we derive a small positive enthalpy of intercalation (unlike Li [50], K [51], Rb [52], Cs [53]) and a negative entropy value (like Li, K, Rb, Cs): $\Delta H_{\mathrm{ins}}^{0}=\left(8.5\pm 1.3\right)\;\mathrm{kJ}/\mathrm{mol}$; $\Delta S_{\mathrm{ins}}^{0}=\left(-26.6\pm 2.0\right)\;J/\mathrm{mol}\;K$, respectively. Given these small Δ*H*‐values, this is not in contradiction to the small but positive OCV data for the quenched HT samples as the OCV contains also the configurational contribution and relates to Na incorporation into Na‐containing carbon‐layers.

One should also not forget that the van't Hoff relation presupposes a random distribution. Thus, in view of the complex intercalation pattern these values are only effective values and are discussed again below. Moreover, if the Na^+^ and e^−^ were completely independent, the measured slope would be half of the incorporation enthalpy (Part SV‐5); but since we do not treat charge carriers separately, this is only a question of notation. For the HT samples TEM and XRD reveal overwhelmingly two‐sided intercalation whereby these intercalated regions are under‐occupied bilayers separated by typically 4–10 pure carbon‐layers. At LT, however, higher aggregates are observed even if equilibrium has not yet been reached.

There are several points to be explained: (1) What is the reason for the storage capacity minimum in the free energy for Na along the series of alkali metals? (2) Why do we observe two‐sided intercalation and under‐occupation? (3) Why are these layers typically separated by about 4–10 pure carbon‐layers? (4) Why do we observe higher aggregates in the LT samples? Let us address these points step by step.

#### 2.4.1 Apparent Storage Anomaly of Na

Because electrolyte and thus solvation influences disappear in the cell reaction $A+xC\to AC_{x}$ (with A being short for the alkali metal element) and because entropic differences for the formation free energy can be assumed to be rather small (see Part SV‐2), the answer to the question why Na shows a lower capacity than the other alkali metals, must lie in the accommodation energetics. We have observed a positive intercalation enthalpy at HT (Figure 4a), lowering its value with the clear tendency to change sign when the temperature is decreased. Unfortunately, the stability of the coating limits the time window of electrochemical intercalation. At any rate, the measured absolute values are small in contrast to the distinctly negative values in Li and K cases. Nobuhara et al. [26]. and Liu et al. [27]. could reproduce the OCV sequence (Na) < (Li) < (K) < (Rb) < (Cs) as far as the energy is concerned using first principles calculations for AC_6_ and AC_8_. Liu et al. decomposed the insertion process into atomization of A, straining (i.e., stretching and expanding) graphite to the state that is finally achieved, ionization of A plus transferring the electron to the carbon‐substrate, and then coupling of the cation to the negatively charged graphite (involving electrostatic and quantum‐chemical bonding effects). They found all individual contributions to be monotonic from Li to Cs. Coupling and straining were calculated to be costly when compared to Li (monotonically increasing from Li to Cs), while ionization and decohesion are beneficial when compared to Li (monotonically decreasing from Li to Cs). The overall process then shows a maximum in energy for Na insertion in agreement with the experiments. As the coupling dominates the costly contributions and the ionization the beneficial ones, Liu et al. could conclude that it is essentially their counteraction which leads to the non‐monotonicity. (We interpret the distinctly lower coupling energy for the Li‐ion to the negativated carbon‐matrix simply as expression of the higher covalency of the Li─C bond.) So, from the standpoint of covalency, one could also consider Li as an outsider in an otherwise monotonic variation from Na to Cs. Importantly, the overall formation energy is found to be positive for Na in the fully occupied AC_6_ and AC_8_ structures. This is not in contradiction to our findings that the slope in Figure 4a changes sign ($\Delta H_{\mathrm{ins}}^{0}=\left(-9.1\pm 1.2\right)\;\mathrm{kJ}/\mathrm{mol}$; $\Delta S_{\mathrm{ins}}^{0}=\left(-60.7\pm 2.5\right)\;J/\mathrm{mol}\;K$, at the temperature range of 150°C – 250°C) or that the OCV is slightly positive (Figure 4b), as (i) the inclusion of entropy could easily bring down the free energy to negative values as the absolute value of the energy is small; (ii) we deal with much smaller Na contents than considered by Liu et al. which are expected to substantially reduce the energy, and (iii) OCV and intercalation do not exactly refer to the same reaction (Part SV‐7) even though the difference may be small for very small Na contents.

#### 2.4.2 Monolayer Versus Bilayer

The following scenario still needs to be explained: Li, K, Rb, and Cs form monolayers which become progressively less separated (decreasing staging number) as activity increases, and eventually form full aggregates (stage 1) [6, 49, 54, 55]. Na however prefers under‐occupied bilayers with increasing ordering and aggregation on temperature reduction. Full aggregation is not seen even at 25°C for *a*_Na_ = 1, very probably due to the sluggish kinetics.

Let us now discuss the benefit of the two‐sided intercalation. As entropy advantages should be small (see Part SV‐2), we have to seek the explanation in the gain of enthalpy. Incomplete filling (allegedly 50% when compared to the AC_6_ or AC_8_ structure) as discussed above with the aggregation pattern from TEM as outlined in Supporting Information (Part SII, 1–2) would indeed be consistent with a favorable energetic effect as then the Na ions can gain Madelung energy, in particular if the Na ions of the two layers are displaced with regard to each other in consistency with an under‐occupation of about 50%. (Interestingly, an under‐occupation of 50% would also be entropically favored if the energetic effect is negligible, Part SV‐4.) The fact that the two spacings within bilayers can be different (Figure 3c), indicates varying under‐occupancies. Unsurprisingly, literature reports under‐occupancy for the other alkali elements too if the staging number is large [29, 40, 41]. To reiterate: Two‐sided intercalation is not inconsistent with a zero mobility of Na through the layers, as the fast in‐diffusion occurs along the layers. How the mechanism for bilayer filling looks like in detail, has to be clarified in the future. Certainly higher‐dimensional defects, if present, would significantly facilitate the kinetics by allowing for perpendicular transport.

Strikingly, for the other alkali metals, besides fully aggregated (stage 1) states only monolayers (higher stage numbers) have been reported. In these cases obviously, and unlike Na, monolayers are tolerable and aggregation to fully occupied aggregates is, whenever possible, more favorable than bilayer formation. The cause is most likely to be again sought in the counteraction of ionicity and size. Ionicity increases (covalency decreases) from Li to Cs and favors distribution over more than one layer involving under‐occupation. Note that the lower Li ionicity is not directly determined by the only slightly higher ionization potential in the gas phase (compared to Na) [56], but by the value in the carbon environment which should be substantially varied due to the covalency of the Li─C bond (cf. coupling term in refs. [27, 57]); the measured dipole moments have been reported to be markedly less than the value expected from a completely ionized bond (6 instead of 9.5 Debye) [58]. Part SV‐6 gives simple arguments that not only from a Madelung perspective (ionic perspective) but also from the perspective of delocalization (electronic perspective), the energy gain from monolayers to bilayers is greater than from bilayers to higher aggregates making a preference for the Na‐bilayers in terms of free energy very plausible. This holds if the ions are ionized and do not induce strain. Obviously, Li is too covalent (low Madelung contribution) and the higher alkali metals too big (too high strain) to favor bilayers within the carbon matrix (note that the energy connected with misfit stress depends quadratically on the misfit assuming that this effect is not compensated by the lower occupancy). Thus, Na is expected to offer the best parameter compromise as to favor bilayers. Certainly, all this offers an interesting task for ab initio calculations which might also clarify the relevance of how important the fitting of bilayer stacking and matrix stacking is for the intercalation.

#### 2.4.3 Revisiting the Staging Phenomenon

Let us now refer to the thermodynamic stability of staging compounds in general. In order for intercalated layers to be separated by non‐intercalated layers rather than aggregating, repulsion between them must predominate over attraction. (Note again that the entropic effect on rearranging the layers is negligible.) Elastic interactions are either negligible (as for Li [59], or Na) and/or of low range, so we concentrate on the electric effect. In an adapted screening model [46, 60], it was shown that screening lengths can reach 5 – 8 Å, that is, values higher than assumed by Dresselhaus et al. (“one atomic layer”) [48]. A critical distance may be assessed to be 4 times this value corresponding to about 10 layers [61], nicely agreeing with the TEM results. This is nevertheless very approximate, also in view of the fact that the interaction of the alkali metal with the carbon layers can extend up to a few carbon layers [62]. For Li the higher covalency is an additional factor (in the case of zero ionization the electric double layer effect would be zero).

So far, we have neglected electric double layers formed by the electron cloud screening the alkali ion arrays. The thermodynamics of a diffuse electric double layer interaction (do not confuse this with the Na bilayer intercalation) stemming from truncating the semi‐infinite extent (very large distance, no interaction) of the electric potential to the finite profile with the value in the middle being larger than the corresponding semi‐infinite values, has been treated by Overbeek in ref. [63]. As pointed out by Overbeek, this confinement of the charge carriers reduces the configurational entropy which in most examples exceeds the energy effect connected with the approach of the diffuse space charge zones. Surprisingly, such entropic effects have never been discussed for staging compounds though being popular in colloid chemistry. Naturally the number of the separating carbon‐layers and thus the degree of tolerated double layer repulsion is a function of the alkali metal activity (cf. voltage). In Na compounds the intercalation energy is not very favorable such that the intercalation layer separation is larger than for the other alkali metals for which a smaller if not vanishing distance (corresponding to an increasingly lower staging degree) is possible for the same condition.

**FIGURE 5:**
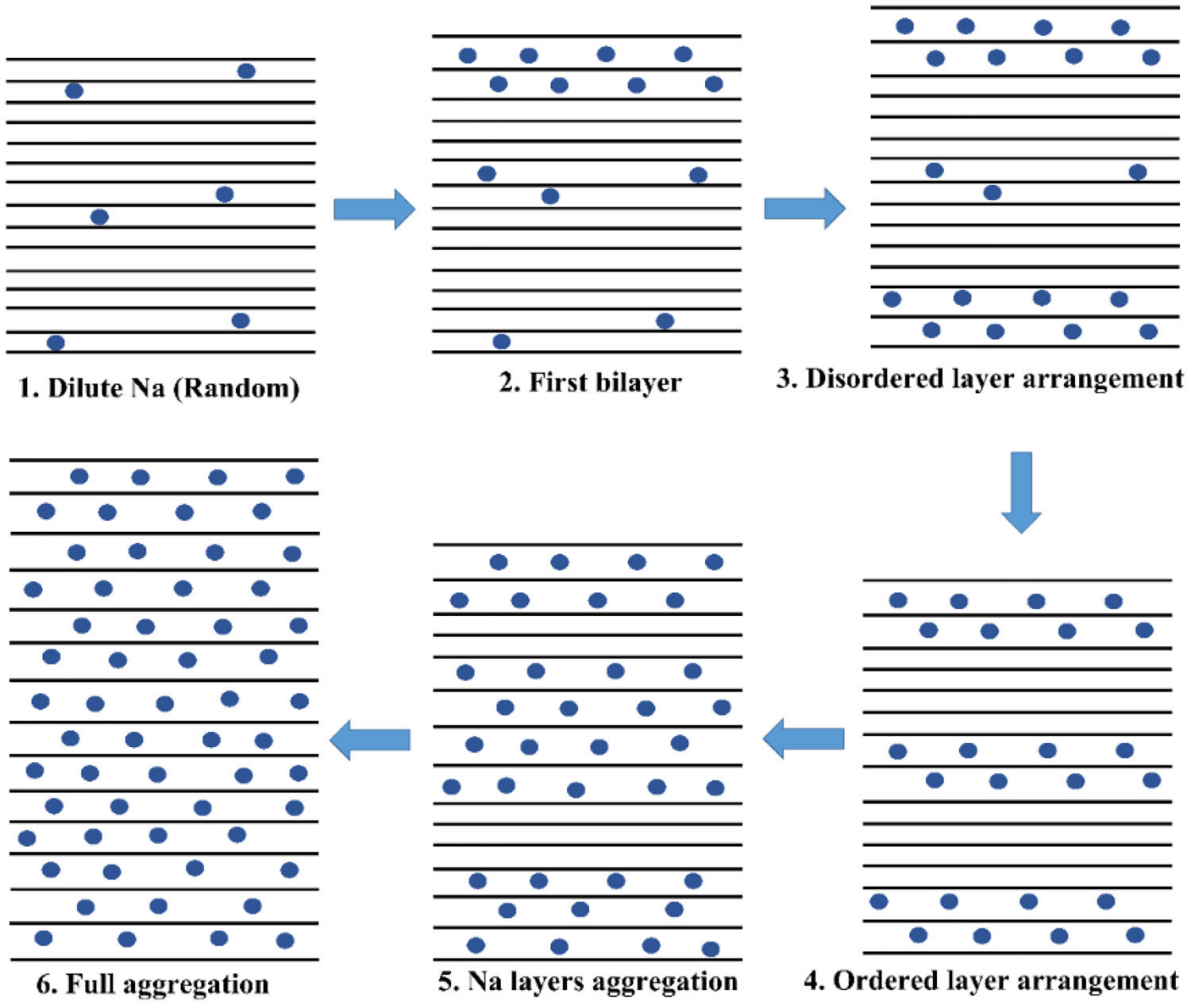
Sketch of the equilibrium intercalation pattern at increasing activity of Na (without defects consideration). Ignoring potential domain formation (see Figure 3a and ref. [42].), the figures can also be viewed as transient cases toward full aggregation at given high Na activity. If we replace the bilayers by monolayers, the sequence is applicable for the intercalation of the other alkali metals.

In this way we arrive at a consistent mechanistic picture of the intercalation process. Figure 5 gives a sketch of how we figure the formation of Na intercalation thermodynamically if the Na activity and hence the Na content is increased. It can also be interpreted as sketch of the kinetic intermediates (cf. LT insertion path). In the very beginning, a random distribution is expected. Soon, bilayers are favored whose distances become smaller and smaller, eventually favoring aggregates such as NaC_6_ or NaC_8_ for extreme and possibly unrealistic waiting times. Part SV‐5 calculates the energetic and entropic variations. An analogous feature can be applied to Li, K, Rb, Cs but by replacing bilayers with monolayers. The fact that we observe mostly homogeneously filled layers is due to the already discussed kinetic effect that the presence of alkali metal accelerates chemical diffusion.

#### 2.4.4 Stability of Bilayer Aggregates at Low Temperature

It remains to be explained why in the case of Na higher aggregates seem to be stable at LT (25°C), while at HT (400°C) almost exclusively chemical bilayers are observed. A tempting explanation would again be mass action considerations of the various aggregation degrees; but as already mentioned the configurational entropy for rearranging layers is much too small (as an example one may look at the annihilation of surfaces or grain boundaries: these energies are on the order of 0.1 or 1 J/m^2^ or more, exceeding configuration free enthalpy terms by many orders of magnitude, Part SV‐4). Hence, one has to seek the reason in the standard entropy of the aggregation reaction (i.e., the “local” entropy value), which we discuss now.

**FIGURE 6:**
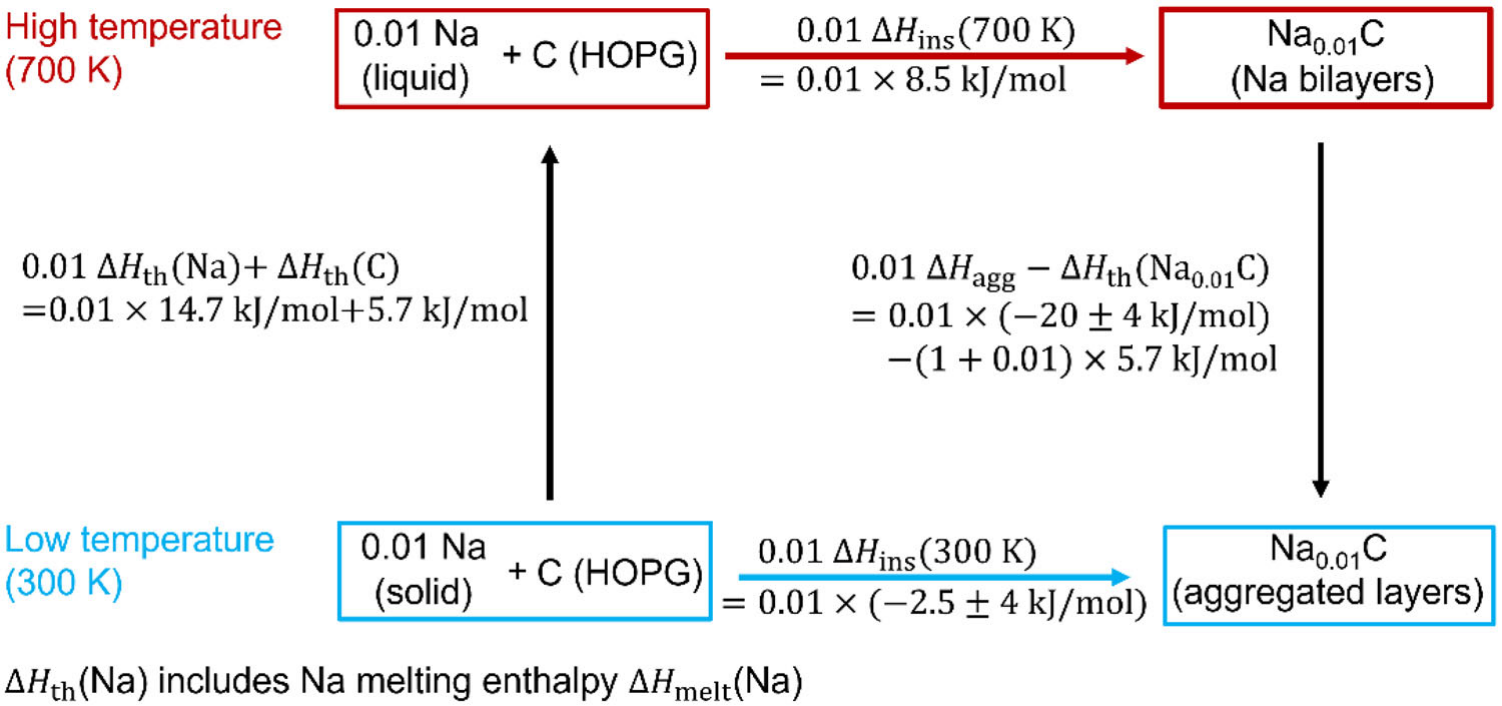
Thermodynamic cycle for the enthalpy changes as discussed in the text. The figure refers to the same *y* (0.01 mol Na) for HT and LT. Part SV‐5 gives a more general figure and shows that a difference in *y* (700 *K*) and *y* (300 *K*) does not perceptibly vary the results as long as *y* ≪ 1. The cycle shows the consistency of the values used.

Phononic effects do not appear sufficient to explain the behavior (the effective Debye temperatures for graphite and LiC_6_ or KC_6_ are similar and would only give rise to negligible entropy contributions [50, 64]). Yet space charge layers can (Figure 4c). Note that on aggregating two chemical bilayers to one quadruple layer—to select this example—one loses two space charge layers (per bilayer and hence for one Na per formula unit). As discussed above, related entropy effects can and actually do exceed in many cases the related energy effects. Then the standard free enthalpy referring to a layer or a layer aggregate would be given by $\Delta H_{\mathrm{agg}.}^{0}-T\Delta S_{\mathrm{agg}.}^{0}$ where the first term is dominated by a chemical interaction term (outrunning the small electric double layer value) while the second stems essentially from space charge entropy (outrunning phononic contributions). Note that elastic interactions, should they be important, would make the pure chemical bonding value even more negative. As shown in Part SV‐4 and 5, from the fact that the last term is dominant at HT, whilst overcompensated by the enthalpy term at LT, we can assess their values. The situation is sketched in Figure 6 (More details are found in Part SV). Figure 4a shows the van't Hoff plot of the solubilities versus temperature. The HT behavior refers to intercalation in bilayers and the LT behavior is closer to the intercalation into massive aggregates (agg.). According to the rough estimate derived in Part SV‐4, we can give $\Delta \;H_{\mathrm{agg}.}^{0}=\left(-20\pm 4.0\right)\;\mathrm{kJ}/\mathrm{mol}$, $\Delta \;S_{\mathrm{agg}.}^{0}=-34.0\;J/\mathrm{mol}\;K$, explaining the different slopes in Figure 4a along with the different nanostructures. Figure 4a shows in addition to the HT equilibrium Na concentrations, the extrapolated LT values. According to the experiments, the values show an upward deviation from the HT van't Hoff behavior. The insertion enthalpy corresponding to a LT van't Hoff slope—which is then to be understood as effective mean value—can be estimated to be (− 2.5  ±  4.0) kJ/mol (Part SV‐5). As this value is an average effective value, the equilibrium solubilities below 100°C may be substantially higher than those obtained by the linear ln [Na] versus 1/*T* approach and even values comparable to NaC_6_ or NaC_8_ may be thermodynamically possible though kinetically hardly realizable. The thermodynamic cycle depicted by Figure 6 shows the consistency of the thermodynamic arguments in terms of the respective enthalpies (For simplicity, in Figure 6 the same Na‐content was taken for HT and LT). In the Supporting Information, where refinements of the above Δ*H*‐cycle (Part SV‐5 and Figure SV‐6) are discussed, it is shown that a different Na content (*y*) for HT and LT would not change the result as long as *y* ≪ 1.

## 3 Summary and Conclusions

The present contribution is concerned with the intensely debated Na storage in graphite using long‐time chemical and electrochemical storage experiments on HOPG. With the help of a multitude of experiments, not only a comprehensive picture in terms of thermodynamics and kinetic is arrived at, also an unexpected storage feature is detected.

On increasing temperature from 25°C to 500°C the storage behavior changes from interfacial control to diffusion control and finally to thermodynamic control. The Na content of these equilibrated samples is found to be on the order of 1 mol %. Thermodynamically higher Na concentrations are predicted for LT, but are kinetically out of reach (given practically relevant waiting times). The OCV is small but positive indicating thermodynamically stable insertion at a Na‐activity of 1 (i.e., equilibrated with Na). The small OCV values imply small driving forces and can be held responsible for the sluggish kinetics.

The structure of these compositions is—at HT and/or for very low metal activity—surprisingly characterized by carbon‐layers that are embedded by Na layers (i.e., we face two‐sided intercalation) which are under‐occupied when compared to layers in AC_6_ or AC_8_. The further evolution of the intercalation pattern on increased Na activity can be understood by ordering of the bilayer sequence once the screening length is reached (the critical distance is typically on the order of 10 carbon‐layers). Further aggregation to denser bilayer arrangements or higher layer aggregates (lower staging number) requires lower temperatures. In principle, an analogous qualitative picture can be assumed for the other alkali metals with the exception that here monolayers play the decisive role before full aggregates are realized on subsequently reducing the number of pure carbon‐layers (reduction of staging number). Thus, by the inclusion of space charge layers and their interaction together with a self‐amplified chemical diffusion, a comprehensive mechanistic picture of alkali metal intercalation including the staging phenomena can be given.

The strikingly anomalous role of Nawithin the column of alkali metals regarding structure (bilayers instead of monolayers), storage capacity (extremely small), OCV (very close to zero) and kinetics (very sluggish) can be thermodynamically explained by the interplay of energy and entropy including space charges, and atomistically by the interplay of covalency, ionicity, and size [65, 66].

Interpreting the results in terms of battery research, it can be stated that for HOPG and then even more so for graphite powder, in order to reach the necessary storage in practically relevant times at room temperature, coated nanocrystalline samples are required. In view of the small OCV‐values which are not safer than the values of hard carbon, we can conclude that pure graphite is inferior to hard carbon with its much better kinetics and higher achievable capacities. The most promising path for optimizing graphite electrodes should lie in chemically modified carbon enabling better thermodynamics, in the preparation of kinetically stable carbon structures with a high content of higher‐dimensional defects (this includes high surface carbon), or in the preparation of high surface carbon whose surfaces are chemically modified.

## Author Contributions

C. G. and C. X. contributed equally to the work. C. G. performed the storage experiments, C. X. performed electrochemical experiments, H. W. and P. v. A. are responsible for the TEM measurements, R. M. did the chemical analysis, S. B. did the XRD simulations, B. V. L. and J. M. supervised the experiments and conceived the project. J. M. is responsible for the thermodynamics analysis. All the authors discussed the results together and assisted in writing the paper.

## Conflicts of Interest

The authors declare no conflicts of interest.

## Supporting information




**Supporting File 1**: anie71998‐sup‐0001‐SuppMat.pdf.

## Data Availability

The data that support the findings of this study are available from the corresponding author upon reasonable request.
