# Supplementary material for: Anomalous Sodium Insertion in Highly Oriented Graphite: Thermodynamics, Kinetics and Evidence for Two‐Sided Intercalation
Source: Angew Chem Int Ed Engl. 2026 Mar 28;65(19):e8139859. doi: 10.1002/anie.8139859 (PMC13134605; doi:10.1002/anie.8139859)
Supplement: Supplementary file 1 — Supporting File 1: anie71998‐sup‐0001‐SuppMat.pdf. [file ANIE-65-e8139859-s001.pdf]

SUPPORTING INFORMATION

Anomalous Sodium Insertion in Highly Oriented Graphite: Thermodynamics, Kinetics and Evidence for Two-Sided Intercalation

Chuanhai Gan[*1], Chuanlian Xiao[1], Hongguang Wang, Peter A. van Aken, Rotraut Merkle, Sebastian Bette, Bettina V. Lotsch, Joachim Maier*

Max Planck Institute for Solid State Research, 70569 Stuttgart, Germany

[1]These authors contributed equally.

*Corresponding authors, Chuanhai Gan (c.gan@fkf.mpg.de); Joachim Maier (office-maier@fkf.mpg.de)

An initial manuscript of the present work was uploaded to arXiv (preprint).[1]

Table of Contents

Part SI Methods and Materials

Part SII Na Concentration, Interface Stability, and Kinetic Analysis of CV Measurements

Part SIII Supplementary Figures

Part SIV XRD Simulations and Analyses

Part SV Entropy and Enthalpy Problems

Supplementary References

Appendix Consecutive STEM imaging

Part SI Methods and Materials

1 Materials Synthesis

Na intercalated graphite compounds (Na_yC) were synthesized by chemical and electrochemical intercalation of Na (99.9 %, Sigma-Aldrich) into uncoated and TiO_2 (or amorphous carbon, coating method is similar to ref.[2]) coated HOPG. Sample preparation for both chemical and electrochemical intercalation and post-treatment were in argon-filled glove box ($O_2 \leq 0.1$ ppm, $H_2O \leq 0.1$ ppm). As a rule, every piece of HOPG used was around 5×5×0.25 mm^3 by cutting commercial HOPG crystals (Mosaic angle 0.8±0.2°, Thermal conductivities 1800±200 w/mK in basal plane and 8±2 w/mK perpendicular to basal plane, size 10×10×1 mm^3, Thermo Fisher Scientific) without further treatment. TiO_2 coated samples were prepared by atomic layer deposition (ALD) method in clean room. Tetrakis-dimethylamido-titanium (TDMAT) and H_2O were used as the precursors ($Ti(NMe_2)_4$ (g) + $2H_2O$ (g) → TiO_2 (s)+ $4HNMe_2$ (g)). Uniform TiO_2 coatings of different thicknesses were obtained by varying the number of cycles. The optimized deposition conditions and rates were given in ref.[3]

Chemical intercalation of Na (direct contact of HOPG with Na) into uncoated and coated HOPG (or uncoated polycrystalline graphite powder) was conducted at low/high temperatures (25-500 °C) at given time, Fig. SI-1(a, c). High temperature (HT)-synthesized samples were heated to given temperatures with the rate of 10 °C/min if without specific statement. After synthesized, samples were quickly cooled to room temperature under an argon atmosphere (i.e. quenching, less than 20 min.). For a reference comparison, slow oven cooling of 6 °C/h was also investigated. Routinely, quenched samples were taken for further investigation. Electrochemical intercalation of Na into uncoated and coated HOPG (electrolyte 1 M $NaPF_6$-EC/DMC (vol. 1:1) if without specific notice) was conducted at 25 and 40 °C by constant current discharge from OCV to 0 V (vs Na metal), followed by a hold at 0 V vs Na (voltage fluctuated between 0 and 1 mV, Arbin system BT 2000) for the specified duration. Li (electrolyte 1 M $LiPF_6$-EC/DMC (vol. 1:1), Aldrich) and K (electrolyte 1 M KPF_6-EC/DMC (vol. 1:1)) cases were similarly conducted. Polycrystalline graphite powder (D_{50}=11 µm, 99.95%, NGS Naturgraphit GmbH) was assembled as battery as usual, followed by investigating as similar to HOPG case, but negative voltages were also explored.

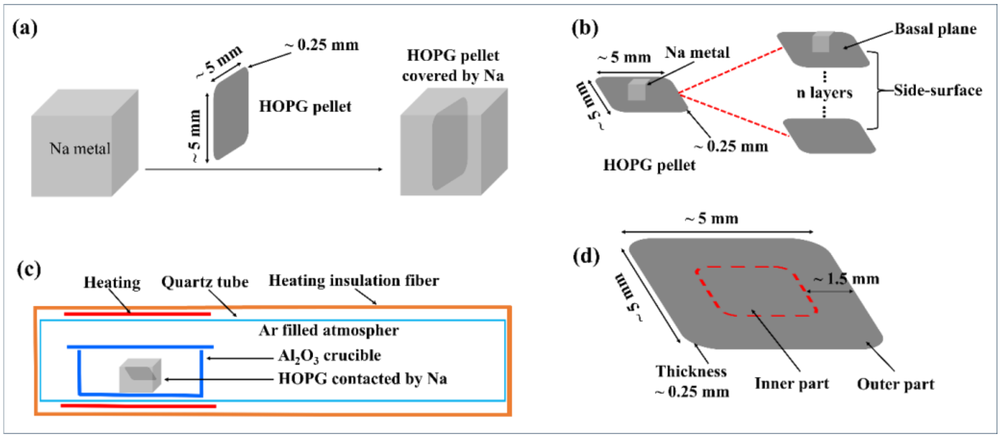

Fig. SI-1. Experimental schemes of 25 °C chemical intercalation (a) by fully covering HOPG pellet using Na metal, (b) by attaching Na metal to the basal plane of HOPG pellet. (c) Schematic set-up for high temperature chemical intercalation by fully covering HOPG pellet using Na metal. (d) Experimental schemes for separating inner and outer parts of synthesized sample.

2 Kinetic and thermodynamic investigations by CV, OCV and XRD

For evaluating the kinetics of the electrochemical intercalation of Na into HOPG, cyclic voltammetry (CV) measurements of Na intercalation into (deintercalation from) uncoated and TiO_2 coated HOPG (10×10×1 mm^3) were performed using a Universal Pulse Dynamic-EIS Voltammetry (Voltalab, PGZ402) at 25 °C (see Fig. 1 (c, d, e)). Sweep rates ranging from 10 µV/s to about 10 V/s were applied with a potential window from 0 to 2.5 V in this study. For a reference comparison, equally sized TiO_2 coated on Nb-doped $SrTiO_3$ (10×10×1 mm^3) was also investigated by CV.

Potential (voltage) recordings (OCV) were carried out as a function of time to evaluate kinetics of Na deintercalation and formation free energy of Na_yC. For Na_yC chemically obtained at high temperatures, after cooled down to 25 °C, followed by cleaning their surface in an argon-filled glovebox, then samples were assembled as coin cell batteries to record voltages vs. Na; For Na_yC electrochemically obtained at 25 °C voltages were directly recorded without reassembling batteries.

Apparent diffusion coefficients (D_{Na}^{δ}) of (electro)chemical intercalation of Na into HOPG at high/low temperatures were estimated by XRD measurements on inner and outer parts (Fig. SI-1(d)) of the chemically and electrochemically synthesized samples Na_yC. D_{Na}^{δ} can be estimated by $L^2 \simeq 2D_{\mathrm{Na}}^{\delta}t$ (where L is diffusion length and t diffusion time).

3 Materials Characterization

3a XRD

Measurements were conducted under Bragg-Brentano configuration using programmable divergence slits, anti-scatter slits and a PIXcel 3D detector (PANalytical Empyrean, CuKα radiation). Samples were sealed in a polycarbonate domed holder before measurement. For data processing and analysis (diffraction), PANalytical software packages HighScore Plus were used.

3b Na concentration measurements - Chemical analyses

After synthesis, samples were cleaned by scratching residual Na and other impurities on the surface. Acid-base titration routinely as well as ICP-OES (not routinely) was used to determine the overall Na concentration. For both techniques we used aqueous extractions of one sample, transforming the Na into NaOH. The sample (typically 5-20 mg) was placed in a Schlenk flask with 80 ml of freshly prepared bi-distilled water, and heated for approx. 20 h to 90 °C (under argon atmosphere). Under these conditions the sodium excorporates from the graphite and reacts according to $Na + H_2O \rightarrow Na+ + OH^- + 0.5\ H_2$. The formed OH^- was titrated with 0.01n HCl (Alfa Aesar) using a Metrohm Titrino 877 automatic titrator (sample solution still under Ar atmosphere to avoid reaction with CO_2); the amount of Na^+ directly corresponds to the titrated OH^- amount. For samples with very low Na content, this method was not applicable because after heating the sample with bi-distilled water the pH was higher than for the pure water but still below 7 (bi-distilled H_2O made from water coming from a cation exchanger is still slightly acidic). In these cases, the pH of the pure bi-distilled water and of the sample solution was measured, and the amount of Na^+ calculated from the decrease of OH^- concentration (and sample solution volume).

3c STEM and EELS

Experimental details on Scanning transmission electron microscopy (STEM) sample preparation is shown in ref.[4] STEM studies were conducted using a spherical aberration-corrected STEM (JEM-ARM200F, JEOL Co. Ltd.) equipped with a cold-field emission gun and a DCOR probe Cs-corrector (CEOS GmbH) operated

at 200 kV. The high-angle annular dark-field (HAADF-STEM) images were obtained by an ADF detector with a convergent semi-angle of 20.4 mrad and collection semi-angles of 70−300 mrad. In order to make precise measurements of lattice constants, six serial frames were acquired with a short dwell time (2 μs/pixel), aligned, and added afterward to improve the signal-to-noise ratio (SNR) and to minimize the image distortion of STEM images. electron energy loss spectroscopy (EELS) acquisition has been performed with a Gatan GIF Quantum ERS imaging filter equipped with a Gatan K2 Summit camera and a CCD camera with a convergent semi-angle of 20.4 mrad and a collection semi-angle of 111 mrad. An image processing pipeline was developed to identify intercalated areas to enable statistical analysis of Na intercalation in HOPG.

3d XPS

X-Ray photoelectron spectroscopy (XPS) measurements were performed on a Kratos Axis Ultra system equipped with a monochromatized Al K_α X-Ray source. Samples were prepared and transferred to the XPS chamber in an airtight transfer tool in an Ar-filled glove box. After measurement of sample surface, for depth profiling, a scanning MiniBeam III Argon ion sputter gun (from Kratos) with a beam energy of 4.0 keV and an emission current of 20 mA was used (1, 3, 8, 16, and 30 min). An immediate measurement was performed after each sputtering.

3e SEM and EDX

The Scanning Electron Microscopy (SEM) micrographs were obtained with a Zeiss Merlin SEM operating at a voltage of 4 kV. Energy Dispersive X-ray Spectroscopy (EDX) mapping was done with an Oxford Instruments detector (Ultim Extreme).

3f EIS

Electrochemical Impedance Spectroscopy (EIS) was employed to characterize the interfacial stability at room temperature. Electrochemcially intercalated samples were synthesized as described in Section 1). Measurements were performed using a Novocontrol Alpha-A analyzer (Novocontrol Techologies, Germany, 2-wire measurement, 10^6 to 10^{-3} Hz, amplitude 0.01 or 0.05 V).

Part SII Na Concentration, Interface Stability, and Kinetic Analysis of CV Measurements

1 Na-compositions of samples for different storage procedures

1-1 Table SⅡ-1 Na intercalated HOPG composition (NaC_x) by titration after hydrolysis [a, b]

Time / (°C) Temperature	2 days	1 week	2 weeks	3 weeks	4 weeks	8 weeks	> 8 weeks	Average
500 (chemically)			NaC_{90}					NaC_{90}
400 (chemically)	NaC_{91}<br>NaC_{88}<br>NaC_{148}<br>NaC_{172}<br>NaC_{119}<br>NaC_{105}[c]<br>NaC_{115}[d]		NaC_{106}<br>NaC_{115}<br>NaC_{102}<br>NaC_{96} (outer)<br>NaC_{153} (inner)		NaC_{109}			NaC_{122}
300 (chemically)			NaC_{133}<br>NaC_{135}<br>NaC_{135}					NaC_{134}
250 (chemically)	NaC_{216}<br>NaC_{419}	NaC_{163}<br>NaC_{156}	NaC_{133}<br>NaC_{135}	NaC_{209} (outer)<br>NaC_{395} (inner)			NaC_{138} (outer)<br>NaC_{158} (inner)<br>(2.5 months)<br>NaC_{193}<br>(5 nm TiO_2 coated, 2.5 months)	NaC_{180}
200 (chemically)			NaC_{358}<br>NaC_{194}<br>NaC_{194}<br>NaC_{185}<br>NaC_{132}	NaC_{290}<br>NaC_{315}<br>(5 nm TiO_2 coated)			NaC_{122} (outer)<br>NaC_{191} (inner)<br>(2.5 months)<br>NaC_{109} (outer)<br>NaC_{139} (inner)<br>(5 nm TiO_2 coated, 2.5 months)	NaC_{152}
180 (chemically)			NaC_{276}<br>NaC_{171}<br>(5 nm TiO_2 coated)					NaC_{224}
150 (chemically)			NaC_{239} (outer)<br>NaC_{14660} (inner)			NaC_{359} (outer)<br>NaC_{374} (inner)	NaC_{279} (outer),<br>NaC_{249} (inner); 6 months	
			NaC_{16225}[e]<br>(5 nm TiO_2 coated)			NaC_{178}<br>(5 nm TiO_2 coated)	NaC_{109}, 6 months<br>(5 nm TiO_2 coated)	
100 (chemically)			NaC_{12266}[e]<br>NaC_{10269}[e]					
			NaC_{14263}[e] (outer)<br>NaC_{17823}[e] (inner)<br>(5nm TiO_2 coated)					
25 (chemically)			NaC_{15200}[e]<br>NaC_{11850}[e]				NaC_{13861}[e] (1 year)	
			NaC_{13700}[e]<br>(2 nm TiO_2 coated)				NaC_{18315}[e] (1 year)<br>(2 nm TiO_2 coated)	
40 (electrochemically, 5 nm TiO_2 coated)			NaC_{666}		NaC_{133} (outer)<br>NaC_{1115} (inner)		NaC_{109} (outer)<br>NaC_{151} (inner)<br>4 months	
25 [f] (electrochemically)		NaC_{10650}[e]<br>(outer)<br>NaC_{7740}[e]<br>(inner)	NaC_{539}			NaC_{276}	NaC_{19320}[e]<br>(3 months)	
25 (electrochemically, TiO_2 coated)			NaC_{664} (2 nm TiO_2)			NaC_{362}<br>(2 nm TiO_2)	NaC_{573}[g] (2 nm TiO_2)<br>NaC_{904}[g] (2 nm TiO_2)<br>(3 months)	
			NaC_{13040}[e] (5 nm TiO_2)			NaC_{466}<br>(5 nm TiO_2)	NaC_{287}<br>(5 nm TiO_2, 3.5 months)<br>NaC_{251}<br>(5 nm TiO_2, 4 months)	

Note:

a) Estimated error bar: ±10%; Original HOPG: NaC_{100000}.

b) Note that the time elongation of intercalation may also be influenced by corrosion. This may be the reason for non-monotonicity of concentration, e.g. 200 °C cases.

c) This sample was cooled from 400 °C to room temperature with a temperature decrease rate of 6 °C/h (we also tested a parallel sample, the composition of which was measured to be NaC_{116}, i.e. no big difference). These two concentrations (NaC_{105} and NaC_{116}) were not included in the averaging. Routinely, samples were fast cooled to room temperature within 20 min. in argon atmosphere.

d) Sample was tested by ICP-OES. In ICP-OES analysis, the dissolved sample was introduced into the inductively generated argon plasma via an atomizer system and excited. The emitted radiation was transferred to the ICP spectrometer where it was split into the individual wavelengths and evaluated. The intensities of the spectral lines were measured with CCD semiconductor detectors. Calibration was carried out with Na solution mixed from standard solutions.

e) Calculation based on pH for x>2000. It has to be noted that these cases come with a larger uncertainty of the Na content. It has to be noted that this calculation assumes that the base formed as reaction product of Na with water if fully dissociated; if this does not hold then the Na content determined for this analysis is underestimated.

f) Solvent co-intercalation probably occurs due to unstable interfaces (see Part Ⅱ-2).

g) 2 nm TiO_2 coated samples under prolonged time (eg. > 8 weeks) would result in Na concentration decrease, which is attributed to unstable interfaces.

1-2 Na under-occupancies of chemically sodiated 400 °C sample

As theoretical stoichiometry is, for Na intercalated graphite, NaC_8 or NaC_6 are discussed in the literature.[5] According to TEM data (see Fig. 3 (a) of the main text and Fig. SⅢ-23), more than 70 % of the intercalates are in Na bilayers, 0 % in monolayers, the rest is in trilayers or higher layers aggregates. Empty (non-intercalated) carbon layers in-between intercalated Na layers are typically 4-10 layers in average.

Let us assume full occupancy, i.e. Na intercalated carbon layers are in the form of NaC_8 (or NaC_6), with *n* non-intercalated carbon layers in between (Fig. SⅡ-1).

a) If we assume *n* is 7, non-intercalated carbon layers in between intercalated Na layers in an ideal unit cell, then we end up with stoichiometry of NaC_{36}, which is distinctly less than the value from analysis, corresponding to ~NaC_{100} (Table SⅡ−1 above). A composition ~NaC_{100} would imply having 20 carbon layers in between which exceeds the average value by about a fact of 2.

b) If we also take account of the fact that we have higher aggregates or higher-dimensional defects where Na adsorbed (cavities, triple-phase junctions, etc.), the Na-under occupation in bilayers would be even more significant.

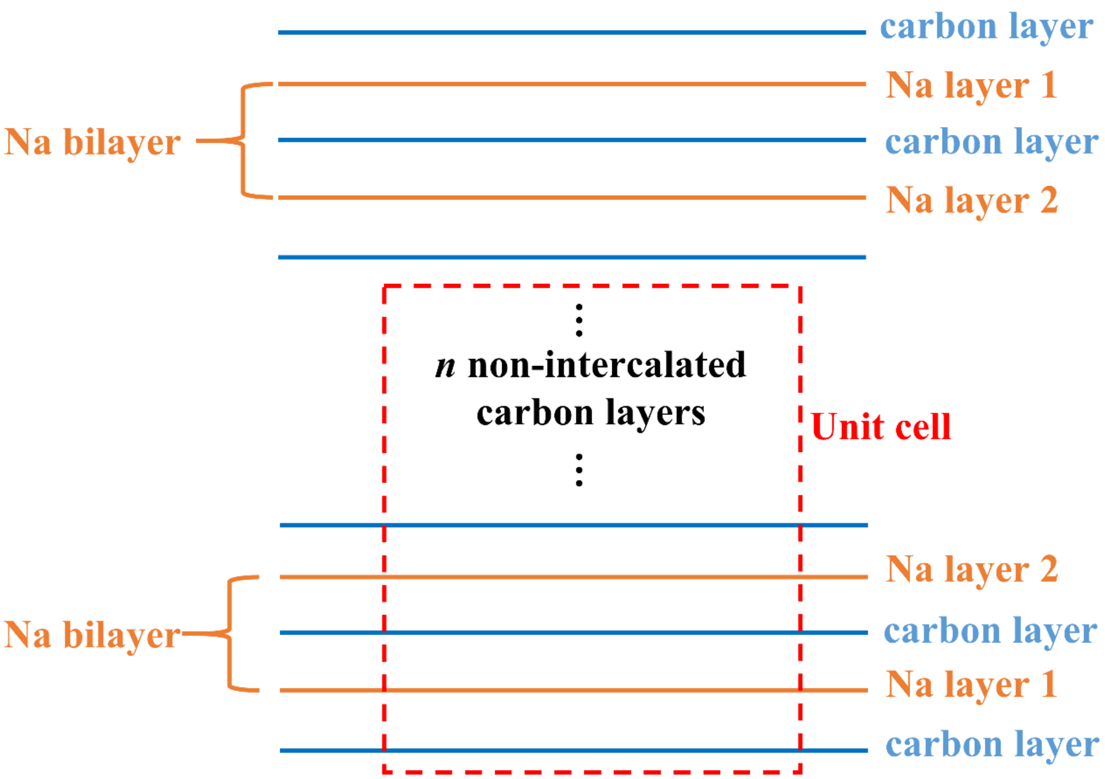

Fig. SⅡ-1 Sketch of Na intercalated HOPG

2 Interface stability and TiO_2 coating for electrochemical storage

Since the interface of Na-containing HOPG-electrolyte is unstable, TiO_2 coating is used to improve the interface stability of the HOPG pellet. The following consideration shows that TiO_2 would not react with Na-containing HOPG. It suffices to show that it is stable even against Na.

$\mathrm{TiO_2 + 4Na \rightleftharpoons Ti + 2Na_2O}$

Using

$\Delta G^{\circ}_{\mathrm{TiO_2}} = -889\ \mathrm{kJ/mol}$[6]

$2\Delta G^{\circ}_{\mathrm{Na_2O}} = 2 \cdot (-379)\ \mathrm{kJ/mol} = -758\ \mathrm{kJ/mol}$[6]

we see that

$2\Delta G^{\circ}_{\mathrm{Na_2O}} > \Delta G^{\circ}_{\mathrm{TiO_2}}$

and $\Delta G^{\circ} (\simeq \Delta G)$ of the redox reaction is positive.

3 Kinetic analysis of CV measurements

Cyclic voltammetry (CV) is a popular technique for obtaining information about complex electrode reactions over a wide potential range in a fairly rapid fashion. Usually, the potential is linearly varied with time, i.e., is characterized by a constant sweep rate. After having reached the maximum potential, the process is symmetrically reversed.

Useful thermodynamic and kinetic information can then be obtained by analyzing the measured current-voltage relation. The mathematical analysis of the sweep experiments was first solved by Randles and Sevcik.[7] The obtained Randles-Sevcik equation is often used to predict the peak current as a function of sweep rate for a reversible reaction in a semi-infinite situation.

$$I_p = 0.4463(\frac{F^3}{RT})^{1/2} n^{3/2} A D_0{}^{1/2} C_O{}^* v^{1/2} = KnFAC_O{}^*(\frac{nFvD_0}{RT})^{1/2}$$

where I_p is the peak current, K is a constant, n is the number of electrons transferred in the redox reaction, A is the area of electrode, F is Faraday constant, D_0 is diffusion coefficient, $C_O{}^*$ is the concentration and v is the sweep rate.

The dependence of the peak current on sweep rate is often investigated in order to estimate kinetic parameters. When the kinetics are limited by diffusion, there is consensus that a power law relation should be valid: $I_p \propto v^b$, with $b = 0.5$.

For a sample of finite size (the sample size is very small or the time-scale of a measurement is long enough), the diffusion profile reaches the other side of the sample and the semi-infinite linear diffusion model is no longer valid. Then the theory of linear sweep voltammetry with finite diffusion space should be considered following the work of Aoki et al.[8] An approximate expression of peak current as a function of sweep rate using the boundary conditions in the system of finite space can be obtained as:

$$I_p = 0.4463\left(\frac{C_O{}^* D_0 nF}{d}\right) w^{1/2} \tanh\left(0.56\, w^{1/2} + 0.05\, w\right)$$

where d is the thickness of diffusion space and $w = (nF/RT)vd^2/D$. The additional term $\tanh\left(0.56\, w^{1/2} + 0.05\, w\right)$ is the only difference in comparison to semi-infinite diffusion. When the films are thick and sweep rate is large, $\tanh\left(0.56\, w^{1/2} + 0.05\, w\right)$ approaches 1 and the equation becomes identical to the semi-infinite diffusion case.

The exponent in $I_p \propto v^b$ is expected to change from $b = 0.5$ to 1.0 as sweep rate decreases.

The situation changes for surface reaction limitation will be shown elsewhere (C. Xiao, R. Usiskin, J. Maier, in preparation). The typical exponent will then be distinctly lower.

Part SIII Supplementary Figures

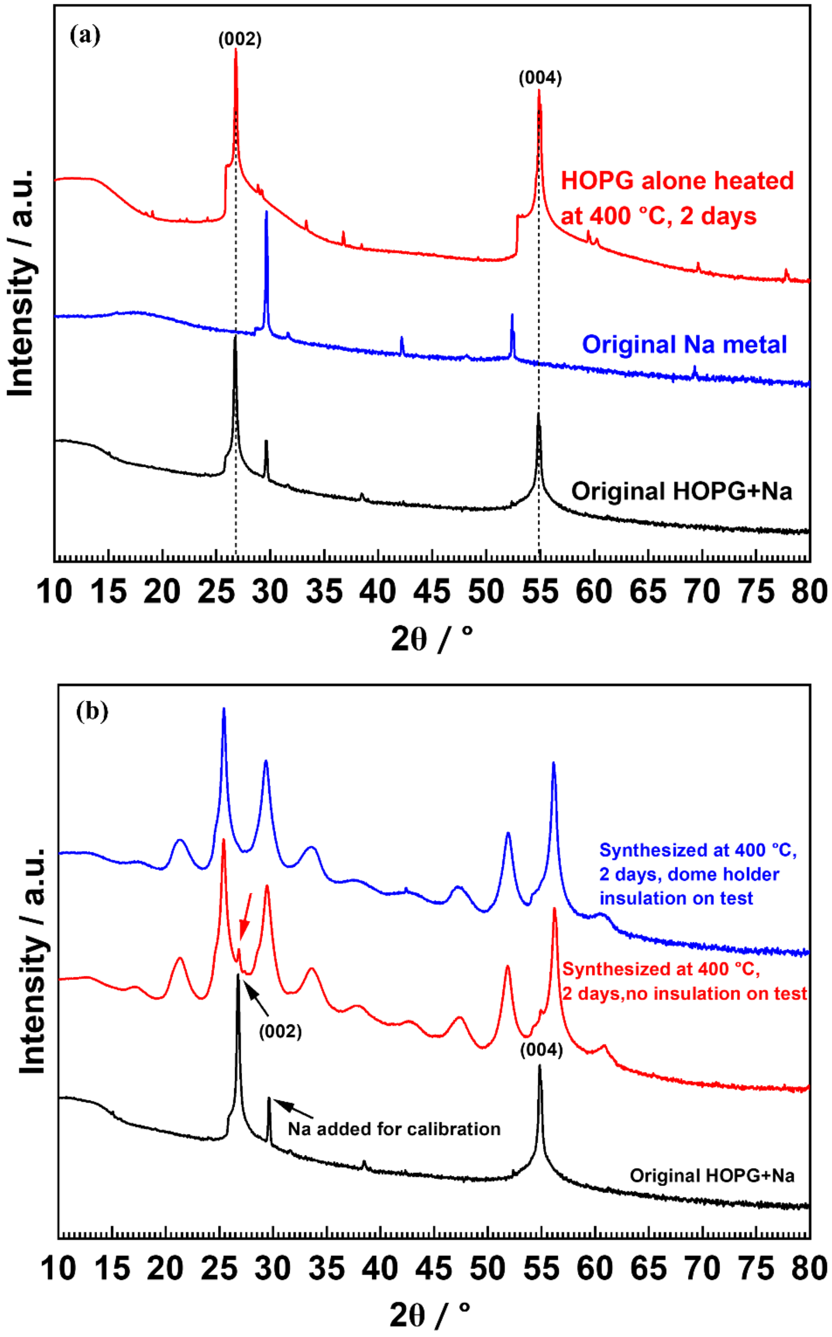

Fig. SⅢ-1 XRD patterns for (a) original Na metal, original HOPG plus Na metal, and pure HOPG after heating treatment at 400 °C of 2 weeks, (b) Na intercalated HOPG samples exposed to / insulated from air during test (Without dome holder insulation during test, there was a weak graphite signal of (002) as marked by red arrow).

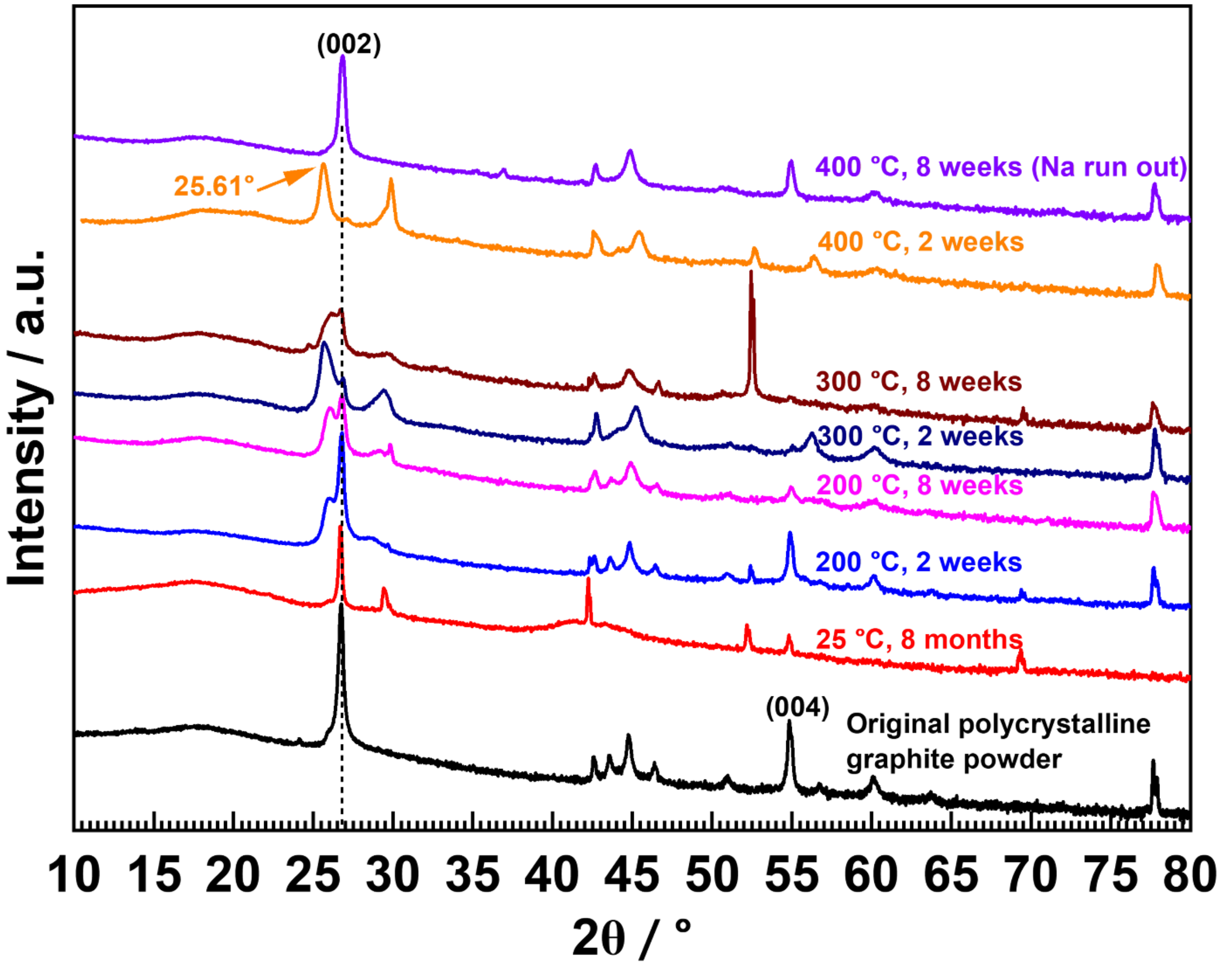

Fig. SⅢ-2 XRD patterns for chemical intercalation of Na into polycrystalline graphite powder (Na metal was added intentionally for the peak position calibration).

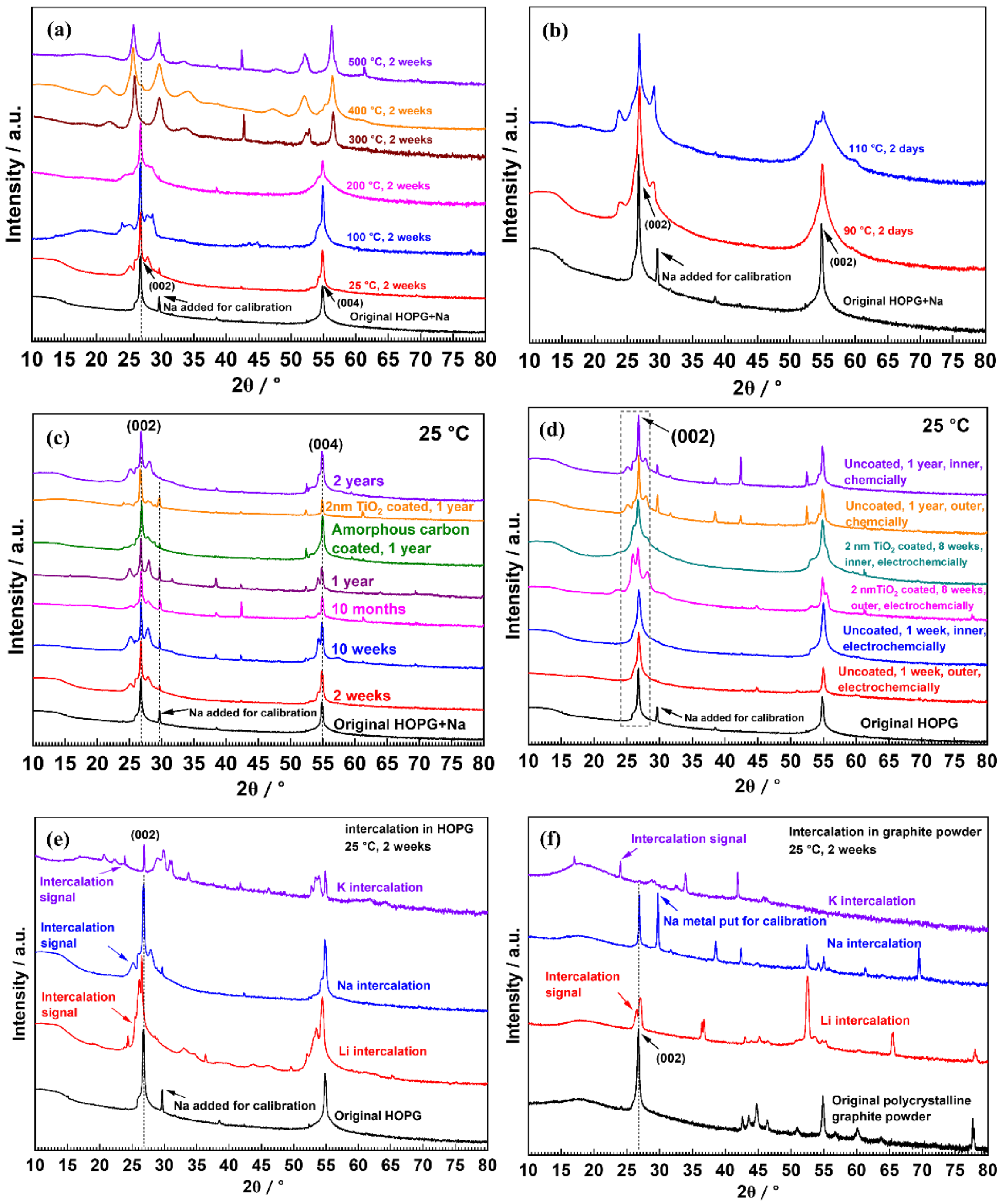

Fig. SⅢ-3. XRD patterns for chemical intercalation of Na into (a, b) HOPG at various temperatures with given time, (c) uncoated and coated (TiO_2 or amorphous carbon) HOPG at 25 °C, (d) chemically and electrochemically Na intercalated compounds for comparing the inner part and outer part of sample at 25 °C (dashed grey box shows intercalation signals at lower and higher angles compared to pure (002) peak of HOPG), chemically Li, Na and K intercalation into HOPG (e) and polycrystalline graphite powder (f) for 2 weeks at 25 °C. Na metal was added for the peak position calibration.

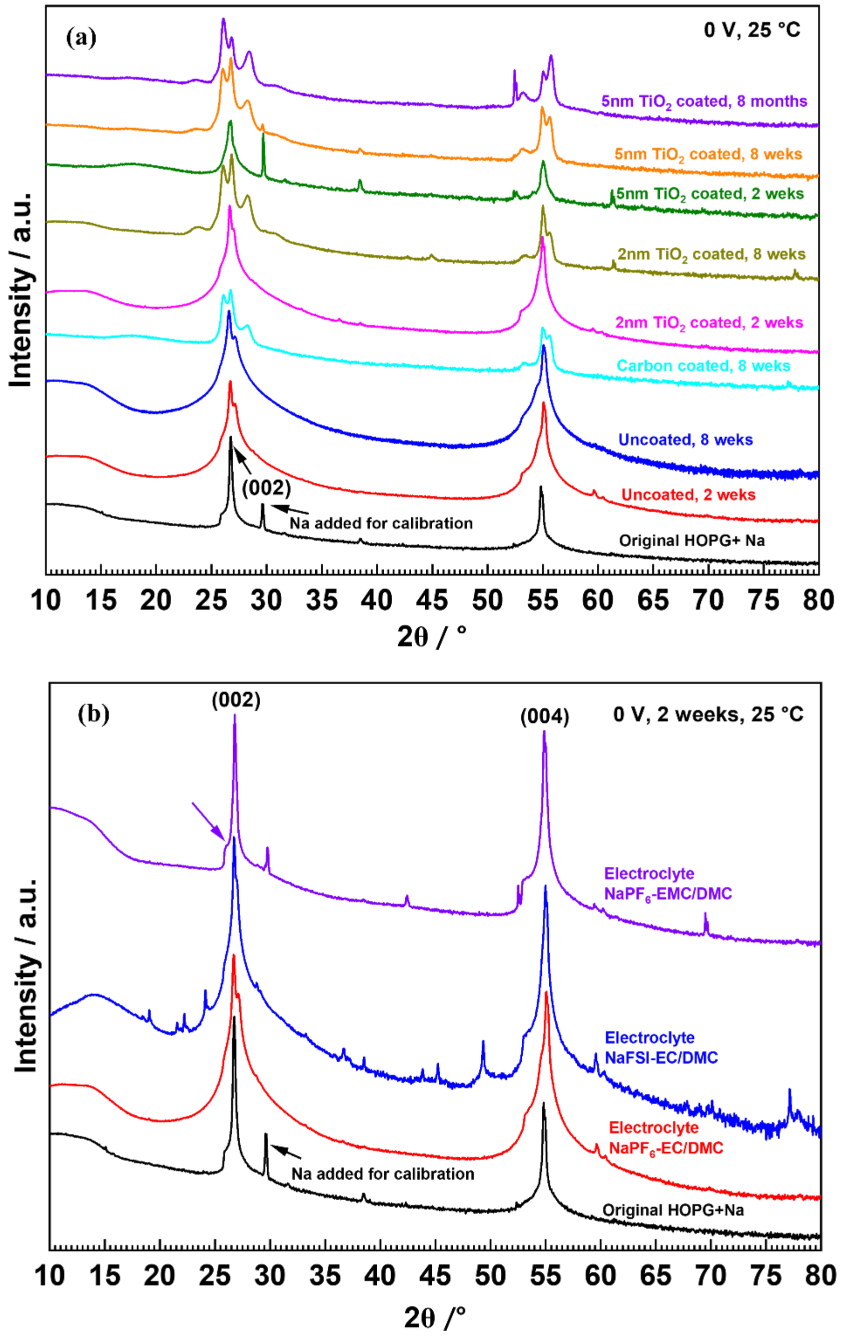

Fig. SⅢ-4 XRD patterns for electrochemical intercalation of Na into (a) TiO_2 or amorphous carbon coated HOPG at 0 V vs Na metal at 25 °C, (b) HOPG with different electrolytes for 2 weeks. Note that Na metal was added for the peak position calibration; the violet pattern (using $NaPF_6$-EMC/DEC electrolyte) indicates absence of observable intercalation.

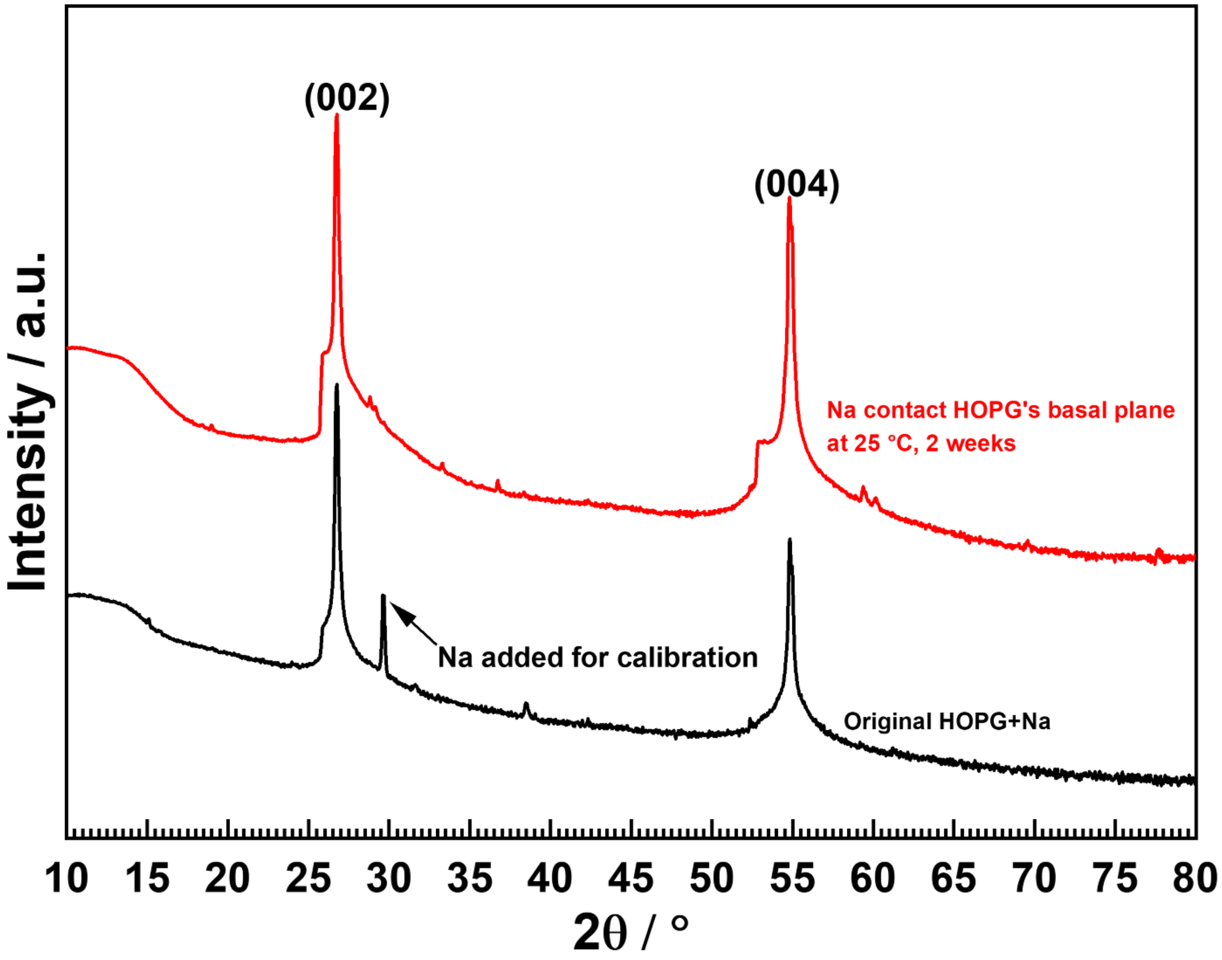

Fig. SⅢ-5. XRD patterns of direct contact of Na on the basal plane of HOPG at 25 °C for 2 weeks (chemical insertion, see Fig. SI-1(b)). Na metal was added for the peak position calibration.

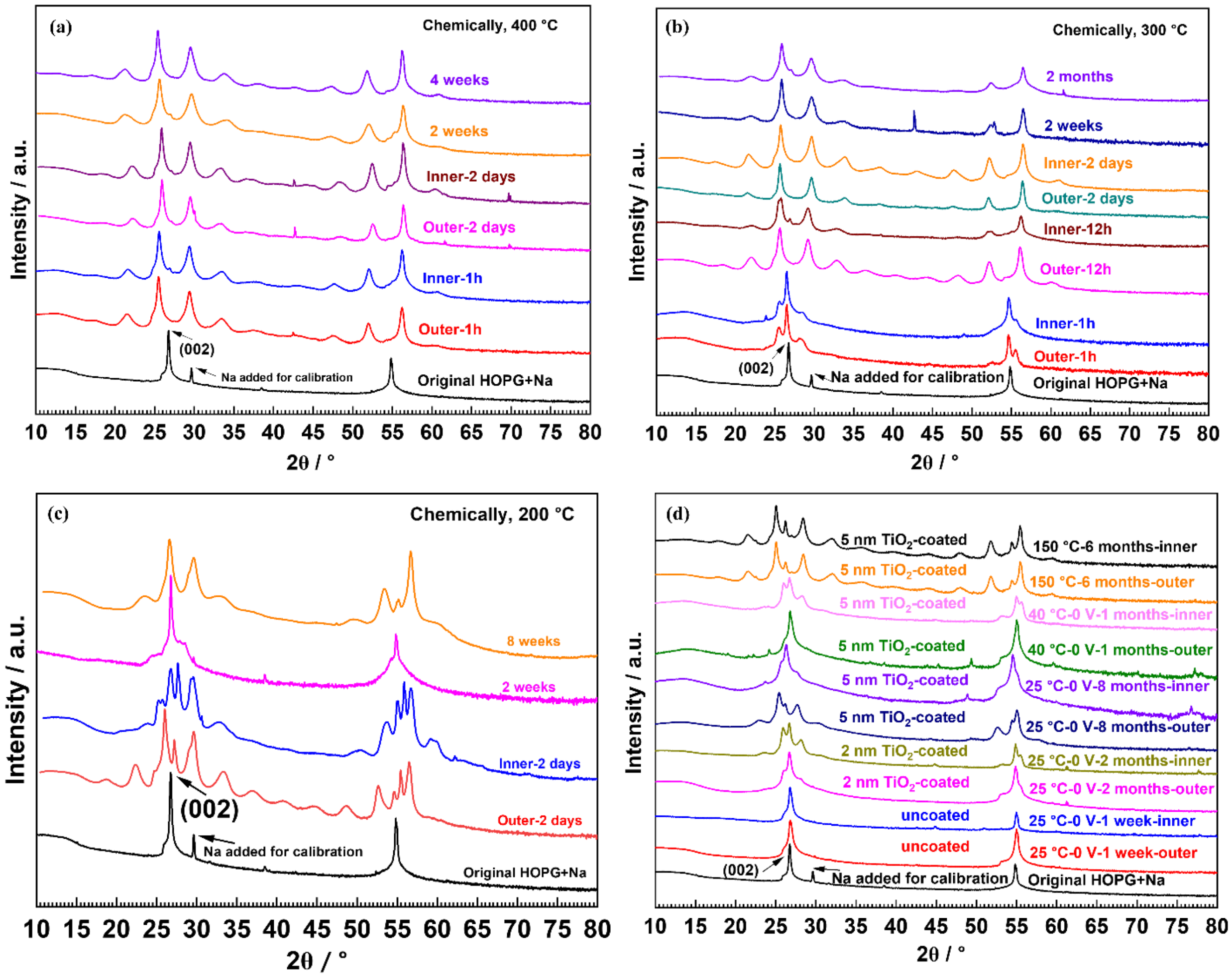

Fig. SⅢ-6. XRD patterns for chemical intercalation of Na into HOPG at (a) 400 °C, (b) 300 °C, (c) 200 °C and (d) 150, 40 and 25 °C, respectively (25 and 40 °C samples were synthesized by electrochemical method.) Inner and outer indicate inner and outer parts of sample were tested, respectively, as shown in Fig. SI-1d. Na metal was added for the peak position calibration.

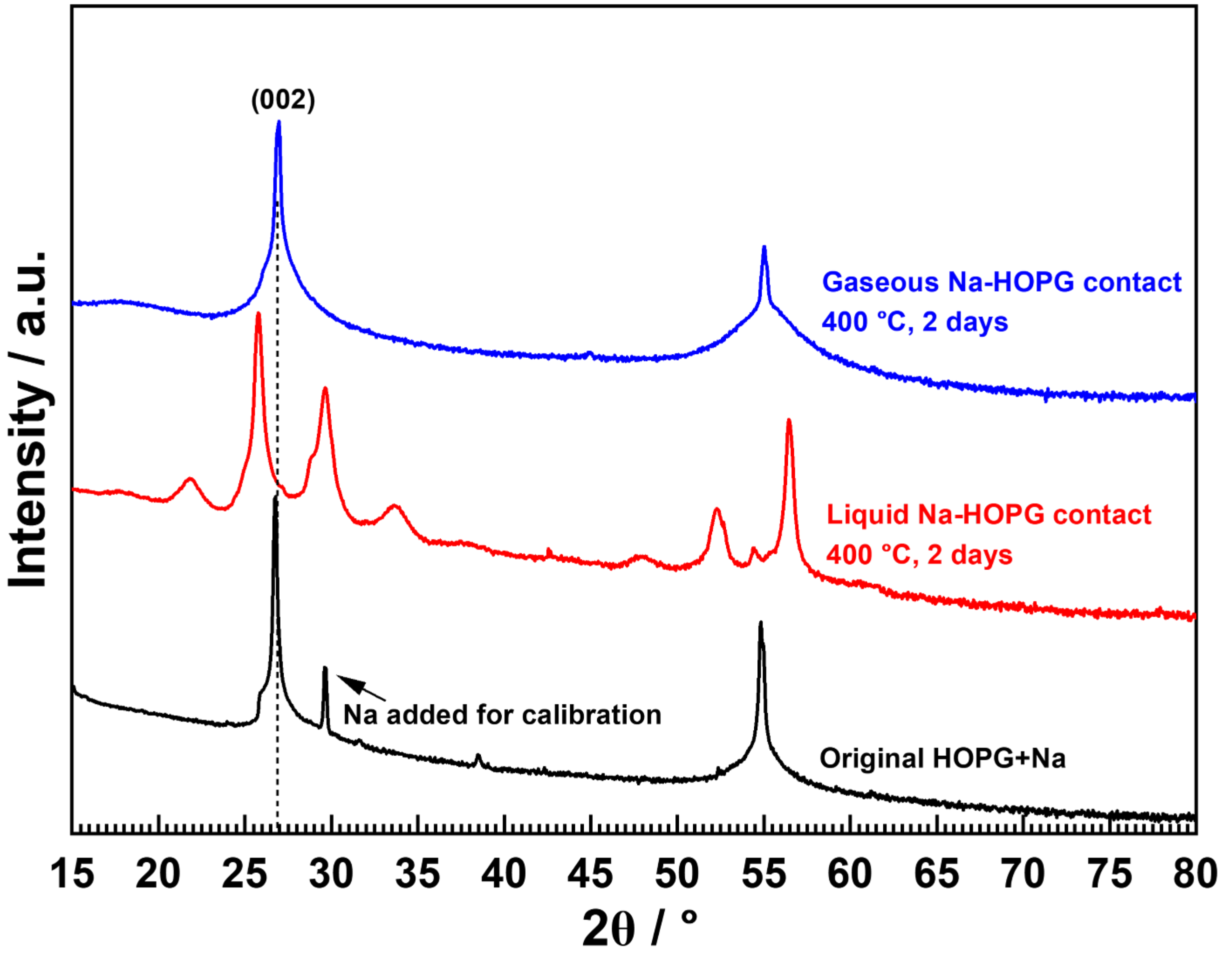

Fig. SⅢ-7. XRD patterns comparison for chemically gaseous and liquid Na intercalation into HOPG at 400 °C for 2 days. Na metal was added for the peak position calibration.

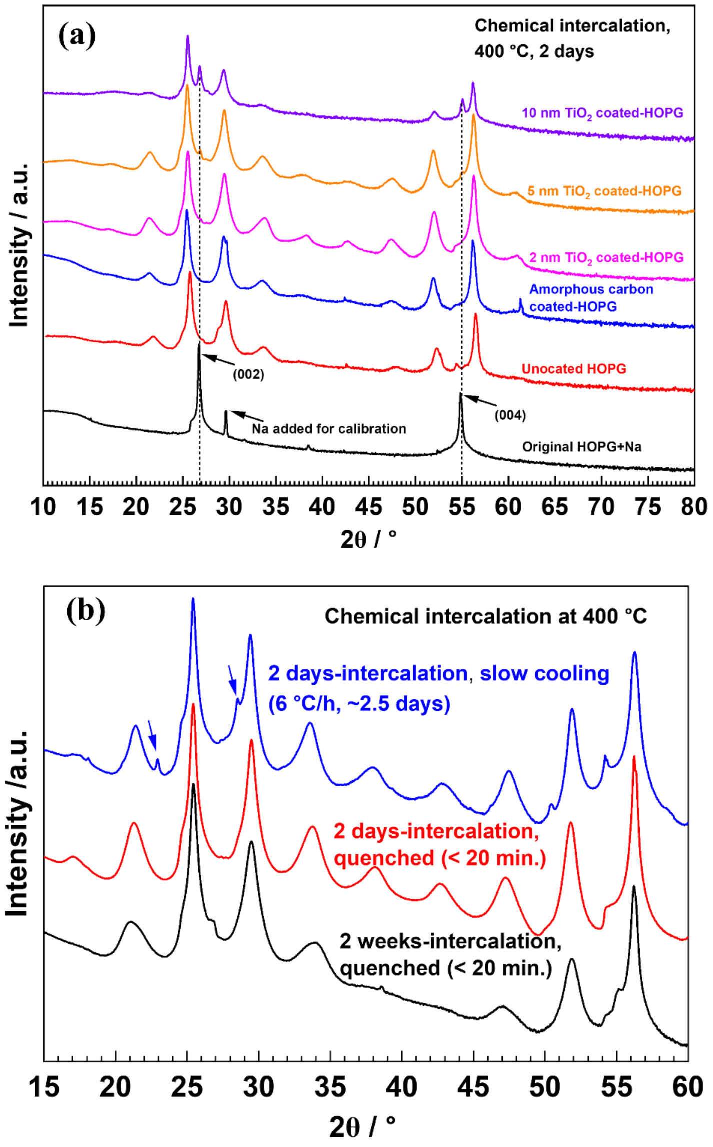

Fig. SⅢ-8. (a) XRD patterns for chemical intercalation of Na into uncoated-, amorphous carbon coated-, and TiO_2 (of various thicknesses) coated- HOPG at 400 °C for 2 days. Noting that the (002) graphite peak still exists for the thicker TiO_2 coated sample (Na metal was added for the peak position calibration). (b) XRD patterns for chemical intercalation of Na with different cooling methods. Only weak peaks (marked by blue arrows, which we ascribe to surface effects) appearing for samples of 6 °C/h cooling compared to that of quenched samples (cooled to room temperature in argon atmosphere within 20 min.); their Na contents are similar (~NaC_{100}, see Table SII-1), indicating that even slowing cooling can preserve the main characteristics.

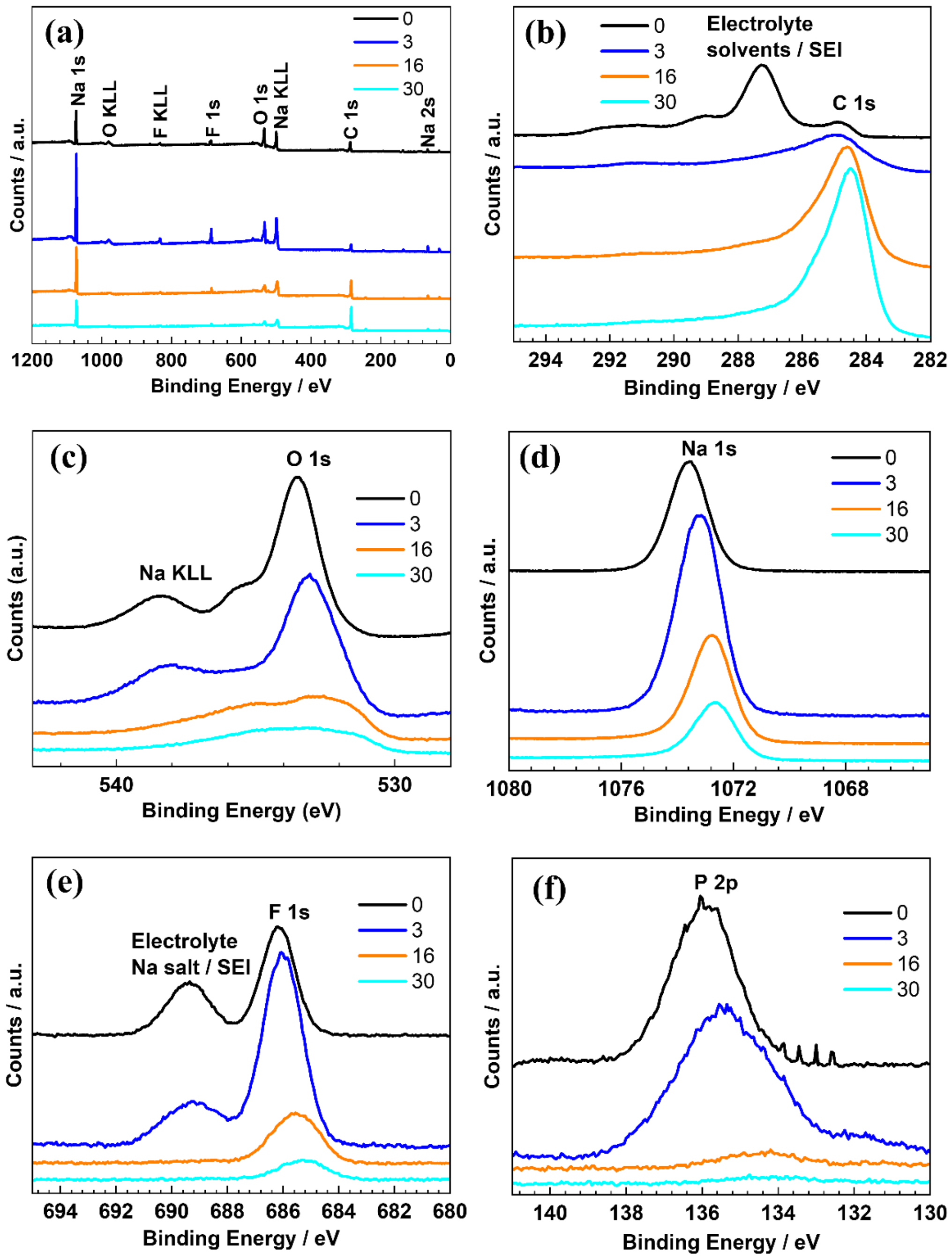

Fig. SⅢ-9 XPS spectrum for the electrochemical intercalation of Na into HOPG at 0 V vs Na with a waiting time of 3 days at 25 °C (The numbers 0, 3, 16 and 30 indicate Ar^+ sputtering time in minute, i.e. corresponding detected depth from sample surface).

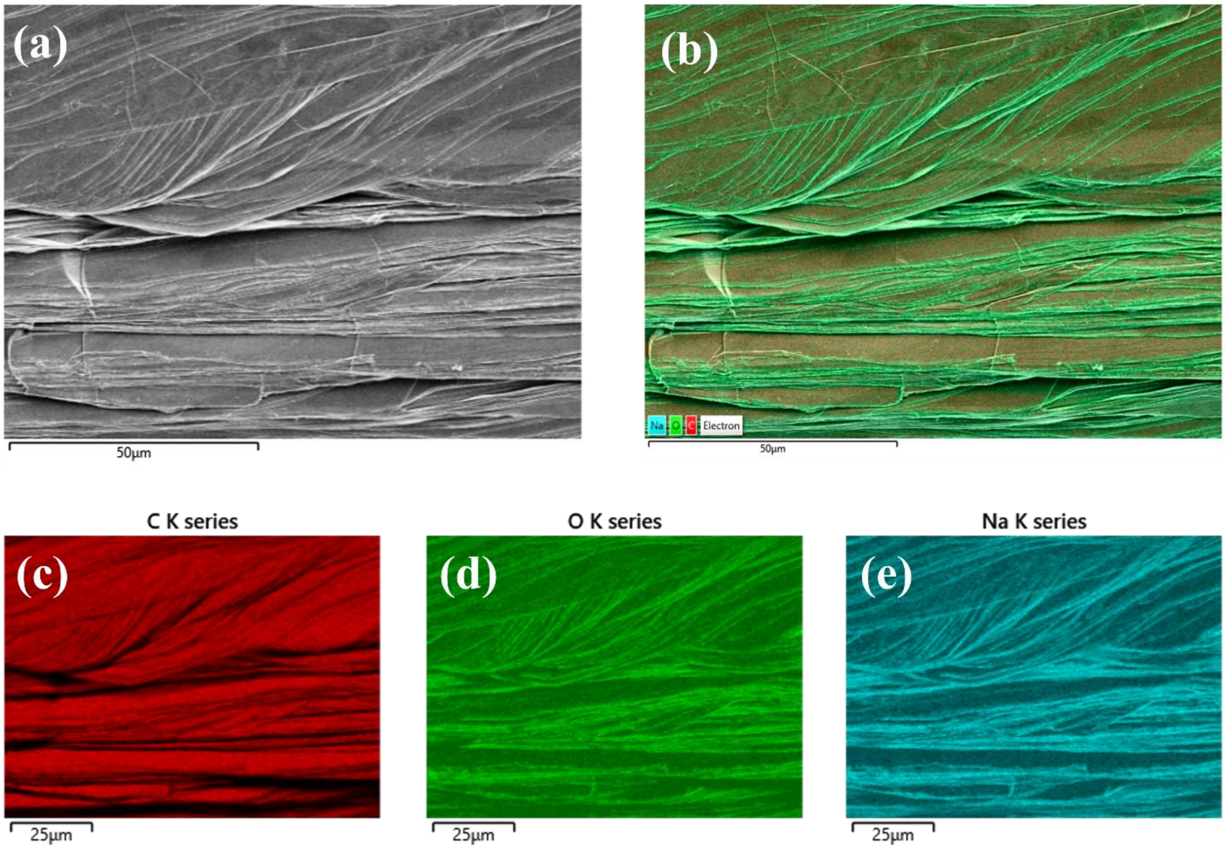

Fig. SⅢ-10. EDX elemental mapping of the cross-section (inner part) of electrochemically Na intercalated HOPG (Electrochemical test cell (coin cell) was assembled in an argon-filled glove box (O_2 ≤ 0.1 ppm, H_2O ≤ 0.1 ppm), with HOPG as working electrode, Na metal as the reference electrode. 1 M $NaPF_6$ solution in a 1:1 vol/vol mixture of ethylene carbonate (EC) and dimethyl carbonate (DMC) was utilized as the electrolyte and Whatman® glass microfiber (Grade GF/D) was used as separator. The cell was discharged to 0 V from OCV with a constant current and then held at 0 V for three weeks by using Arbin system (BT 2000). Then the cell was disassembled in an argon-filled glove box and the intercalated HOPG was washed using solvent DMC in order to remove the electrolyte residue. The electrochemically intercalated HOPG was cut into two pieces. The samples were mounted to a SEM holder in a glove box and transferred to SEM chamber in the protective environment using a vacuum transfer tool. The fresh cross-section of inner part was measured.).

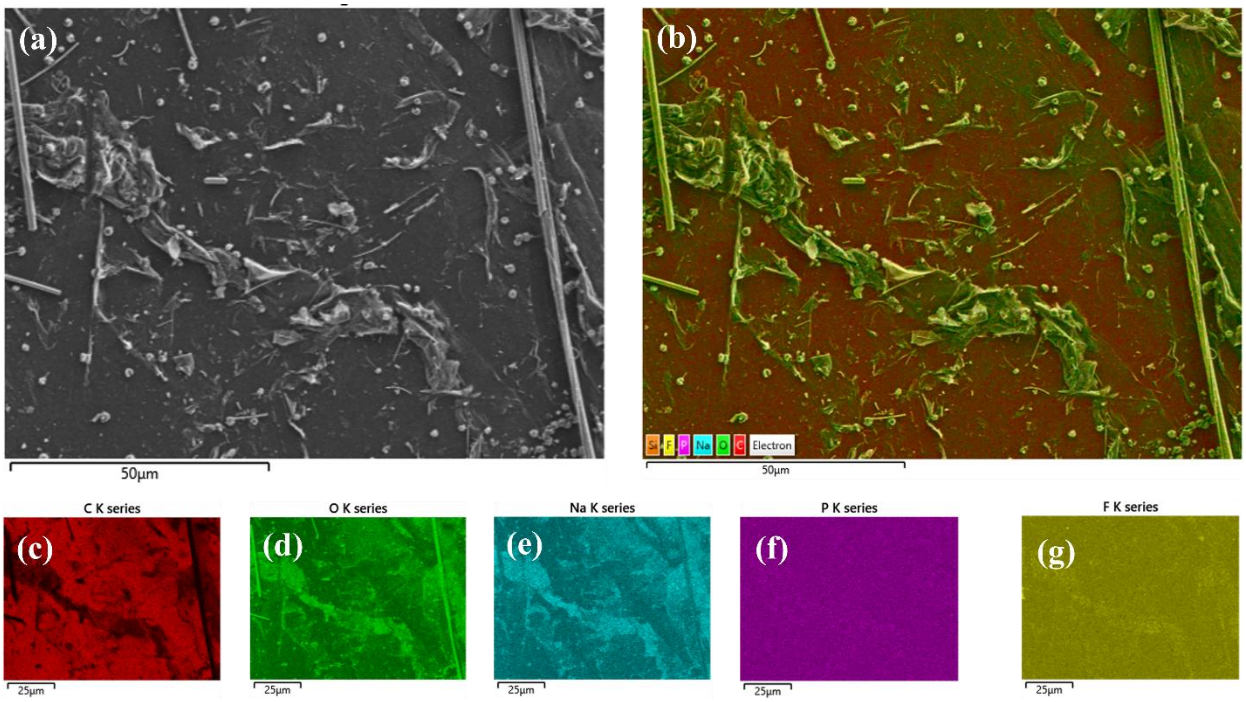

Fig. SⅢ-11. EDX elemental mapping of the top surface (basal plane) of electrochemically intercalated HOPG (for detailed sample preparation procedure, see Fig. SⅢ-10.).

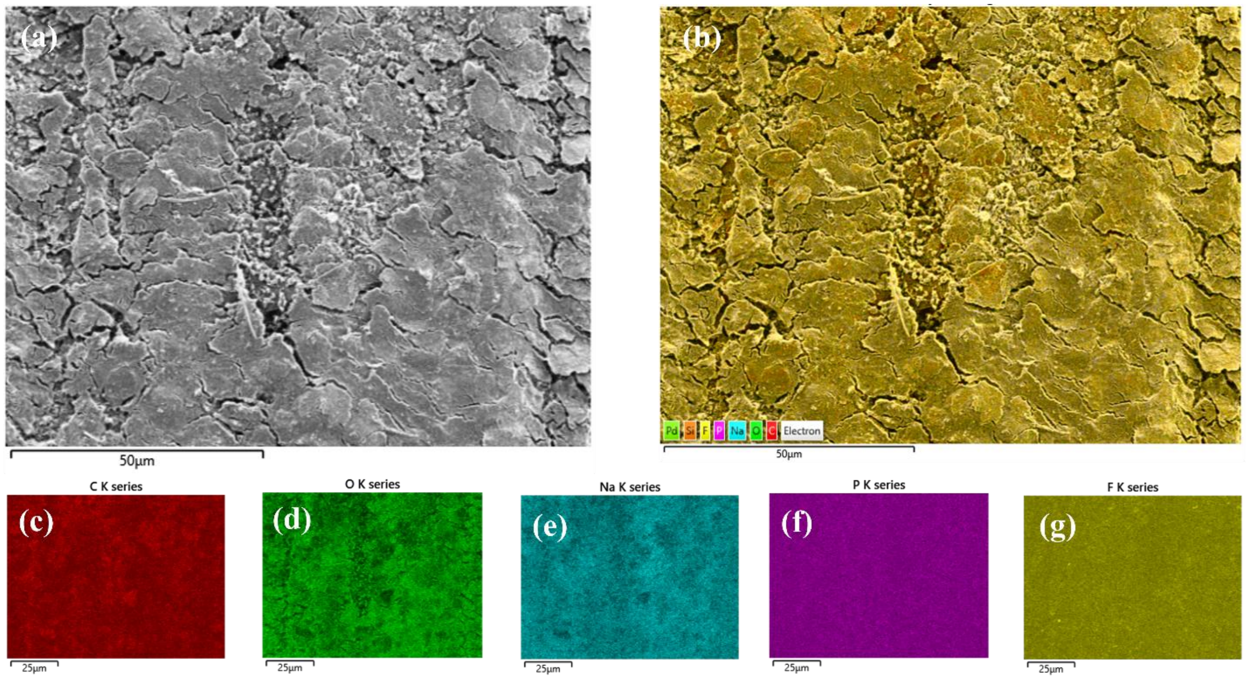

Fig. SⅢ-12. EDX elemental mapping of the cross-section (outer side-surface) of electrochemically intercalated HOPG (for detailed sample preparation procedure, see Fig. SⅢ-10.).

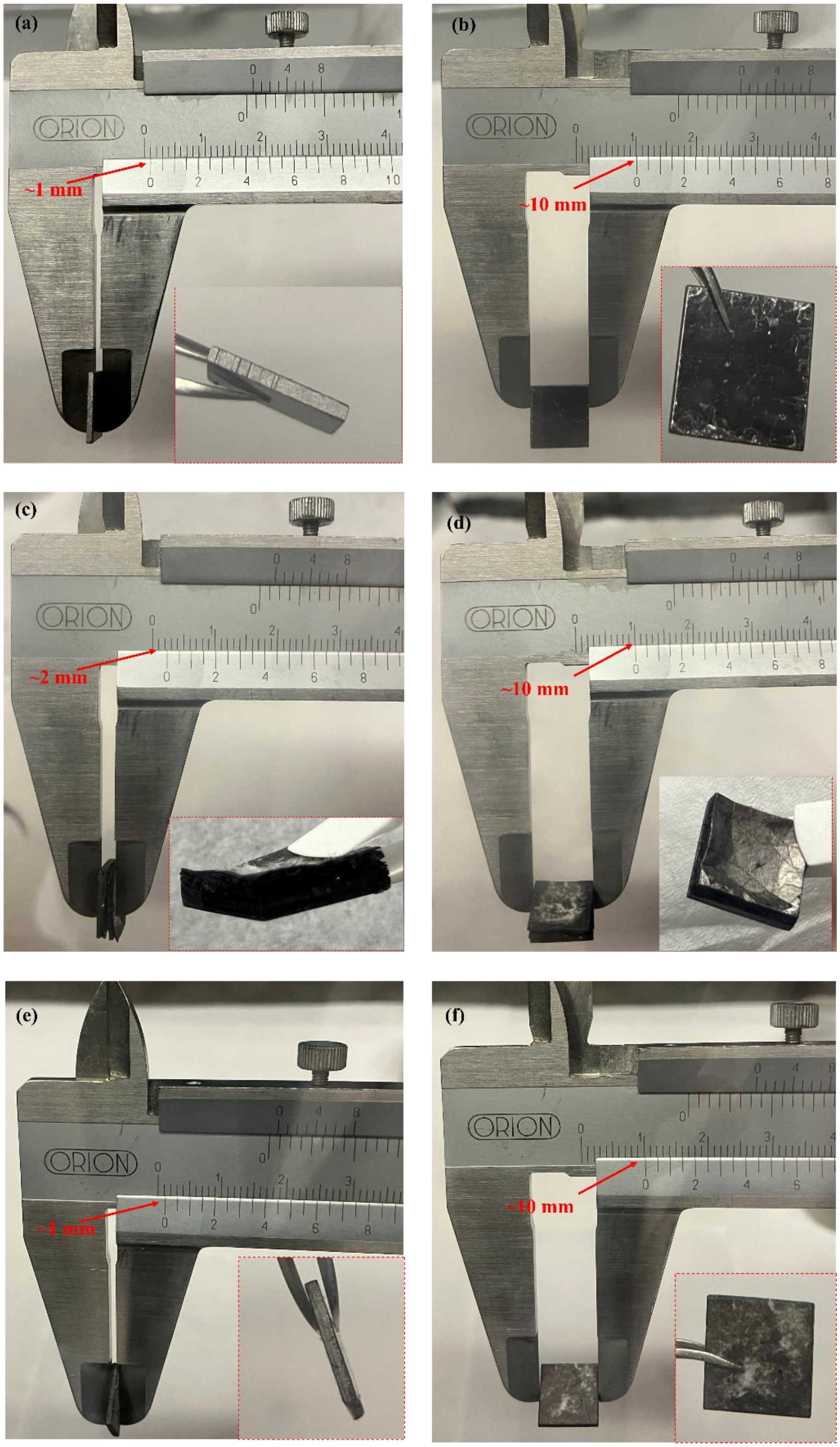

Fig. SⅢ-13. Photos of original HOPG (a, b), and treated HOPG (electrochemical intercalation of Na into HOPG at 0 V vs Na for 5 months at 25 °C) (c, d), as well as 2 nm TiO_2 coated HOPG at 0 V for 5 months at 25 °C (e, f). The comparison with the scale indicates the expansion.

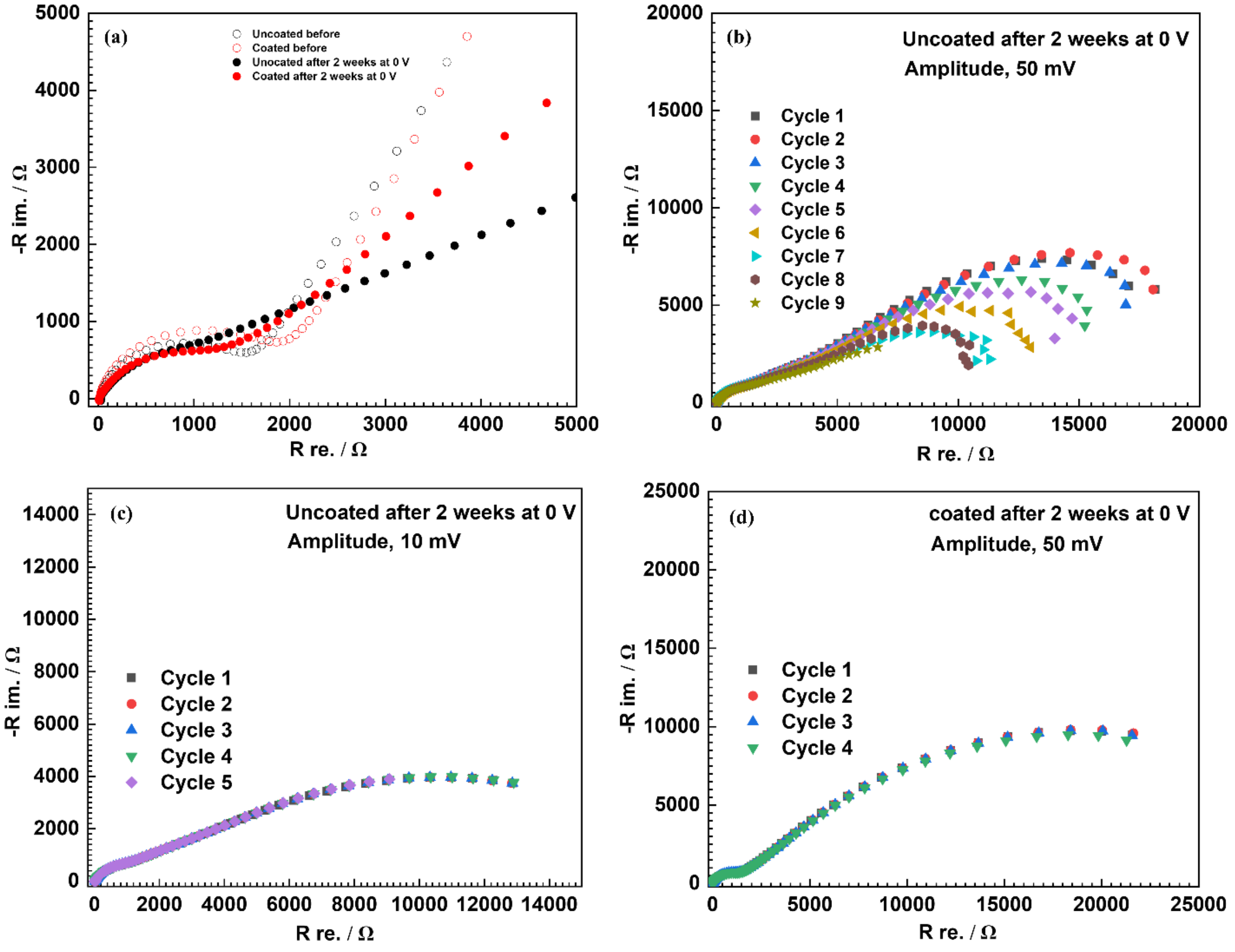

Fig. SⅢ-14. EIS spectra for electrochemical intercalation of Na at 0 V vs Na at 25 °C into uncoated- and 5 nm TiO_2 coated- HOPG. (a) Before and after Na intercalation applying 50, 10 mV (AC output amplitudes), respectively, (b) uncoated test sample and 50 mV, (c) uncoated sample and 10 mV, (d) coated sample and 50 mV.

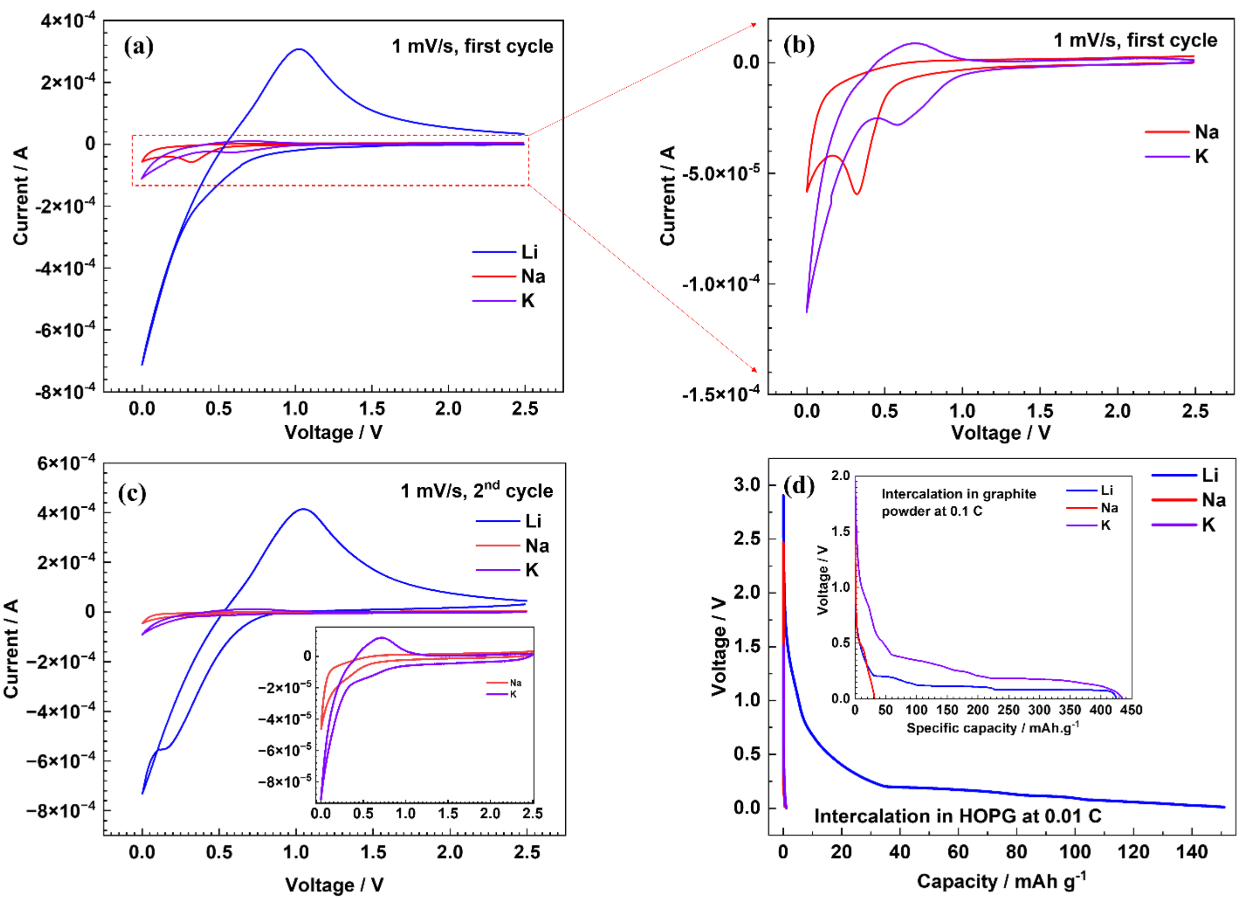

Fig. SⅢ-15. (a, b) CV for Li-, Na- and K- intercalation into HOPG at high sweep rate (1 mV/s) at 25 °C. The high voltage peaks around 0.4 V and 0.51 V for Na and K, respectively, appearing in the first cycle are ascribed to SEI formation; they disappear in the subsequent cycles as shown in (c), and the inset in (c) is zooming into the plots of Na and K. (d) Discharge curves for Li-, Na- and K- intercalation into HOPG at a current density of 0.01 C and polycrystalline graphite powder a current density of 0.1 C (inset) at 25 °C, respectively, suggesting available intercalation capacities of Li and K to be comparable while kinetics of K intercalation in HOPG is poor (Li: 1 C=372 mA/g, Na and K: 1 C=279 mA/g, voltage cutoffs: 10 mV for graphite powder and 0 mV for HOPG, respectively. HOPG mass for Li-, Na- and K- intercalation is 4.90, 4.82 and 7.57 mg, respectively.).

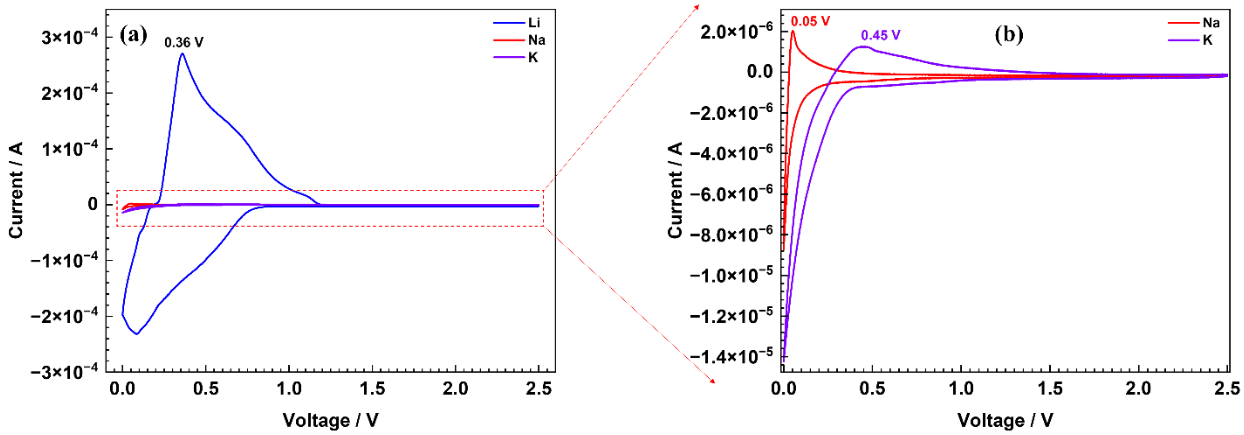

Fig. SⅢ-16. (a, b) CV at a sweep rate of 20 μV/s for Li-, Na- and K- HOPG at 25 °C. The peak current potentials of de-intercalation are 0.36 V, 0.05 V, and 0. 45 V for Li-, Na- and K- HOPG, respectively. The true OCV values should be in between de-intercalation and intercalation (close to 0 V) potentials.

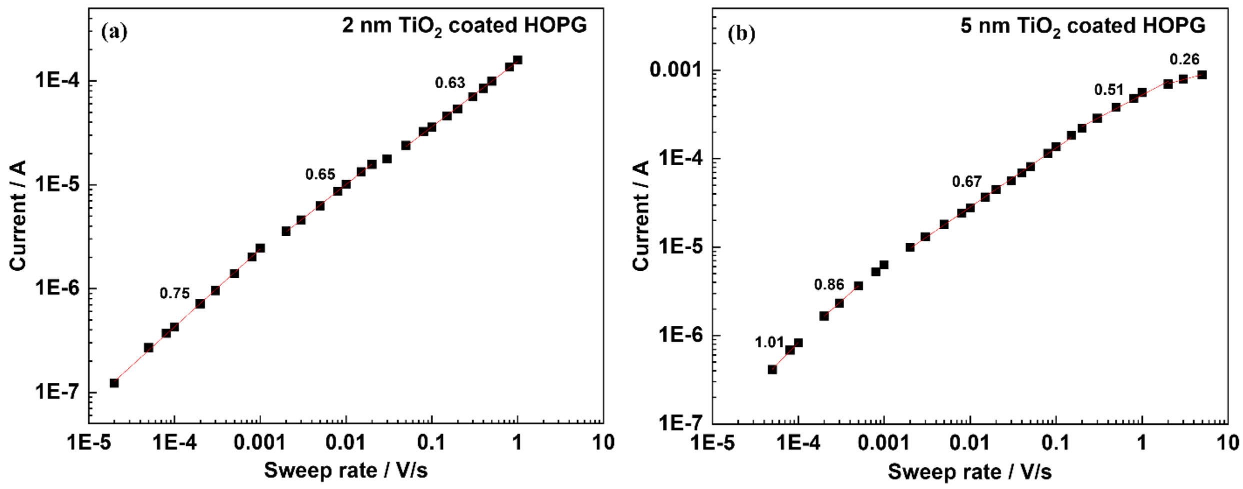

Fig. SⅢ-17. Peak current at various sweep rates ν of CV measurements for the 2 nm- and 5 nm-TiO_2 coated HOPG.

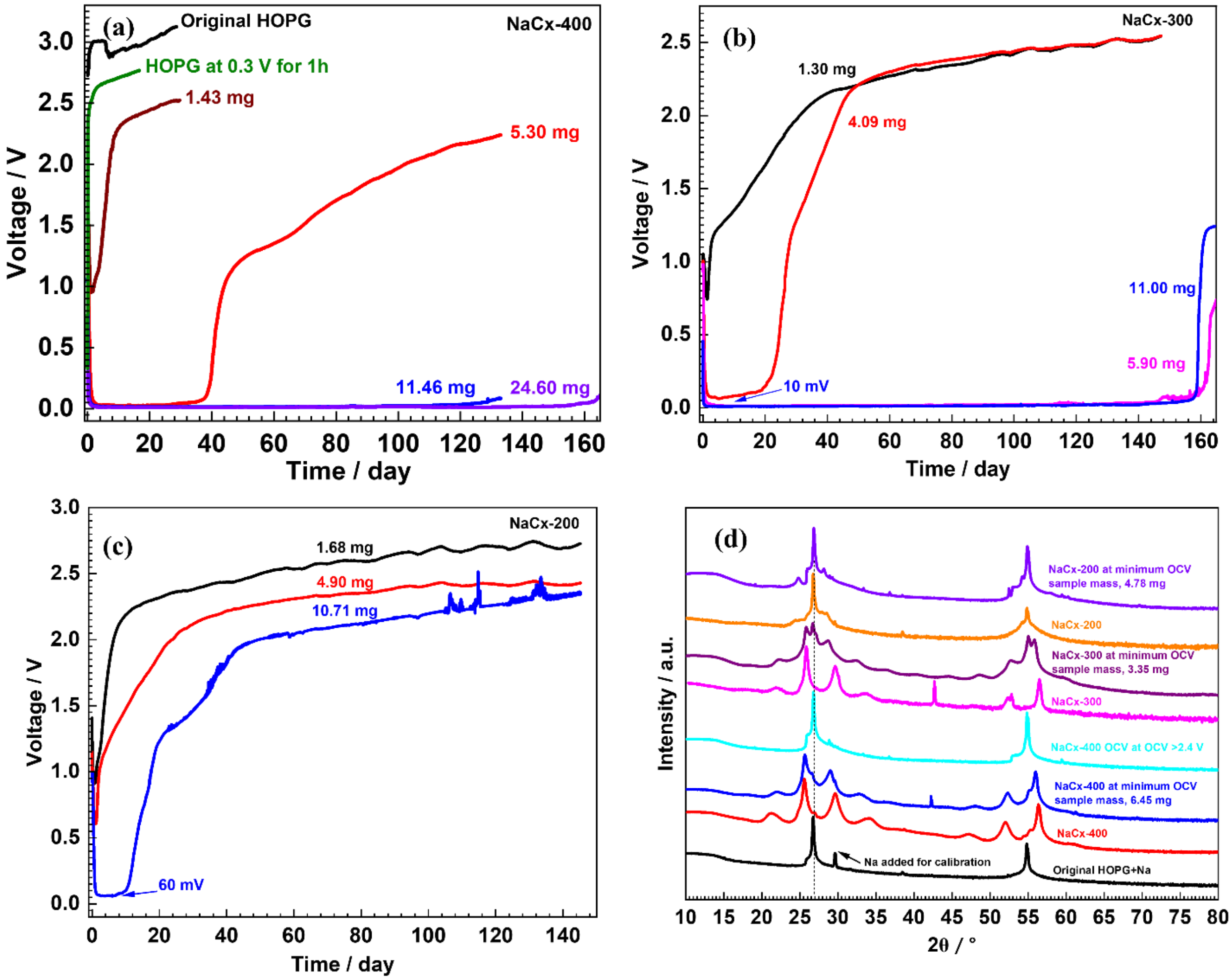

Fig. SⅢ-18. (a, b, c) Open circuit voltages (OCV) for chemical intercalation of Na into HOPG at 400 (NaC_x-400), 300 (NaC_x-300), and 200 °C (NaC_x-200), respectively. (d) XRD patterns for samples held at various states (minimum OCV denotes plateau range, e.g. 10 mV for NaC_x-300). Noting that both OCV and XRD measurements were carried out at 25 °C.

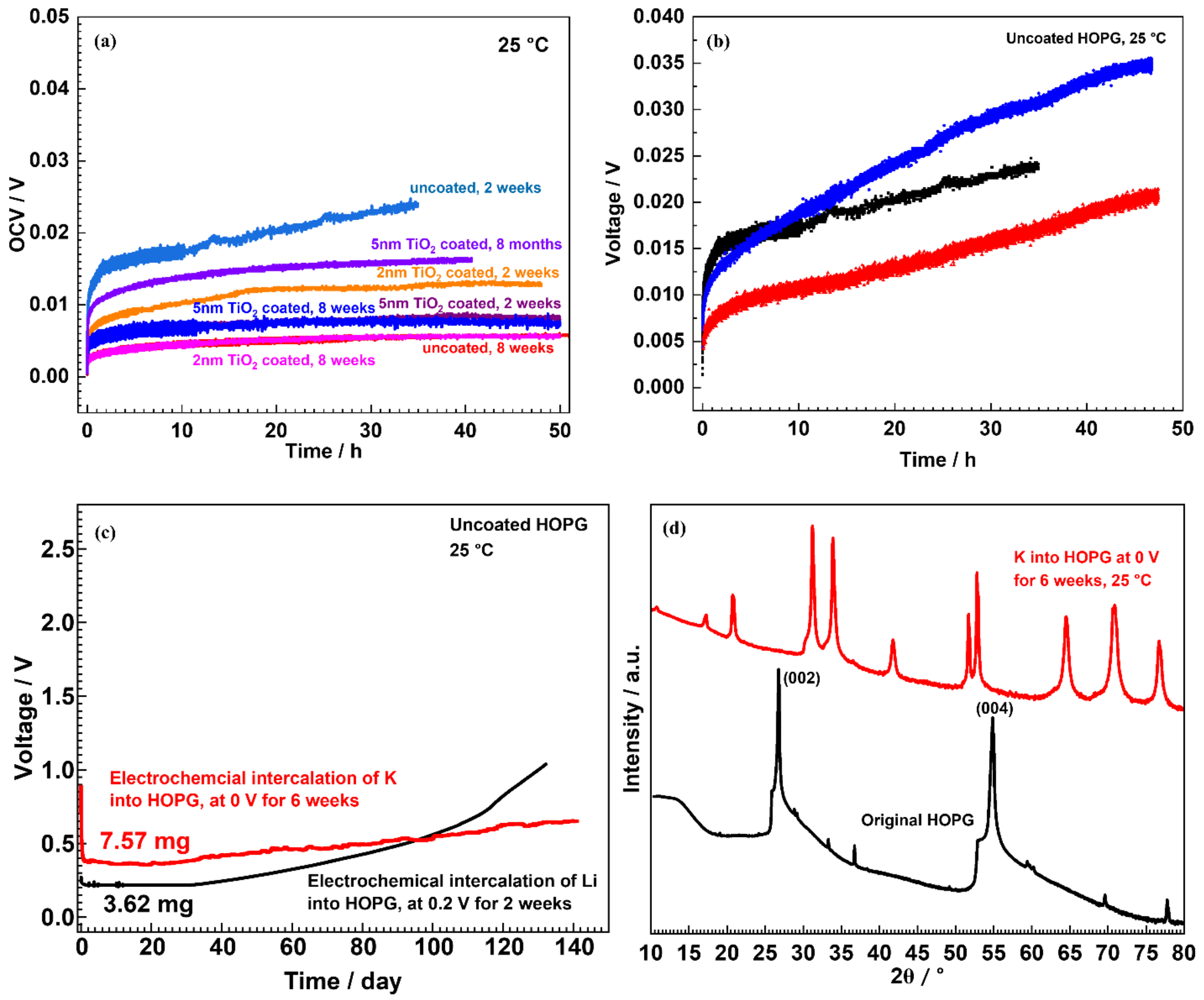

Fig. SⅢ-19. Open circuit voltages (OCV) for samples synthesized by electrochemical intercalation of Na (a, b) into uncoated and coated HOPG, (c) Li and K into HOPG, at 0 V at 25 °C. (d) XRD patterns corresponding to K intercalated HOPG of (c). The XRD pattern of Li intercalated HOPG is shown in Fig. SⅢ-20 below.

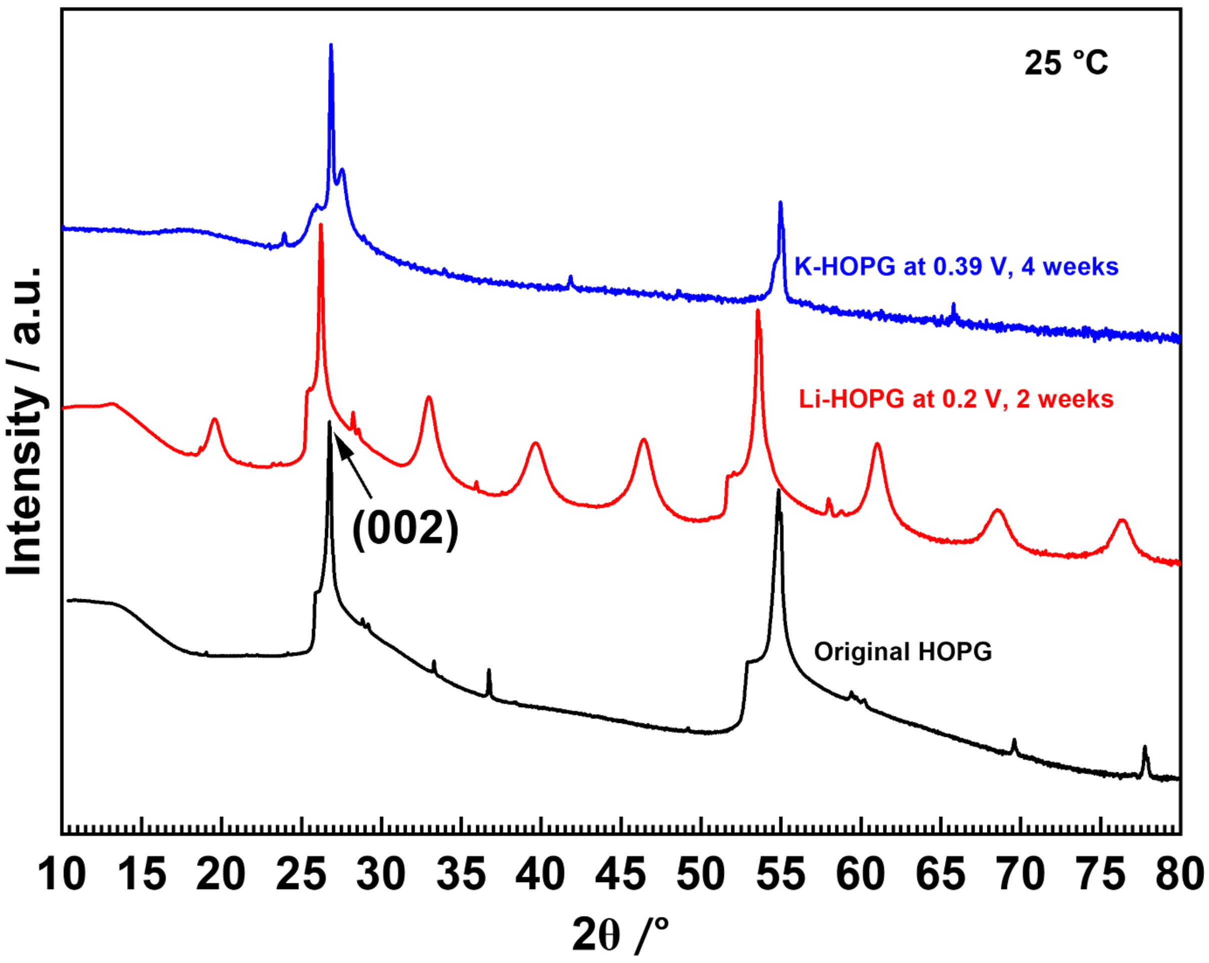

Fig. SⅢ-20. XRD patterns for electrochemical intercalation of Li and K into HOPG at 0.2 V and 0.39 V at 25 °C, respectively (note that (002) plane of graphite disappears after 2 weeks in Li case while not in K case, indicating K intercalation kinetics is poorer than Li intercalation).

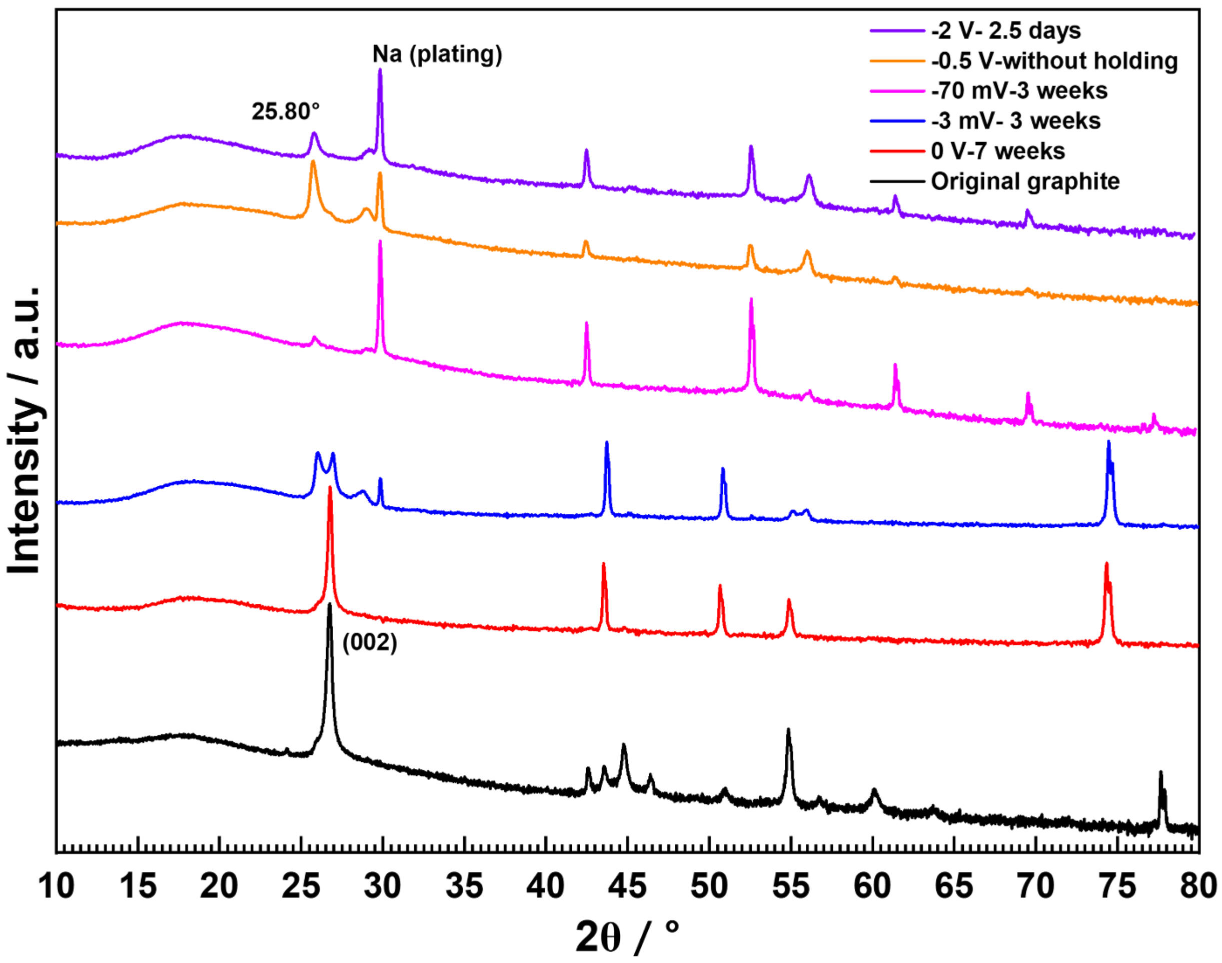

Fig. SⅢ-21. XRD patterns for electrochemical intercalation of Na into polycrystalline graphite powder at negative voltages at 25 °C (the intercalation was conducted by discharging the cell to 0 V vs Na with a constant current, followed by potential sweep control of -0.05 mV/s to certain voltages, then holding at the given voltages, e.g. -0.50 V). Note that both Na plating and Na intercalation signals are characterized for negative voltages applied.

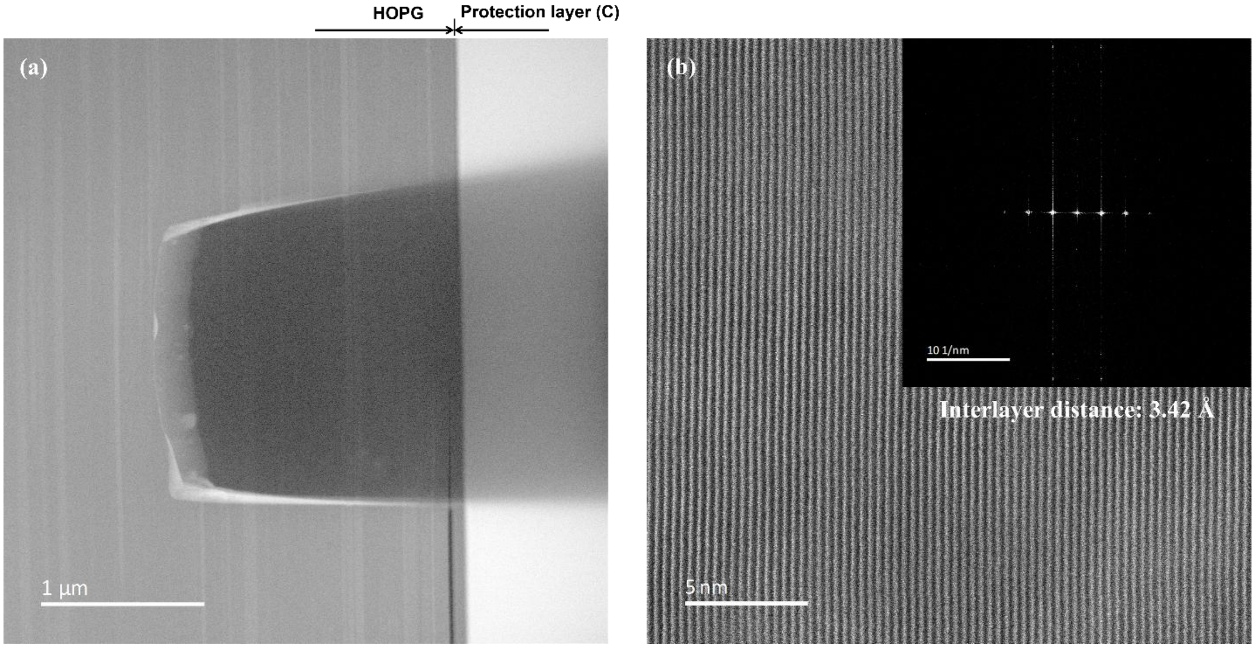

Fig. SⅢ-22. Original HOPG. (a) Low-magnification TEM image, (b) HAADF-STEM image (inset, Fast Fourier transform image).

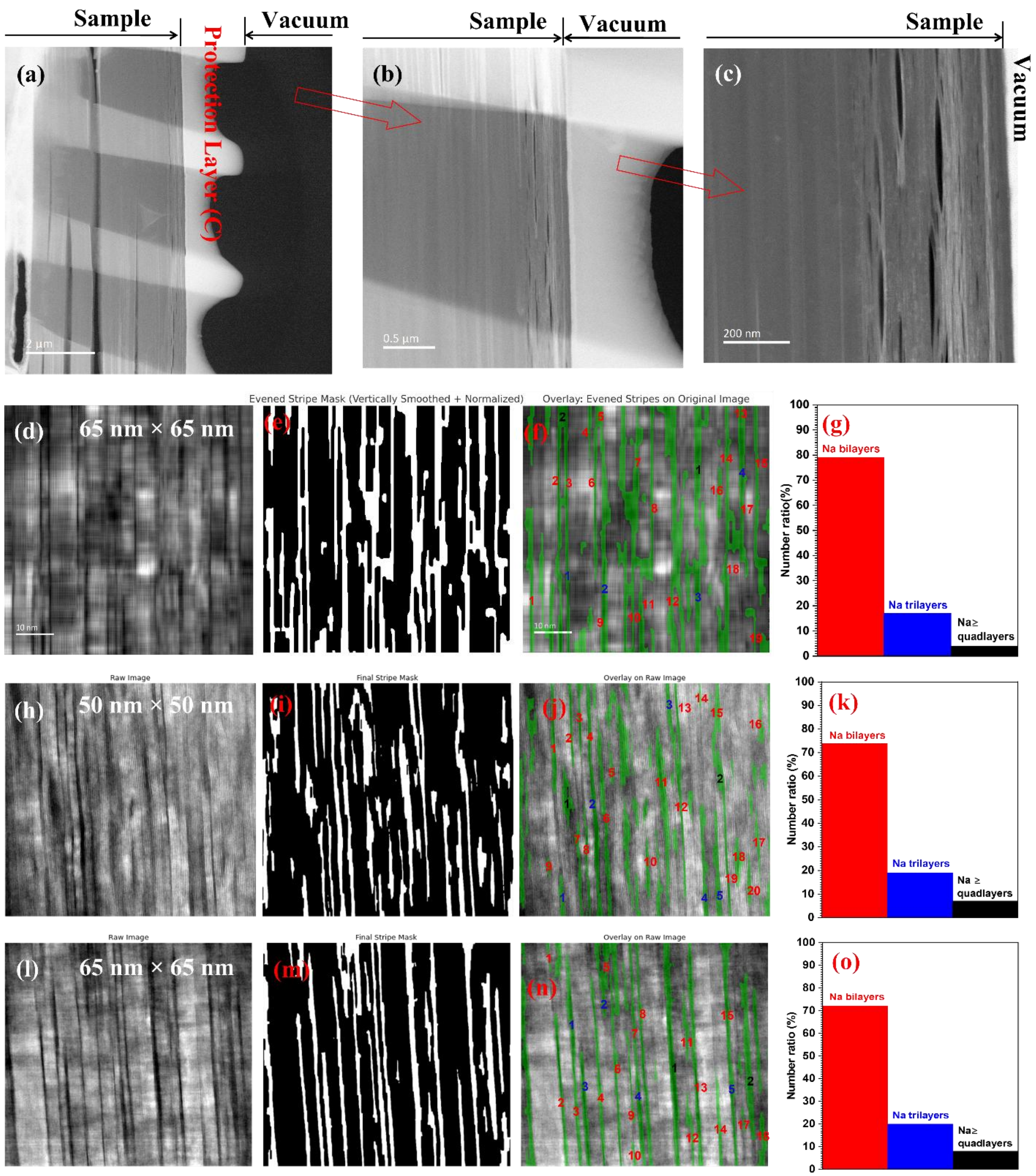

Fig. SⅢ-23. Na intercalated into HOPG compounds chemically synthesized at 400 °C with a waiting time of 2 weeks. (a-c) Low-magnification TEM image, (d, h, l) ADF-STEM images for representative regions, (e-g, i-k, m-o) statistics of number of Na bilayers, trilayers and ≥ quadlayers (note in images (f, j, n), red, blue and black numbers denote Na bilayers, trilayers and ≥ quadlayers counted, respectively).

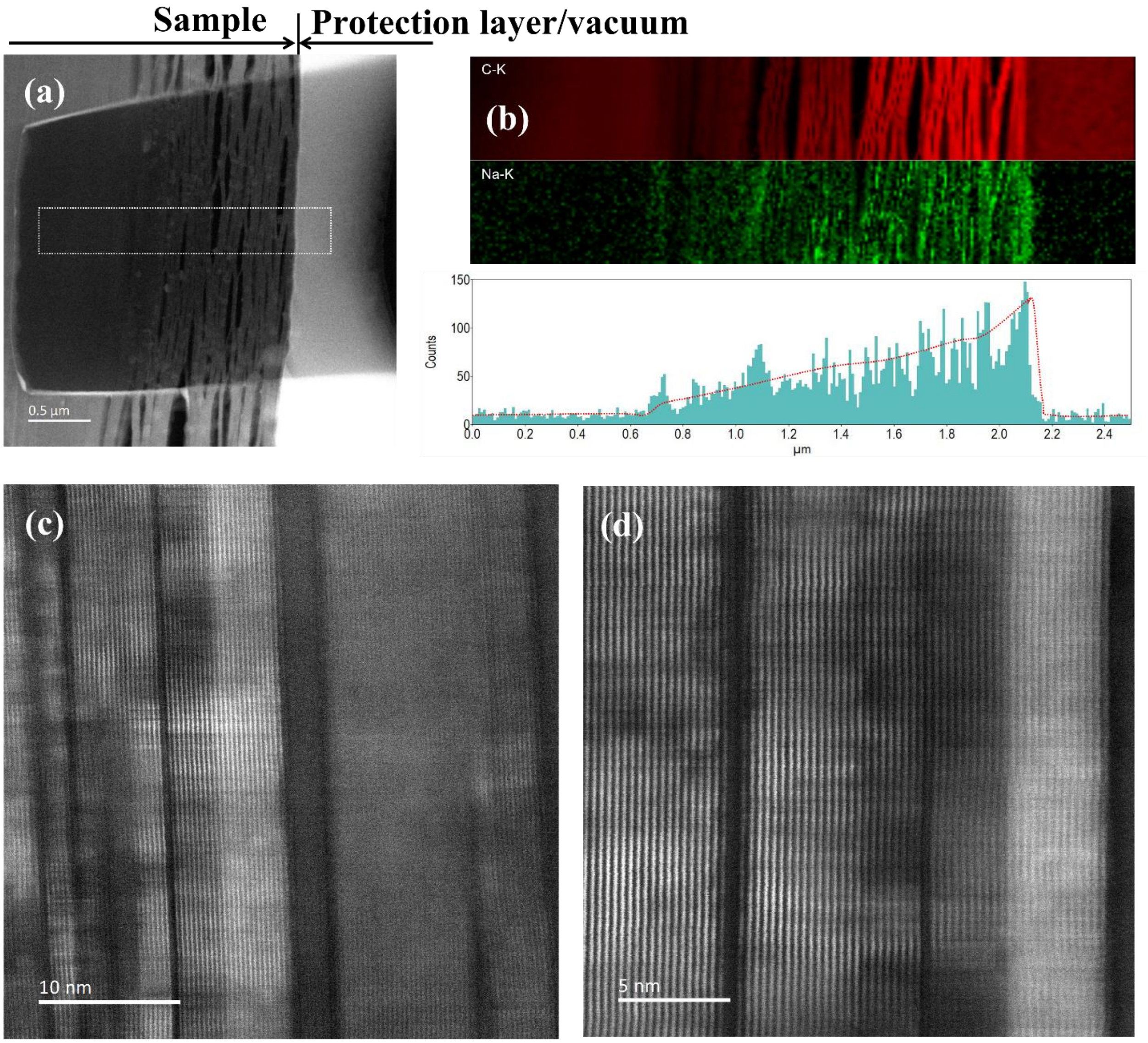

Fig. SⅢ-24. Na intercalated into HOPG compounds chemically synthesized at 25 °C with a waiting time of 10 months. (a) Low-magnification TEM image, (b) EELS mapping, (c, d) HAADF-STEM images.

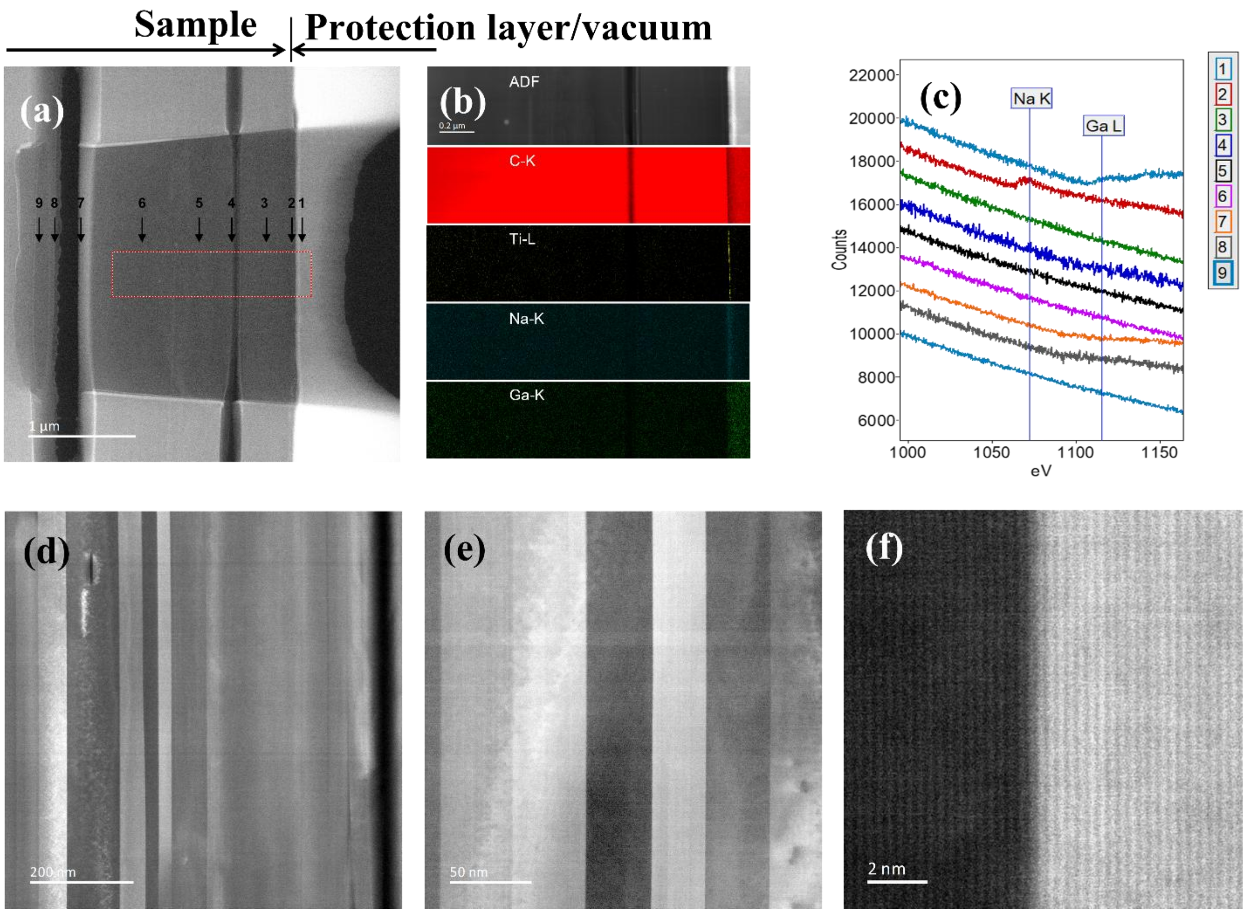

Fig. SⅢ-25. Na intercalated into 5 nm TiO_2 coated-HOPG compounds electrochemically synthesized at 25 °C, 0 V vs Na with a waiting time of 2 weeks. (a) Low-magnification TEM image, (b) EEELS mapping, (c) EELS spectra, (d, e, f) ADF-STEM images (note the bright and dark stripes arise from variations in the stacking configuration between layers, not intercalation signal. There is no confirmed Na intercalation signal based on EELS or distance of carbon-carbon interlayer which is attributed to extremely low Na concentration, but Na intercalation can be concluded by combing the cracks in image (a) and XRD data (Fig. SⅢ-4(a)), titration analysis (Table SⅡ-1) as well. Thus, it can be concluded that weakly Na intercalated in random distribution).

Part SⅣ XRD Simulations and Analyses

XRD pattern of the synthesized samples were recorded in Bragg Brentano geometry under inert atmosphere in order to avoid sample damage and degradation. The inert atmosphere sample holder did not allow for sample rotation. Hence, all XRD data suffered from preferred orientation. Moreover, it was not possible to adjust the z-position of the sample holder to the height of the film, this led to a sample height error, which artificially introduced a shift of the peak position. This can be clearly observed by the mismatch of the Na 011 peak in the XRD patterns of pure Na metal and Na metal admixed with original HOPG (Fig. SⅣ-1). Hence fully weighted Rietveld refinement cannot be performed on these samples and we resorted to a qualitative comparison with simulated XRD patterns. For the XRD simulations, we have to make assumptions: The intercalation of Na in-between graphite layers, leads to an increase in interlayer distance. However, there are no reliable data on this increase in interlayer distance available in literature. Hence, we use 3.80 Å, as observed by TEM measurement, as the interlayer distance of Na intercalated HOPG (see Fig. 3(c), Main Text. However, TEM data indicate the distances are not constant, varying even within the same Na bilayers from 3.60 Å to 4.02 Å. Here it is qualitative analysis/simulation of XRD, thus we assume 3.80 Å for simplicity). In addition, we assume that Na is situated above and below the centers of the $C_{6/3}$ rings and therefore, Na intercalation alters the ABAB stacking order of hexagonal graphite locally to an eclipsed AAAA stacking.

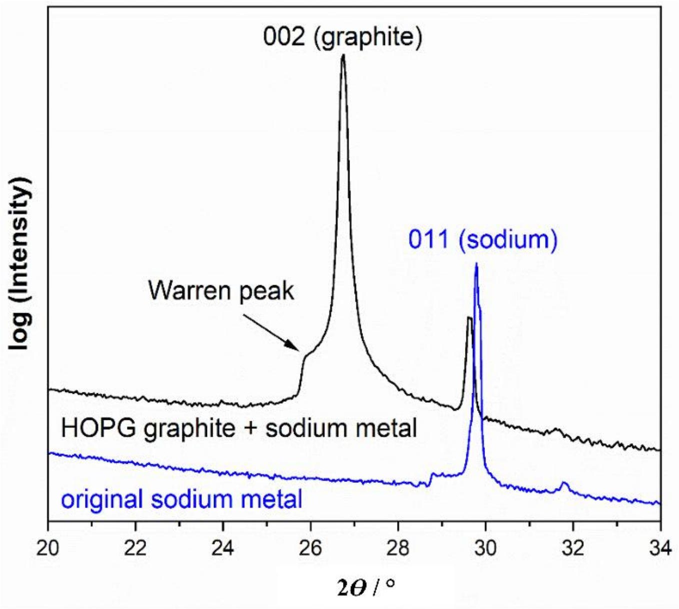

Fig. SⅣ-1. Comparison of the XRD pattern of pure Na metal and Na metal admixed with HOPG including selected reflection indices. The small, symmetry forbidden, trigonally shaped reflection in HOPG, denoted as "Warren peak",[9] indicates the presence of stacking disorder in HOPG.[10]

In the first series of simulations, it refers to random intercalation, i.e. no staging of ordered or disordered Na layers in-between graphite sheets, applying an intercalation probability of 25 %. Both scenarios lead to a considerable downshift (to lower diffraction angle) and broadening of the 002 reflection from 26.5 ° (2θ) to 25.3 ° (2θ) (Fig. SⅣ-2(a)). It should be noted, that the downshift of the 002 reflection depends on the increased interlayer distance of Na intercalated layer and on the intercalation probability. A higher intercalation probability and a larger increase in interlayer distance, would lead to more pronounced downshift of this peak. In case of ordered Na layers, an additional, triangular Warren-type peak can be observed at around 20.8 ° (2θ). In the second series of simulations, we tested the intercalation of randomized Na monolayers and bilayers (= two subsequent intercalation events, i.e. double Na) combined with three and four non-intercalated graphite layers. This microstructural motif is often referred to a staging.[11] Staging commonly implies that Na layers are filled first, before additional Na layers are intercalated in-between graphite layers. The occupation of Na within the intercalated layers, primarily effects the peak intensity, which is corrupted by pronounced preferred orientation. Hence, we will rather use the crystallographic term "Reichweite", describing the range of a fault, i.e. a Na intercalation event in the stacking of graphite, as we cannot make any quantitative statements on the population of the Na layers. Both the intercalation of Na monolayers and bilayers combined with a Reichweite of three or four, leads to a downshift of the 002 reflection but not to a considerable broadening of the peak (Fig. SⅣ-2(b)). In addition, satellite peaks appear both at lower and higher diffraction angles, with the satellites situated at higher diffraction angles having a significantly higher integral peak intensity than the ones at lower angles. The larger the Reichweite of the intercalation event, the lower are the satellites split apart from the 002 diffraction line. An intercalation of Na monolayers, also leads to less intense satellites than the intercalation of Na bilayers. In the next series of simulations, we compare the intercalation of an ordered vs. a randomized Na layers with a Reichweite of three layers (Fig. SⅣ-2(c)). Both simulated patterns of faulted intercalated-graphite are indistinguishable. This is attributed to the stacking faulted nature of graphite (Fig. SⅣ-1), as the position of an ordered Na, *n*, is randomized with respect to the preceding Na layer *n*-4 and the subsequent Na layer *n*+4. As a consequence of the strongly averaged information content of a powder pattern, the ordered Na positions appear to be smeared out and therefore disordered. There are, however, slight differences in the peak shape and the resulting peak maxima, in particular for non 00*l* reflections, but due to the pronounced preferred orientation of the samples, we cannot analyze the data in this depth. As a consequence, we cannot draw any conclusions of the atom arrangement of the intercalated Na layers from the XRD data. In the last series of simulations, we softened the criteria for monolayer and bilayer intercalation and for the Reichweite. At first, we simulated the single

intercalation of Na applying a Reichweite of three (Fig. SⅣ-2(d), green line). As previously seen, this leads to a downshift of the position of the 002 reflection compared to faultless graphite (Fig. SⅣ-2(d), blue and green lines) and to the emergence of satellite peaks at higher and lower diffraction angles. In the next step, we allow for an intercalation of a Na bilayer with a probability of 10 % ($P_{double\ Na}$ = 0.1) and a Reichweite of three but with a 10 % probability for the existence of a 4th (P_{4th} = 0.1) and a 5 % probability of a 5th (P_{5th} = 0.05) non-intercalated graphite layer within the stack (Table SⅣ-1). This leads to a broadening of the 002 reflection, whereas the position of the peak maximum is hardly affected (Fig. SⅣ-2(d), green and magenta lines). The satellites, however are broadened to a higher degree and appear nor much broader than the 002 reflection. In addition, they are shifted more closer to the 002 peak. Finally, we simulated a diffraction pattern in which an intercalation of a Na layer occurs with a probability of 90 % ($P_{double\ Na}$ = 0.9) and a Reichweite of three but with a 10 % probability for the existence of a 4th (P_{4th} = 0.1) and a 5 % probability of a 5th (P_{5th} = 0.05) non-intercalated graphite layer within the stack. The position of the 002 reflection is now shifted towards significantly lower diffraction angles, as the mean interlayer distance is increased compared to the other simulated patterns (Fig. SⅣ-2(d), magenta and cyan lines). The satellite peaks appear less broadened but still broader than the 002 reflection. The downshift of the satellite at higher diffraction angles is considerably larger than the upshift of the satellite at lower angles.

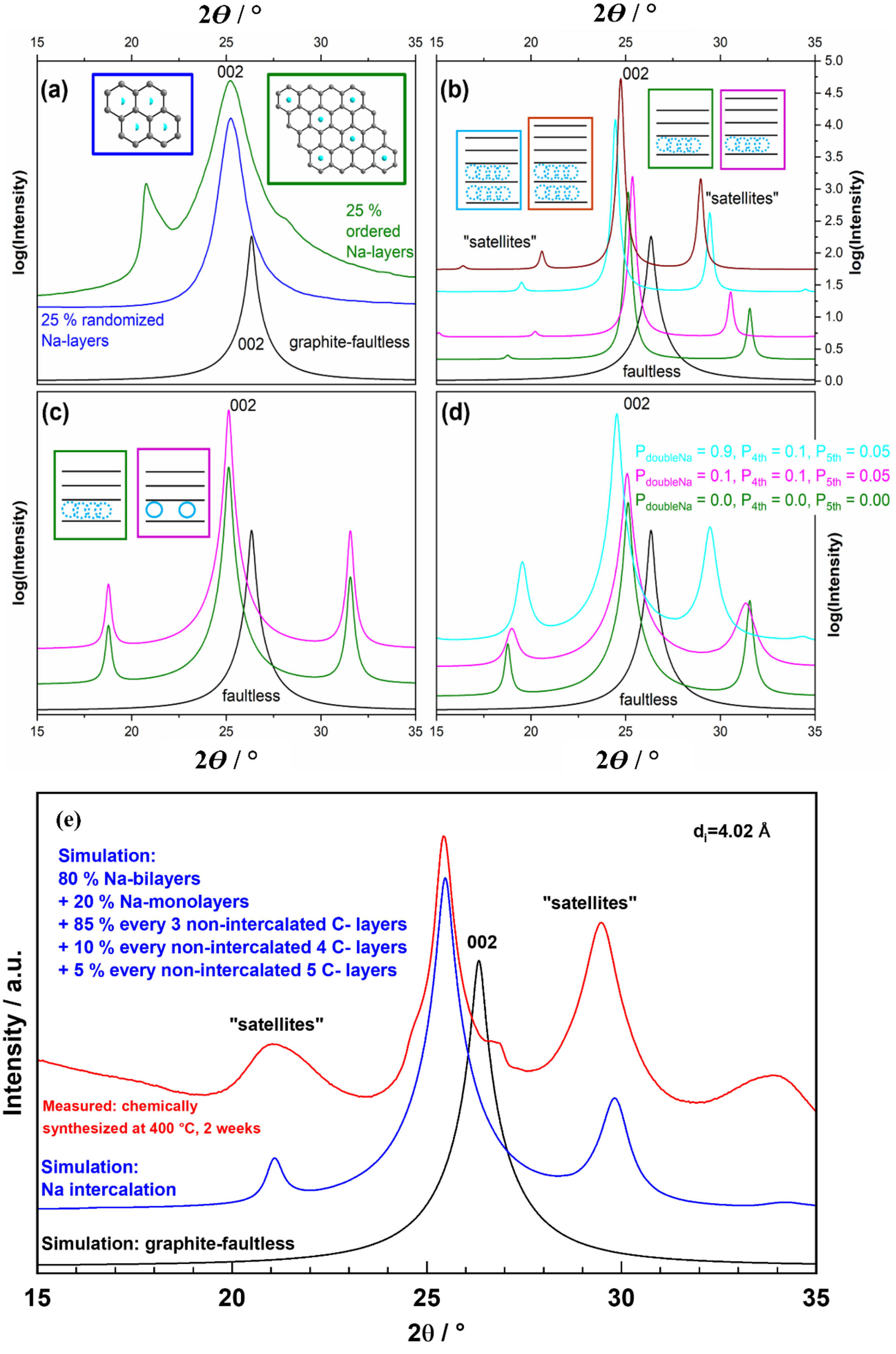
2θ / °
log(Intensity)
(a)
002
25 % ordered Na-layers
25 % randomized Na-layers
002
graphite-faultless
(b)
002
"satellites"
"satellites"
faultless
(c)
002
faultless
(d)
002
$P_{doubleNa}$ = 0.9, P_{4th} = 0.1, P_{5th} = 0.05
$P_{doubleNa}$ = 0.1, P_{4th} = 0.1, P_{5th} = 0.05
$P_{doubleNa}$ = 0.0, P_{4th} = 0.0, P_{5th} = 0.00
faultless
(e)
d_i=4.02 Å
Simulation:
80 % Na-bilayers
+ 20 % Na-monolayers
+ 85 % every 3 non-intercalated C- layers
+ 10 % every non-intercalated 4 C- layers
+ 5 % every non-intercalated 5 C- layers
"satellites"
002
"satellites"
Measured: chemically synthesized at 400 °C, 2 weeks
Simulation: Na intercalation
Simulation: graphite-faultless
Intensity / a.u.
2θ / °

Fig. SⅣ-2. Simulations of Na intercalated hexagonal graphite using different faulting scenarios: (a) random intercalation of ordered vs. disordered Na layer (no staging), (b) intercalation of Na monolayers and bilayers followed by three and four non-intercalated graphite layers, (c) intercalation of Na monolayers, followed by three non-intercalated graphite layers, (d) intercalation of Na bilayers, with randomization of Reichweite of the intercalation (Table SⅣ-1), (e) chemical intercalation of Na in HOPG at 400 °C for 2 weeks by assuming Na intercalated interlayer distance of carbon-carbon layers d_i=4.02 Å.

The comparison of the measured XRD patterns of the starting materials, HOPG with Na-metal and Na-intercalated HOPG obtained by chemical intercalation at 400 °C after two weeks (Fig. SⅣ-3(a), blue and green lines) shows, that the 002 reflection is significantly downshifted by almost 3 ° (2θ) and slightly broadened. In addition, satellite peaks appear at lower and higher diffraction angles, which are significantly broader than 002 diffraction line. The overall shift on the 002 peak position is an indicator for a successful intercalation of Na in-between the graphite layer, which corresponds to an increase of the mean interlayer distance. The satellite peaks indicate a Reichweite of the intercalation event (Fig. SⅣ-2(b, c, d)) and broadening of both the 002 diffraction and of the satellites could point to the co-existence of both Na monolayers, bilayers and perhaps even multi-layer Na intercalation. The measured peak positions do not exactly match the reflection positions in the simulated pattern, which corresponds to an intercalation of a Na layer occurs with a probability of 90 % ($P_{double\ Na}$ = 0.9) and a Reichweite of three but with a 10 % probability for the existence of a 4th (P_{4th} = 0.1) and a 5 % probability of a 5th (P_{5th} = 0.05) non-intercalated graphite layer within the stack. This can be assigned to multiple factors: differences in the overall intercalation probability, further modulation of the interlayer distance by partial occupation of the Na layers or a different or broader distribution of the Reichweite of the defect. Given to the limited data quality (preferred orientation, poor particle statistics, sample height error), the multi-dimensionality of the anticipated microstructural model and the fact that a modulation of the Reichweite of defects turns the dimension of the transition probability matrix into a parameter, which needs to be optimized,[12] we refrain from any quantitative statements. The chemical intercalation of Na at room temperature (25 °C) seems to lead to an incomplete conversion, as neither the position nor the broadening of the 002 diffraction line changes (Fig. SⅣ-3(b)). After 10 months (magenta line), a small and broad satellite appears at higher diffraction angles. Eventually, after one year (cyan line), another broad reflection emerges at lower diffraction angle. Considering the peak position, it could be both a satellite or another 00l' diffraction line. The latter would indicate a two-phase character of the sample with a non-intercalated phase co-existing with a Na-intercalated HOPG phase, in which defects are associated with a considerable

Reichweite, most likely exceeding quadlayers. Another explanation would be a low intercalation probability, which is connected with a high Reichweite, as an increase in the Reichweite, leads to smaller splitting of the peak position of the 002 diffraction line and its satellites (Fig. SⅣ-2(b)). The electrochemical intercalation of Na in HOPG seemingly leads to a splitting of the 002 diffraction line (Fig. SⅣ-3(c)) into a larger peak at higher and a smaller reflection at lower diffraction line. This however, would mean that the electrochemical intercalation of Na leads both to an increase and decrease of the interlayer distance compared to original HOPG. The latter fact seems to be very unlikely. Hence, we interpret the XRD pattern as a low Na intercalation, with large Reichweite. The satellite at lower diffraction angles might be broadened to a degree that it merges with the background. It should be noted, that this satellite is also considerably more broadened in the chemically intercalated samples (Fig. SⅣ-3(a, b)). When the electrochemical intercalation is extended from 2 weeks (Fig. SⅣ-3, green line) to 8 weeks (magenta line), the 00l reflection slightly shifts towards lower diffraction angles and the peak becomes broader, the satellite peak also broadens. This and the fact, that the shape of the 00l reflection becomes slightly more trigonal shaped leads to the conclusion, that the intercalation process proceeds over time, but the Na layers are distributed more randomly within the stack (Fig. SⅣ-2(a)). The electrochemical intercalation leads to different diffraction patterns, when HOPG is coated by 5 nm TiO_2 (Fig. SⅣ-3(d)). Here additional satellite peaks 011 appearing at higher diffraction angles, are reflections attributed to Na metal intentionally put for calibration. Overall, these patterns are comparable to the XRD data obtained from chemical intercalation at room temperature (Fig. SⅣ-3(b)). Hence, the diffraction effects could be explained by similar microstructural effects. As the satellite peak at, lower diffraction angles, which is most likely another 00*l*' line, we assign this to a two-phase character of the sample with a non-intercalated phase co-existing with a Na-intercalated HOPG phase, in which defects are associated with a considerable Reichweite, most likely exceeding quadlayers. The intercalation probability is considerably higher than for the chemically intercalated sample. Due to the broadening of the 00*l*' reflection and its triangular shape, we also conclude that the distribution of Na layers within the stack is partially randomized.

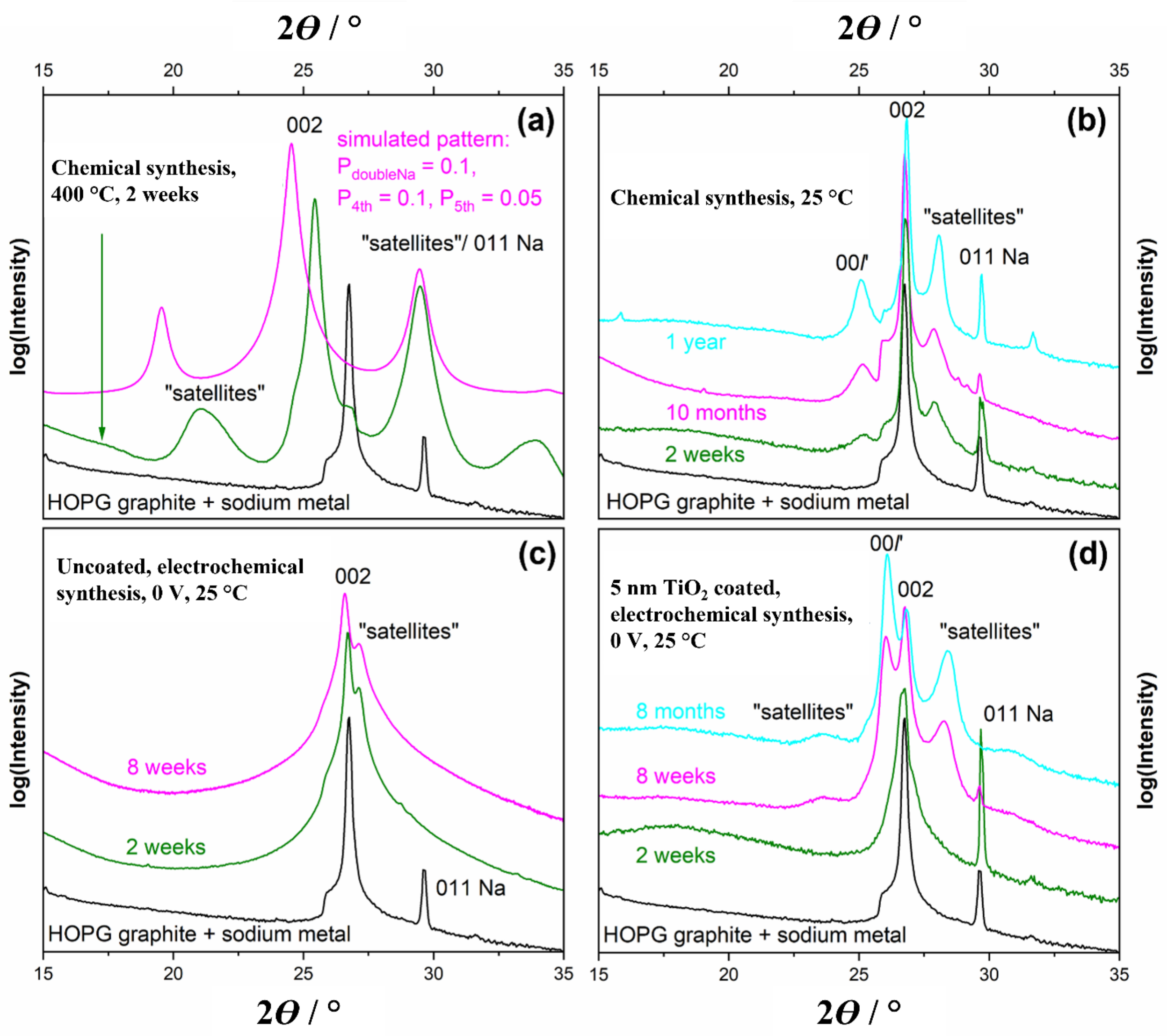

Fig. SⅣ-3. (a) Comparison of the measured XRD patterns of HOPG with Na-metal, the starting materials (blue line), Na-intercalated HOPG obtained after two weeks of chemical intercalation at 400 °C (green line) and the simulated pattern intercalation of Na bilayers, with randomization of Reichweite of the intercalation event (magenta line, Fig. SⅣ-2(d), cyan line), (b) comparison of the measured XRD patterns of HOPG with Na-metal, the starting materials (blue line) and Na-intercalated HOPG obtained after two weeks (green line), ten months (magenta line) and one year (cyan line) of chemical intercalation at 25 °C, (c) comparison of the measured XRD patterns of HOPG with Na-metal, the starting materials (blue line) and Na-intercalated HOPG obtained after 2 (green line) and 8 weeks (magenta line) of electrochemical synthesis at 0 V at 25 °C, (d) comparison of the measured XRD patterns of HOPG with Na-metal, the starting materials (blue line) and Na intercalated-5 nm TiO_2 coated HOPG after 2 (green line) and 8 weeks (magenta line) and 8 months (cyan line) of electrochemical synthesis at 0 V at 25 °C.

Table SⅣ-1. Transition probability matrix used for the faulting scenario of intercalation of Na bilayers (double layers), with randomization of Reichweite of the intercalation event (Fig. SⅣ-2(d))

to→ / from↓	Na+Graphite	Na+Graphite	Graphite	Graphite	Graphite	Graphite	Graphite	Graphite
Na+Graphite	0	$\mathbf{P_{double\ Na}}$	1-$\mathbf{P_{doubleNa}}$	0	0	0	0	0
Na+Graphite	0	0	1	0	0	0	0	0
Graphite	0	0	0	1	0	0	0	0
Graphite	0	0	0	0	1	0	0	0
Graphite	0	0	0	0	0	$\mathbf{P_{4th}}$	0	1-$\mathbf{P_{4th}}$
Graphite	0	0	0	0	0	0	$\mathbf{P_{5th}}$	1-$\mathbf{P_{5th}}$
Graphite	0	0	0	0	0	0	0	1

Part SV Entropy and Enthalpy Problems

1 Configurational entropy of intercalation planes

At a first glance the higher degree of aggregated layers when the temperature is reduced, might be explained by the loss of configurational entropy ($S_{\text{conf.}}$) of layer arrangement and then by mass action laws for (dis-)aggregation. (Whether we consider monolayer or bilayer is not relevant) However, $S_{\text{conf.}}$ is much too small in comparison to the aggregation energy. It suffices to study the site exchange of N_{I} monolayers within the ensemble of $N_{\text{I}} + N_{\text{C}}$ layers (Fig. SV-1).

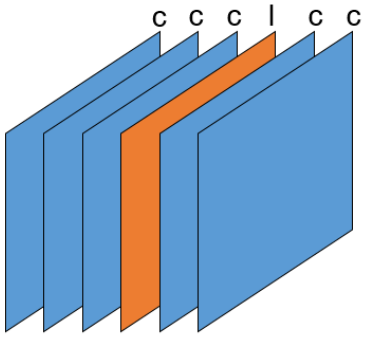

Fig. SV-1. Statistics of a random layer arrangement.

Let us assume a random distribution, then

$$S_{\text{conf.}} = k \ln \binom{N_{\text{I}} + N_{\text{c}}}{N_{\text{I}}}$$

and the chemical potential of a layer

$$\mu_{\text{I}} = \mu_{\text{I}}^{\circ} + kT \ln \left(\frac{N_{\text{I}}}{N_{\text{c}}} \right)$$

where k is Boltzmann constant, μ_{I}° standard chemical potential, and T temperature in kelvin.

For the equilibrium concentration we find

$$\frac{N_{\text{I,eq}}}{N_{\text{c}}} = \exp\left(-\frac{\Delta G_{\text{I}}^{\circ}}{RT}\right)$$

where $\Delta G_{\text{I}}^{\circ}$ is the standard free enthalpy of forming a single intercalated layer (I). The value refers to the entire plane and is massively larger than value for single atomic constituents (at). Moreover, the reference quantity N_{c} is vastly smaller than the reference value for single atomic constituents ($N_{\text{c}} \sim N_{\text{at}}^{1/3}$). In total

the equilibrium concentration is much smaller than unity, i.e. the configurational entropy of layer rearrangement is absolutely negligible (see page 119-120, ref.).[13]

2 Configurational entropy of Na distribution within a layer

Let us consider the configurational entropy of the rearrangement of atomic constituents (Na) within a given plane and again assume a random distribution (Fig. SⅤ-2).

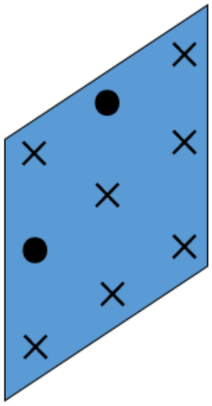

Fig. SⅤ-2. Statistics of random Na distribution for a given plane.

We can adopt the above equations when N_I is relabeled as N_Na, and N_c is now to be understood as the number of rings not "occupied" by Na in say the NaC_6 structure (the rings that are not occupied even in the fully intercalated state are not included). The entropy value is much higher than in the previous example as we refer to point defects (but less than in the 3D case to be considered below; $N_\text{total sites in plane} \sim N^2_\text{total planes} \sim N^{2/3}_\text{total sites in total volume}$). Specifically, we will estimate the free energy change when Na atoms of a fully occupied monolayer (fully occupied or vacant are defined relative to a NaC_6 structure) are redistributed over two neighboring layers such that the chemical bilayer is formed. The free energy change is at minimum, if approximately

$$\mathrm{const} + kT \ln \frac{x}{1-x} = 0$$

where $x = \frac{N_\mathrm{Na}}{N_\mathrm{c}+N_\mathrm{Na}}$ and const refers to the change of the standard potential on this transfer.
When ordering effects are negligible, const is rather small, and we find $x \simeq 0.5$. Like in the previous section mass actions can be formulated, whose solution gives complete preference to the state of lowest free standard enthalpy (here the aggregated state).

The other extreme would be a strongly ordered situation. Here now the first term is significant and variable, while the configurational term is small. Then according to the main text, the equilibrium x might be also 0.5.

Whether in the bilayers the above entropy argument holds for both layers separately or if double the sites are available, depends on the exchangeability between the layers which appears to be only relevant if there are imperfections that allow for perpendicular transport.

3 Entropy and enthalpy of charged particles in an ideal space charge layer

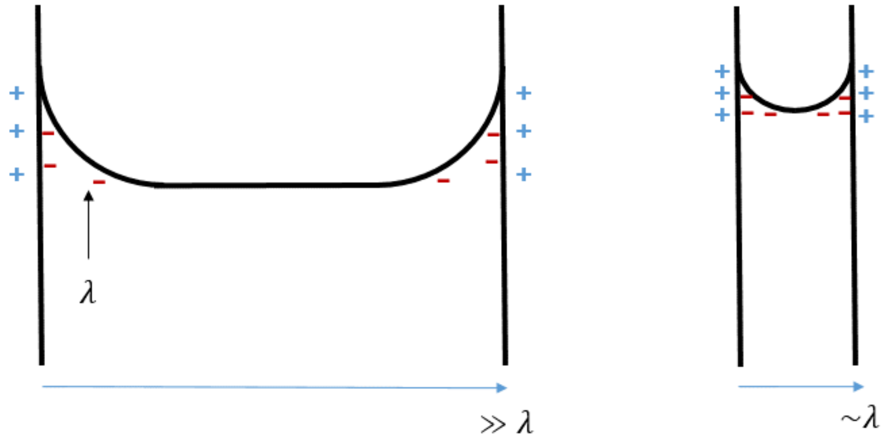

Fig. SⅤ-3 Sketch of two space charge layers.

Fig. SⅤ-3 gives the situation for a distance of two charged layers, which is far beyond the screening length λ (left) and on the order or less than λ (right), displayed for the case of fixed interfacial charges. On approach of very distant layers towards the situation shown at the left, certainly the energetic situation differs but also the configurational entropy for the distribution of the diffuse charges. In fact, various authors in particular Overbeek have shown that the latter contribution is dominant in the majority of cases.

In such a situation where electric fields occur, the decisive potential is the electrochemical potential (that unlike the chemical potential also includes the electric potential ϕ)

$$\widetilde{\mu} = \mu^{\circ} + ze\phi + \mu_{\mathrm{conf.}}$$

In the space charge free enthalpy (G_{scl}), it is useful to separate the space charge enthalpy (H_{scl}) from the configurational entropy of space charge distribution, i.e. ΔS_{scl}.

For high interfacial potential Overbeek[14] gives

$$-T\Delta S_{scl} \ = G_{\mathrm{scl}} \frac{\psi_0 - 3 + 6\,\mathrm{e}^{-\frac{\psi_0}{2}}}{\psi_0 - 2 + 4\,\mathrm{e}^{-\frac{\psi_0}{2}}} > \Delta H_{\mathrm{scl}}$$

with

$$\Delta H_{\mathrm{scl}} = G_{\mathrm{scl}} \frac{1 - 2\,\mathrm{e}^{-\frac{\psi_0}{2}}}{\psi_0 - 2 + 4\,\mathrm{e}^{-\frac{\psi_0}{2}}}$$

where $\psi_0 = e\varphi_0/kT$ and φ_0 = interfacial electric potential,

while at small ψ_0, $-T\Delta S_{scl} \simeq \Delta H_{\mathrm{scl}}$.

The equations hold for a flat layer situation.

For not too small φ_0, the approach of two equally charged interfaces is primarily hindered by the loss of entropy on confining the distribution. The total G-curve of approach increases (maximum at ~ $\lambda/4$ for the example treated in Overbeek)[14] and then decreases towards the value of the aggregated layers. This maximum refers to a transition state and holds irrespective of the aggregation free enthalpy being negative or positive.

Obviously λ - in interplay with the Na-activity a_{Na} (OCV) - determines the staging situation. If a_{Na} is very small, the Na-layers will be randomly separated by distances larger than a few times λ. To be precise, variations of the surface potential and additional effects due to strain and electron correlation have to be considered.

4 Standard free enthalpy of aggregation

So far we have not explicitly addressed the full standard entropy ΔS° of aggregation, i.e., the entropy connected with assembling isolated chemical bilayers to higher aggregates. There are essentially three contributions: the first is the change of the phononic entropy, the second the entropy loss as a consequence of the loss of space charge layers, the third the entropy change on the configurational change plus the chemical interaction on aggregation. As shown above, the second term is large and assumed to be dominant. Contrarily, the corresponding enthalpy change is most probably dominated by the chemical term, as the space charge enthalpy change should be small. When comparing the insertion enthalpies of Na at high and low T, we need to consider the large enthalpy change of Na metal from LT to HT, i.e., the specific heat which even includes the melting enthalpy, as well as the aggregation enthalpy

when going from the HT (chemical bilayers) to LT (aggregated layers), what is more, the thermal enthalpy change owing to the specific heat of the Na-containing HOPG.

Let us consider the different contributions:
The thermal enthalpy change of Na (ΔH_{th}(Na)) between 700 K and 300 K is 14.7 kJ/mol.[6]
The thermal enthalpy change of the Na containing HOPG can, as the Na content (x) is small, be approximated by $1 + x$ times the value for pure graphite, where the thermal enthalpy change of pure graphite (ΔH_{th}(C)) between 700 K and 300 K is 5.7 kJ/mol.[6]

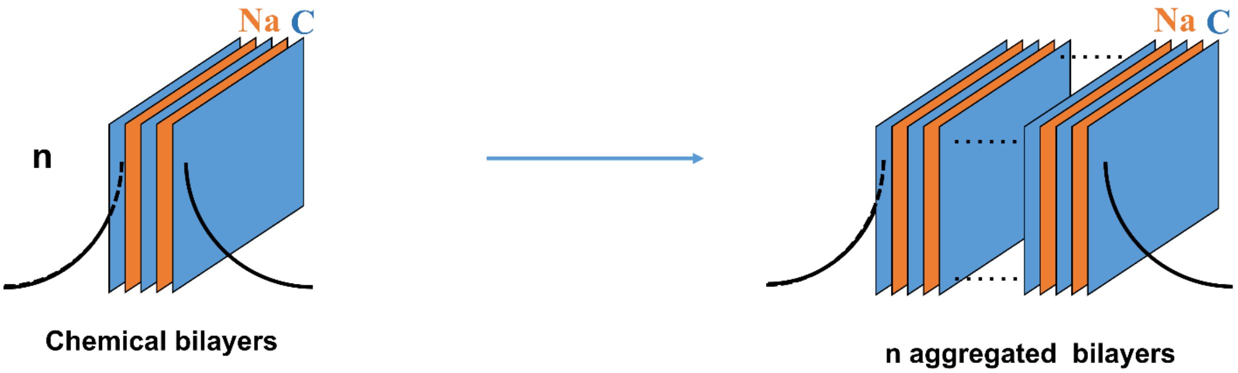

Fig. SⅤ-4 Sketch for chemical bilayer aggregation to higher aggregates, resulting in loss of space charge zones (also shown in the main text).

If changes in the phononic contributions are negligible, the (standard) aggregation entropy can be estimated from the loss of the space charge zones. As shown in Fig. SⅤ-4, for the aggregation of n isolated bilayers to a 2n-layer aggregate the loss of space charge zones is (2n-2) for n chemical bilayers. For large n-values we count 2n per n bilayers or, if we assume a 50 % occupation, per n fully occupied monolayers. This limit of 2 per full layer is taken in the further evaluation.

Let us take the text example revealing that at 700 K almost exclusively chemical bilayers are observed while at 300 K mostly aggregates (triple layers, quadruple and higher aggregates are observed). Then

$$\Delta H^{\circ}/700\ \mathrm{K}\ \ > \Delta S^{\circ} > \Delta H^{\circ}/300\ \mathrm{K}$$

where ΔH° is standard aggregation enthalpy. When we again refer to Overbeek's work.[14] We may find expression for the free energy (free enthalpy) for losing two space charge zones, namely

$$G = \varepsilon_0 \varepsilon_r \kappa (\frac{kT}{e})^2 \left\{ 8 \cosh \frac{\psi_0}{2} - 8 \right\}$$

where $\psi_0 = e\varphi_0/kT$ and $\kappa^{-1}(= \lambda)$ stands for the Debye length, ε_0 and ε_r the vacuum and relative dielectric constant, respectively. The interfacial electric potential φ_0 can be obtained via

$$\varphi_0 = \frac{2kT}{e}\sinh^{-1}\frac{eq}{2\varepsilon_0\varepsilon_r kT\kappa}$$

where q is the surface charge density (charge per area).

When solely relying on space charge effects, we find the bilayer aggregation free enthalpy to be 0.065 J/m^2 at 500 K and 0.038 J/m^2 at 300 K (half occupied relative to a NaC_8 structure, the relative dielectric constant is assumed to be 10, and the screening length (Debye length) is taken as 1 nm[15]. (The free enthalpies are 0.087 J/m^2 at 500 K and 0.052 J/m^2 at 300 K when considering a half occupied NaC_6 structure). However, we need to take also the middle C-layer into account. It is a reasonable approach to tentatively assume that the middle layer takes up half of the total charge of the bilayer, so that each double layer contains 1/4 of the total charge, so a value that is smaller by a factor of 2 compared to the previous value. This factor becomes the smaller, the larger n, so it is even more reasonable to neglect the space charge layer contribution in the n-aggregate (Here we ignore solubility changes with temperature. Higher solubilities would weaken the last statement). (In this situation, we obtain the bilayer aggregation free enthalpy due to space charges to be 0.032 J/m^2 at 500 K and 0.019 J/m^2 (10 kJ/mol) at 300 K for NaC_8 structure; the aggregation free enthalpy values are 0.043 J/m^2 at 500 K and 0.026 J/m^2 at 300 K in terms of NaC_6 structure).

From these values, one can extract an entropy of bilayer aggregation due to space charges of -1.35×10^{-4} J/m^2K, and an enthalpy term of -0.0025 J/m^2. (The entropy and the enthalpy are -1.75×10^{-4} J/m^2K and -0.0005 J/m^2 for the case of NaC_6 structure). Then the bilayer aggregation free enthalpy due to space charges at 700 K is found to be 0.092 J/m^2 (23 kJ/mol). (The value is 0.122 J/m^2 (23 kJ/mol) in terms of NaC_6 structure). When we take account of the situation in which each double layer contains 1/4 of the total charge, we find the entropy of bilayer aggregation to be -6.5×10^{-5} J/m^2K, and the enthalpy term to be -0.0005 J/m^2 for NaC_8 structure. (The entropy and enthalpy values are -8.5×10^{-5} J/m^2K and 0.0005 J/m^2 for NaC_6 structure). Then the bilayer aggregation free enthalpy due to space charges at 700 K is obtained to be 0.045 J/m^2 (23 kJ/mol). (The value is 0.06 J/m^2 (23 kJ/mol) in terms of NaC_6 structure). Even though the values in "J/m^2" are different for different situations (different charge densities), when we consider the charge density variation and convert the energy per area into the energy per mole ("J/m^2" into "kJ/mol"), all the situations give a similar value of aggregation free enthalpy.

Now we refer to the total aggregation processes (beyond space charges): As the related space charge enthalpy (see above) is negligible we have essentially the chemical term (ΔH_{ch}) dominating ΔH° (total aggregation process). (Such chemical term will include bonding effects and Madelung terms.) From this we can calculate free enthalpies of total aggregation for 700 K and 300 K (including ΔH°). As the first value must be positive and the second one negative, ΔH_{ch} can be assessed to be in the range of -10 kJ/mol and -23 kJ/mol, probably close to the latter as we still observe a small portion of triple layer at 700 K. The heterogeneity at 300 K is to be ascribed to not having reached equilibrium.

Note that this aggregation energy is an average value for aggregating bilayers to the aggregates at room temperature (without thermal contribution). It is intelligible that the aggregation energy per particle of bilayers to quadruple layers is larger than for the higher aggregation. This is even more pronounced for the aggregation of hypothetical monolayers to bilayers what explains the fact that monolayers have not been observed for Na. (The improved Madelung energy and/or the increased electron delocalization may account for this.) In the case of Li the ionicity as major reason for the energy gain is much lower while for K etc. the strain energy seems to dominate. It is pertinent to recall that the strain energy is proportional to the square of the misfit meaning that doubling the misfit will lead to an energy twice the sum of the strain energy of two individual misfitting layers. When treating the virtual transformation of filled monolayers to half-filled bilayers by the above arguments one has also to take account of the effects of lower occupancy on misfit and on space charge potential.

For the other alkali metals, also a tendency for dissociation at higher temperature[16] is observed but less than for Na conforming to the more favorable situation of full aggregates for the other alkali elements (experiments and DFT).[11, 17]

5 Enthalpies and entropies of intercalation

So far, we have been concerned with thermodynamic problems in which the overall configurational entropy did not play a significant role (the configuration problem in Section 4) still referred of individual space charge layers).

Here we consider very low Na concentrations for which we can assume random distribution, i.e., before a critical concentration is reached opening pathways of “pipe” diffusion.

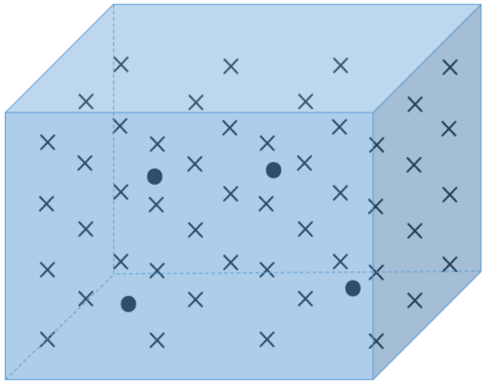

Fig. S V -5 Statistics of random Na distribution in three dimensions.

Then (see Figure S V -5) the same treatment as in 1) but now in a three dimension-space gives us appreciable numbers for the configurational entropy. The resulting mass action law for the process

$$x \text{ Na} + \text{C} \rightleftharpoons \text{Na}_x\text{C}$$

yields

$$[\text{Na}]_{\text{eq}} = \mathring{a}_{\text{Na}} \exp\left(-\frac{\Delta H^{\circ}_{\text{ins}}}{RT}\right) \exp\left(\frac{\Delta S^{\circ}_{\text{ins}}}{R}\right)$$

($\mathring{a}_{\text{Na}}$= activity of Na in Na, can be taken as 1).

The equilibrium Na concentration logarithmically plotted vs. *1/T* (Van't Hoff plot) delivers $\Delta H^{\circ}_{\text{ins}}$ and $\Delta S^{\circ}_{\text{ins}}$ (see the main text). The slope is well defined at high temperature (HT), whereas a lower slope (or even sign inversion) is indicated by the measurement at low temperature (LT). This LT value can be assessed within our model by the thermodynamic cycle to be described now. Note that we certainly overstress the random assumption. The statistical problem involving aggregation is more complicated and in view of the lack of information not tractable yet. At any rate, the activity coefficient is smaller than 1 and the concentration curve is higher than the linear $\ln a$ vs. $1/T$ plot expected from a random situation. So $\Delta H^{\circ}_{\text{ins}}$ and $\Delta S^{\circ}_{\text{ins}}$ have to be considered as effective values. Note also that the ab-initio calculations usually refer to $\Delta H^{\circ}_{\text{ins}}$ and do not only ignore $\Delta S^{\circ}_{\text{ins}}$, but also $\Delta S_{\text{conf.}}$.

If we assume the Na concentrations at HT and LT to be roughly the same (e.g., $y{\sim}0.01$), we can write the chemical processes as follows (see also the thermodynamic cycle as shown in Fig. 6 in the main text):

$$y \text{ Na (300 K)} + \text{C (300 K)} \rightarrow \text{Na}_y\text{C (300 K)}$$

$$\text{Na}_y\text{C (300 K)} \rightarrow \text{Na}_y\text{C (700 K)}$$

$$\mathrm{Na}_y\mathrm{C}\ (700\ \mathrm{K}) \rightarrow y\ \mathrm{Na}\ (700\ \mathrm{K}) + \mathrm{C}\ (700\ \mathrm{K})$$

The overall reaction can be written as

$$y\ \mathrm{Na}\ (300\ \mathrm{K}) + \mathrm{C}\ (300\ \mathrm{K}) \rightarrow y\ \mathrm{Na}\ (700\ \mathrm{K}) + \mathrm{C}\ (700\ \mathrm{K})$$

From this set of equations, we obtain the enthalpy relation as

$y\ \Delta H_{\mathrm{th}}(\mathrm{Na}) + \Delta H_{\mathrm{th}}(\mathrm{C})$

$= y\ \Delta H_{\mathrm{ins}}(300\ \mathrm{K}) + \Delta H_{\mathrm{th}}(\mathrm{Na}_{0.01}\mathrm{C}) + y\ \Delta H_{\mathrm{dis}} - y\ \Delta H_{\mathrm{ins}}(700\ \mathrm{K})$

where ΔH_{dis} is disaggregation enthalpy, the specific heat ΔH_{th}(Na) also includes melting enthalpy of Na, and the thermal enthalpy change of the Na containing carbon is approximated by

$\Delta H_{\mathrm{th}}(\mathrm{Na}_y\mathrm{C}) = (1+y)\Delta H_{\mathrm{th}}(\mathrm{C})$ (see 4)).

Then we have the insertion enthalpy difference

$$\Delta H_{\mathrm{ins}}(300\ \mathrm{K}) - \Delta H_{\mathrm{ins}}(700\ \mathrm{K}) = \Delta H_{\mathrm{th}}(\mathrm{Na}) - \Delta H_{\mathrm{th}}(\mathrm{C}) - \Delta H_{\mathrm{dis}}$$

Combining with the values given in 4) (note that here we use the upper limit of $\Delta H_{\mathrm{dis}}(\equiv -\Delta H_{\mathrm{ch}}) = (20 \pm 4)\ \mathrm{kJ/mol}$, we can calculate the difference between the insertion enthalpies at 300 K and 700 K to be $(-11 \pm 4)\ \mathrm{kJ/mol}$. Taking the latter from the HT slope of Fig. 4(a) in the main text, which is $8.5\ \mathrm{kJ/mol}$, we estimate the LT value as ($-2.5\ \pm\ 4$) kJ/mol, which can explain the trend of our experimental results. We obtain from Fig. 4(a) (main text) the insertion entropy as $\Delta S^{\circ}_{\mathrm{ins}}$ at HT to be $-26.6\ \mathrm{J/mol\ K}$. We may assume that this value is essentially due to the loss of Na(s) where entropy is around $50\ \mathrm{J/mol\ K}$. In fact, the values for other alkali metals are similar. Note that the values in the literature (e.g., Ref.[16c] even though labeled as ΔS°, contain still a configurational contribution). However, the extracted positive insertion enthalpy causes the significant discrepancy in comparison to other alkali metals, where all the others have negative insertion enthalpies (e.g., $-10\ \mathrm{kJ/mol}$ for Li[18]and $-35\ \mathrm{kJ/mol}$ for K[16c]).

Alternatively to this mechanistic picture, we can also understand the upwards bending in Fig. 4(a) (main text) at lower temperature by introducing overall activity coefficients which are smaller than unity (interaction) and are the smaller the higher the concentration. Notwithstanding the various rough approximations, the cycle shows the internal consistency of our model.

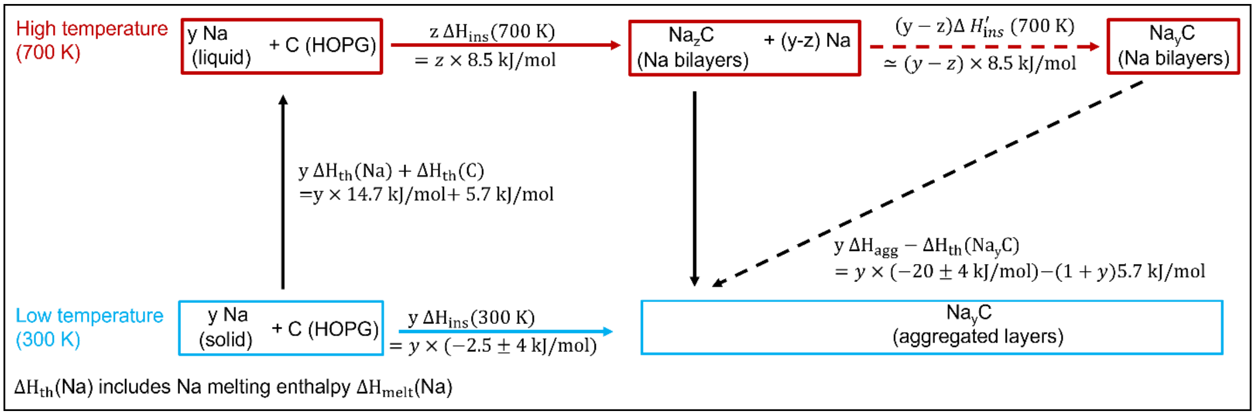

Fig. SⅤ-6. Unlike the approximate thermodynamic cycle shown as Fig. 6 in the main text, this figure is more general and includes the possibility that $\Delta H'_{\mathrm{ins}}$ differs from ΔH_{ins} (see below, Refinements of the above ΔH-cycle).

Refinements of the above ΔH-cycle

a) Taking into account that y (700 K) $\neq$ y (300 K). To simplify the notation, we use *y* for *y* (300 K) and *z* for *y* (700 K). (see Fig. SⅤ-6)

Then we have to write

(1) y Na (300 K) + C (300 K) → $\mathrm{Na}_y\mathrm{C}$ (300 K)

(2) $\mathrm{Na}_y\mathrm{C}$ (300 K) → $\mathrm{Na}_z\mathrm{C}$ (700 K) + $(y - z)$ Na (700 K)

(3) $\mathrm{Na}_z\mathrm{C}$ (700 K) → z Na (700 K) + C (700 K)

where the overall reaction (T) is

(T) y Na (300 K) + C (300 K) → z Na (700 K) + $(y - z)$ Na (700 K) + C (700 K)

Reaction (2) can be split into

(2a) $\mathrm{Na}_y\mathrm{C}$ (300 K) → $\mathrm{Na}_y\mathrm{C}$ (700 K)

(2b) $\mathrm{Na}_y\mathrm{C}$ (700 K) → $\mathrm{Na}_z\mathrm{C}$ (700 K) + $(y - z)$ Na (700 K)

ΔH(T) then follows as

$$\Delta H(\mathrm{T}) = \Delta H(1) + \Delta H(2\mathrm{a}) + \Delta H(2\mathrm{b}) + \Delta H(3)$$

or reformulated:

$y\,\Delta H_{\mathrm{th}}(\mathrm{Na}) + \Delta H_{\mathrm{th}}(\mathrm{C})$

$= y\,\Delta H_{\mathrm{ins}}(300\ \mathrm{K}) + \Delta H_{\mathrm{th}}(\mathrm{Na}_y\mathrm{C}) - (y - z)\,\Delta H'_{\mathrm{ins}}(700\ \mathrm{K}) + y\,\Delta H_{\mathrm{dis}} - z\,\Delta H_{\mathrm{ins}}(700\ \mathrm{K})$

where $\Delta H_{\mathrm{dis}} \equiv -\Delta H_{\mathrm{ch}}$ and $\Delta H'_{\mathrm{ins}}$ is the negative reaction enthalpy of reaction (2b) (i.e. Na insertion into Na-containing HOPG rather than in pure HOPG).

From this, it follows that

$y\, \Delta H_{\mathrm{ins}}(300\ \mathrm{K}) - z\, \Delta H_{\mathrm{ins}}(700\ \mathrm{K})$

$= y\, \Delta H_{\mathrm{th}}(\mathrm{Na}) + \Delta H_{\mathrm{th}}(\mathrm{C}) - \Delta H_{\mathrm{th}}(\mathrm{Na}_y\mathrm{C}) + (y - z)\, \Delta H'_{\mathrm{ins}}(700\ \mathrm{K}) + y\, \Delta H_{\mathrm{ch}}$

where $\Delta H_{\mathrm{th}}(\mathrm{Na}_y\mathrm{C}) = (1 + y)\, \Delta H_{\mathrm{th}}(\mathrm{C})$ (see 4)), yielding

$y\, \Delta H_{\mathrm{ins}}(300\ \mathrm{K}) - z\, \Delta H_{\mathrm{ins}}(700\ \mathrm{K}) + (z - y)\, \Delta H'_{\mathrm{ins}}(700\ \mathrm{K})$

$\cong y\, \Delta H_{\mathrm{th}}(\mathrm{Na}) + \Delta H_{\mathrm{th}}(\mathrm{C}) - (1 + y)\, \Delta H_{\mathrm{th}}(\mathrm{C}) + y\, \Delta H_{\mathrm{ch}}$

and finally

$$\Delta H_{\mathrm{ins}}(300\ \mathrm{K}) - \frac{z}{y}\, \Delta H_{\mathrm{ins}}(700\ \mathrm{K}) + \frac{(z-y)}{y}\, \Delta H'_{\mathrm{ins}}(700\ \mathrm{K}) \cong \Delta H_{\mathrm{th}}(\mathrm{Na}) - \Delta H_{\mathrm{th}}(\mathrm{C}) + \Delta H_{\mathrm{ch}}$$

If *y* = *z*, we trivially obtain the previous result

$$\Delta H_{\mathrm{ins}}(300\ \mathrm{K}) - \Delta H_{\mathrm{ins}}(700\ \mathrm{K}) \cong \Delta H_{\mathrm{th}}(\mathrm{Na}) - \Delta H_{\mathrm{th}}(\mathrm{C}) + \Delta H_{\mathrm{ch}}$$

If $y \neq z$, and considering dilute situation, the enthalpies of Na insertion into C and $\mathrm{Na}_x\mathrm{C}$ $(x \ll 1)$ are the same to a good approximation. Hence, we recover the previous result, viz

$$\Delta H_{\mathrm{ins}}(300\ \mathrm{K}) - \Delta H_{\mathrm{ins}}(700\ \mathrm{K}) \cong \Delta H_{\mathrm{th}}(\mathrm{Na}) - \Delta H_{\mathrm{th}}(\mathrm{C}) + \Delta H_{\mathrm{ch}}$$

b) Taking Na^+ and e^- as independent charge carriers:

$y\, \mathrm{Na}\ (+\ \mathrm{C}) \rightarrow y\, \mathrm{Na}^+ + y\, \mathrm{e}^-$

or more precisely, $\mathrm{Na} + \mathrm{V_i} \rightarrow \mathrm{Na_i^{\cdot}} + \mathrm{e'}$

Assuming mass action laws, we could write

$$[\mathrm{Na_i^{\cdot}}][\mathrm{e'}] = a^{\circ} \exp\left(-\frac{\Delta H^{*}_{\mathrm{ins}}}{RT}\right) \qquad ([\mathrm{Na_i^{\cdot}}][\mathrm{e'}] = [\mathrm{Na_i^{\cdot}}]^2 = [\mathrm{Na}]^2)$$

Then we have

$$[\mathrm{Na}] = a^{\circ 1/2} \exp\left(-\frac{\Delta H^{*}_{\mathrm{ins}}}{2RT}\right)$$

As there is no separate impact on Na^{+} and e^{-} in our considerations, it is only a question of notation with $\Delta H_{\mathrm{ins}} = \Delta H^{*}_{\mathrm{ins}}/2$.

The above equation presupposes classic random distribution and independency, which are of course harsh approximations. Partial association can be introduced by corresponding equilibria according to defect chemistry.

6 Special energetic role of bilayers

In order to understand the special energetics of bilayer formation from monolayers of ionized alkali metals, we need to understand the Madelung effects as well as the delocalization effects. For simplicity we assume for the Madelung problem rock-salt structured layers and for the delocalization homogeneous nano-boxes. Note that the estimation of lattice energies of metals by considering a lattice of metal cations and electrons as anions has been used by Haber and shown to be semi-quantitavely applicable investigated by Meyer.[19]

Firstly we calculate the Madelung factors (M) of mono-, bi- and multilayers (rocksalt structure).
From the literature,[20] we know

M_{3D} = 1.748; $M_{2D\ (monolayer)}$ = 1.616; $M_{1D\ (monolayer)}$ = 2ln2 = 1.386

We consider the transition

(a) from 1D-Monolayer (1.386)

to 1D-Bilayer etc.

up to 1D-Multilayer = 2D-Monolayer (1.616)

(b) from 2D-Monolayer (1.616)

to 2D-Bilayer etc.

up to 2D-Multilayer = 3D-Crystal (1.748)

Let M_0 be the sum within a layer, M_1 the sum of a given to the next layer, M_2 the sum to the second next layer, and M_3 the one to the third next layer, then we can state:

Monolayer: $M_{\text{monolayer}} = M_0$

Bilayer: $M_{\text{bilayer}} = 2(M_0 + M_1)/2 = M_0 + M_1$

Trilayer: $M_{\text{trilayer}} = 2(M_0 + M_1 + M_2)/3 + (M_0 + 2M_1)/3 = M_0 + 4M_1/3 + 2M_2/3$

Quadruplelayer: $M_{\text{quadruplelayer}} = 2(M_0 + M_1 + M_2 + M_3)/4 + 2(M_0 + 2M_1 + M_2)/4 = M_0 + 3M_1/2 + M_2 + M_3/2$

(a) 1D monolayer (1D chain, alternating +/- charges along the x-axis)

$$M_{\text{intra}} = M_0 = 2\sum_{n=1}^{\infty}\frac{(-1)^n}{n} = 2\ln 2 \approx 1.386$$

1D bilayer (add an opposite chain at a distance of *d*)

$$M_{\text{inter}} = M_1 = \sum_{n=-\infty}^{\infty}\frac{(-1)^{n+1}}{\sqrt{n^2+d^2}}$$

Then the total Madelung constant is

$$M_{\text{1D-bilayer}} = 2\sum_{n=1}^{\infty}\frac{(-1)^n}{n} + \sum_{n=-\infty}^{\infty}\frac{(-1)^{n+1}}{\sqrt{n^2+d^2}}$$

For 1D trilayer and quadruplelayer, we have

$$M_{\text{1D-trilayer}} = 2\sum_{n=1}^{\infty}\frac{(-1)^n}{n} + \frac{4}{3}\sum_{n=-\infty}^{\infty}\frac{(-1)^{n+1}}{\sqrt{n^2+d^2}} + \frac{2}{3}\sum_{n=-\infty}^{\infty}\frac{(-1)^n}{\sqrt{n^2+4d^2}}$$

$$M_{\text{1D-quadruplelayer}} = 2\sum_{n=1}^{\infty}\frac{(-1)^n}{n} + \frac{3}{2}\sum_{n=-\infty}^{\infty}\frac{(-1)^{n+1}}{\sqrt{n^2+d^2}} + \sum_{n=-\infty}^{\infty}\frac{(-1)^n}{\sqrt{n^2+4d^2}} + \frac{1}{2}\sum_{n=-\infty}^{\infty}\frac{(-1)^{n+1}}{\sqrt{n^2+9d^2}}$$

If d=1 (ions are equally spaced), numerical evaluations give:

$$M_{\text{1D-bilayer}} \approx 1.504$$

$$M_{\text{1D-trilayer}} \approx 1.541$$

$$M_{\text{1D-quadruplelayer}} \approx 1.560$$

The Madelung factor jump from 1D monolayer to 1D bilayer is greater than that from bilayer to trilayer (and further to multilayers). It will converge slowly toward the 2D monolayer value (1.616).

(b) 2D monolayer

$$M_{\text{intra}} = \sum_{(i,j)\neq(0,0)} \frac{(-1)^{i+j}}{\sqrt{i^2+j^2}} \approx 1.616$$

2D bilayer (add a second layer at a distance of d)

$$M_{\text{inter}} = \sum_{(i,j)} \frac{(-1)^{i+j+1}}{\sqrt{i^2+j^2+d^2}}$$

Then the total Madelung constant is

$$M_{\text{2D-bilayer}} = \sum_{(i,j)\neq(0,0)} \frac{(-1)^{i+j}}{\sqrt{i^2+j^2}} + \sum_{(i,j)} \frac{(-1)^{i+j+1}}{\sqrt{i^2+j^2+d^2}}$$

For 2D trilayer and quadruplelayer, the Madelung factor can be written as

$$\mathrm{M}_{\text{2D-trilayer}} = \sum_{(i,j)\neq(0,0)} \frac{(-1)^{i+j}}{\sqrt{i^2+j^2}} + \frac{4}{3}\sum_{(i,j)} \frac{(-1)^{i+j+1}}{\sqrt{i^2+j^2+d^2}} + \frac{2}{3}\sum_{(i,j)} \frac{(-1)^{i+j}}{\sqrt{i^2+j^2+4d^2}}$$

$$\begin{aligned}\mathrm{M}_{\text{2D-quadruplelayer}} &= \sum_{(i,j)\neq(0,0)} \frac{(-1)^{i+j}}{\sqrt{i^2+j^2}} \\ &+ \frac{3}{2}\sum_{(i,j)} \frac{(-1)^{i+j+1}}{\sqrt{i^2+j^2+d^2}} + \sum_{(i,j)} \frac{(-1)^{i+j}}{\sqrt{i^2+j^2+4d^2}} + \frac{1}{2}\sum_{(i,j)} \frac{(-1)^{i+j+1}}{\sqrt{i^2+j^2+9d^2}}\end{aligned}$$

If d=1, numerical evaluations give:

$$M_{\text{2D-bilayer}} \approx 1.683$$

$$M_{\text{2D-trilayer}} \approx 1.705$$

$$M_{\text{2D-quadruplelayer}} \approx 1.716$$

The Madelung factor jump from 2D monolayer to 2D bilayer by a greater factor than that from bilayer to trilayer (and further to multilayers). It converges slowly toward the 3D bulk value (1.748).

In order to assess the delocalization effect, we calculate-notwithstanding the weakness (but finiteness) of the delocalization perpendicular to the layers when compared to the in-plane situation - the electron-in-the-box energies for box sizes (L, length) given by mono-, bi- and multilayers. Let us define N as charge number, then the free energy change corresponding to layer evolutions,

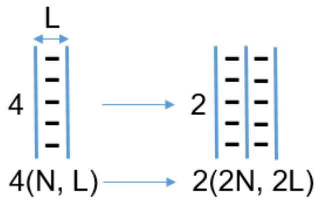

1a

$$\Delta E_{1a} = 2\frac{2N}{4L^2} - 4\frac{N}{L^2} = -3\frac{N}{L^2}$$

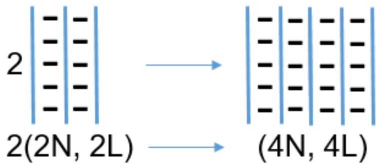

1b

$$\Delta E_{1b} = \frac{4N}{16L^2} - \frac{4N}{4L^2} = -\frac{3}{4}\frac{N}{L^2}$$

One finds

$$|\Delta E_{1b}| < |\Delta E_{1a}|$$

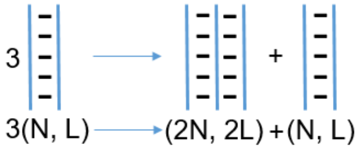

2a

$$\Delta E_{2a} = \frac{2N}{4L^2} + \frac{N}{L^2} - \frac{3N}{L^2} = -\frac{3}{2}\frac{N}{L^2}$$

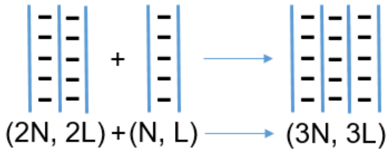

2b

$$\Delta E_{2b} = \frac{3N}{9L^2} - \frac{2N}{4L^2} - \frac{N}{L^2} = -\frac{7}{6}\frac{N}{L^2}$$

Then we have

$$|\Delta E_{2\mathrm{b}}| < |\Delta E_{2\mathrm{a}}|$$

Considering both the Madelung effects and the delocalization effects together, we can recognize that for Na the energy gain from monolayer to bilayer is greater than for further aggregation.

This is in striking difference to the other alkali metals. The reason, why bilayers are not favored for the higher alkali metals can be explained by size and thus by the larger strain, Why bilayers are also not found for Li, may be attributed to the higher covalency [EPR tests,[21] DFT calculation[5]]. The fact that conductivities are not very different for Li-inserted materials does note prove the opposite, as they are not DC values and thus do not represent carrier densities.)

7 Relation between intercalation free energy (ΔG_{ins}) and the free enthalpy measured by OCV (ΔG_{OCV})

$$\Delta G_{\mathrm{ins}} = \Delta G\{x\mathrm{Na} + \mathrm{C} \rightleftharpoons \mathrm{Na}_x\mathrm{C}\} = \mu_{\mathrm{Na}_x\mathrm{C}} - x\mu^0_{\mathrm{Na}} - \mu^0_{\mathrm{C}}$$

$$\Delta G_{\mathrm{OCV}} = \mu_{\mathrm{Na\ in\ Na}_x\mathrm{C}} - \mu^0_{\mathrm{Na}}$$

As $\mu_{\mathrm{Na}_x\mathrm{C}} = x\mu_{\mathrm{Na\ in\ Na}_x\mathrm{C}} + \mu_{\mathrm{C\ in\ Na}_x\mathrm{C}}$ it follows that

$$\Delta G_{\mathrm{ins}} = x\Delta G_{\mathrm{OCV}} + \mu_{\mathrm{C\ in\ Na}_x\mathrm{C}} - \mu^0_{\mathrm{C}}$$

where $\mu_{\mathrm{Na}_x\mathrm{C}}$ is chemical potential of Na containing-HOPG, μ^0_{Na} standard chemical potential of pure Na metal, μ^0_{C} standard chemical potential of pure HOPG, $\mu_{\mathrm{Na\ in\ Na}_x\mathrm{C}}$ chemical potential of Na in Na containing-HOPG, $\mu_{\mathrm{C\ in\ Na}_x\mathrm{C}}$ chemical potential of carbon in Na containing-HOPG.

(1) If Na is equilibrated with sodiated carbon (Na-containing HOPG) at the temperature of measurement:

$$\Delta G_{\mathrm{OCV}} = 0 \Leftrightarrow \Delta G_{\mathrm{ins}} = \mu_{\mathrm{C\ in\ Na}_x\mathrm{C}} - \mu^0_{\mathrm{C}}$$

(2) If carbon (HOPG) is equilibrated with sodiated carbon (Na-containing HOPG) at the temperature of measurement:

$$\Delta G_{\mathrm{ins}} = x\Delta G_{\mathrm{OCV}}$$

Supporting References

[1] C. Gan, C. Xiao, H. Wang, P. A. van Aken, R. Merkle, S. Bette, B. V. Lotsch, J. Maier, *arXiv preprint* **2025**, DOI: 10.48550/arXiv.2508.08806.

[2] Y. Z. Li, K. Yan, H. W. Lee, Z. D. Lu, N. Liu, Y. Cui, *Nat. Energy* **2016**, *1*.

[3] C. L. Xiao, H. G. Wang, R. Usiskin, P. A. van Aken, J. Maier, *Science* **2024**, *386*, 407–413.

[4] H. G. Wang, V. Srot, B. Fenk, G. Laskin, J. Mannhart, P. A. van Aken, *Micron* **2021**, *140*.

[5] Y. Liu, B. V. Merinov, W. A. Goddard III, *Proc. Natl. Acad. Sci.* **2016**, *113*, 3735–3739.

[6] I. Barin, G. Platzki, *Thermochemical Data of Pure Substances. Vol. 304* (VCh Weinheim) 3rd ed., VCH Verlagsgesellschaft mbH, Weinheim & VCH Publishers, Inc., New York, **1995**, pp. 209, 1102, 1130, 1692.

[7] a) J. Randles, Trans. Faraday Soc. **1948** 44, 327-338; b) A. Ševčík, *Collect. Czech. Chem. Commun.* **1948** 13, 349-377.

[8] K. Aoki, K. Tokuda, H. Matsuda, *J. Electroanal. Chem.* **1983** 146 (2), 417-424.

[9] B. E. Warren, *Phys. Rev.* **1941**, *59*, 693–698.

[10] T.R. Welberry, B.D. Butler, *J. Appl. Cryst.* **1994** 27, 205-231.

[11] M. S. Dresselhaus, G. Dresselhaus, *Adv. Phys.* **2006**, *30*, 139–326.

[12] L. Diehl, S. Bette, F. Pielnhofer, S. Betzler, I. Moudrakovski, G. A. Ozin, R. Dinnebier, B. V. Lotsch, *Chem. Mater.* **2018**, *30*, 8932–8938.

[13] J. Maier, *Physical chemistry of ionic materials : ions and electrons in solids*, 2nd edition. ed., John Wiley & Sons, Inc, Hoboken, **2023**, pp. 119-120.

[14] J. Theodoor, G. Overbeek, *Colloid. Surf.* **1990**, 51, 61-75.

[15] a) L. H. Thomas, *Mathematical Proceedings of the Cambridge Philosophical Society* **2008**, *23*, 542–548; b) E. Fermi, *Rend. Accad. Naz. Lincei* **1927**, 6 (602-607), 32; c) I.L. Spain, D.J. Nagel, *Mat. Sci. Eng.* **1977**, 31, 183-193.

[16] a) N. A. Cañas, P. Einsiedel, O. T. Freitag, C. Heim, M. Steinhauer, D.-W. Park, K. A. Friedrich, *Carbon* **2017**, *116*, 255–263; b) H. Oka, Y. Makimura, T. Uyama, T. Nonaka, Y. Kondo, C. Okuda, *J. Power Sources* **2021**, *482*; c) S. Aronson, F. J. Salzano, D. Bellafiore, *J. Chem. Phys.* **1968**, *49*, 434–439.

[17] a) K. Nobuhara, H. Nakayama, M. Nose, S. Nakanishi, H. Iba, *J. Power Sources* **2013**, *243*, 585–587; b) D. Igarashi, R. Tatara, R. Fujimoto, T. Hosaka, S. Komaba, *Chem. Sci.* **2023**, *14*, 11056–11066.

[18] Y. Reynier, R. Yazami, B. Fultz, *J. Power Sources* **2003**, *119-121*, 850–855.

[19] Meyer, K. *Physikalisch-chemische kristallographie*. (VEB Deutscher Verlag für Grundstoffindustrie Leipzig; **1968**, pp. 194-261.

[20] a) R. Hoppe, *Angew. Chem. Int. Ed. Eng.* **2003**, *5*, 95–106; b) D. Borwein, J. M. Borwein, K. F. Taylor, *J. Math. Phys.* **1985**, *26*, 2999–3009.

[21] P. Lauginie, H. Estrade, J. Conard. *Physica B+ C* **1980** 99 (1-4), 514-520.

Appendix

Consecutive STEM imaging

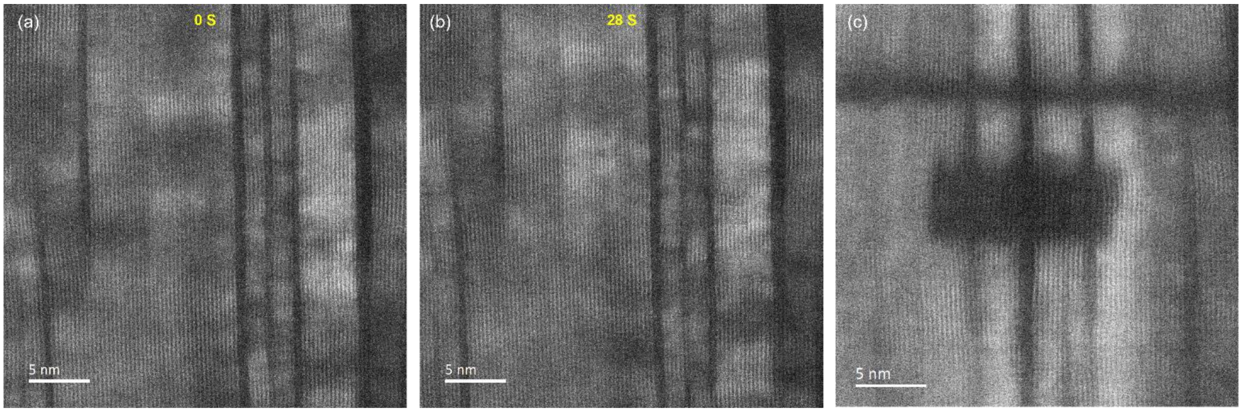

Fig. A1 STEM images of Na-intercalated graphite acquired with an 8C probe before (a) and after (b) eight scans (2 µs/pixel) over the same 1024 × 1024-pixel area. (c) STEM image showing electron-beam-induced damage obtained with a 5C probe; the dark lines and the boxed region indicate the damaged area. Sample: chemical intercalation of Na in HOPG at 400 °C for 2 weeks. The 8C probe was selected to do following tests.

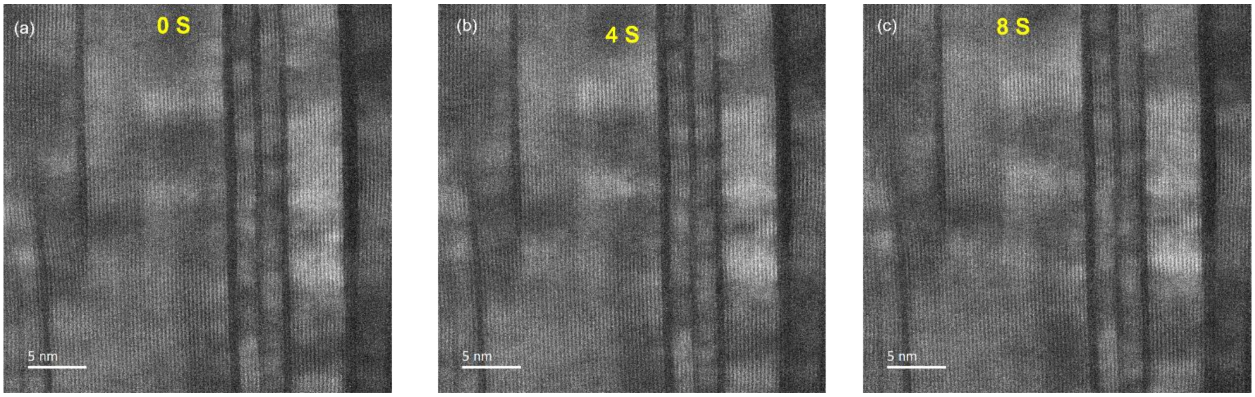

Fig. A2 Consecutive scans, 0 S, 4 S and 8 S (Probe size: 8C, Mag: 6M, Pixel time: 2 µs, Scan size: 1024*1024 pixels)

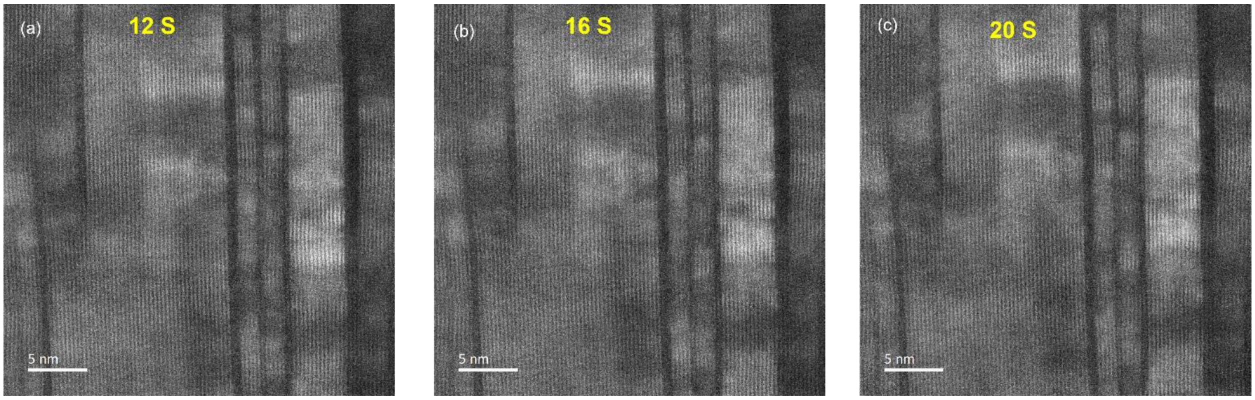

Fig. A3 Consecutive scans, 12 S, 16 S and 20 S (Probe size: 8C, Mag: 6M, Pixel time: 2 µs, Scan size: 1024*1024 pixels)

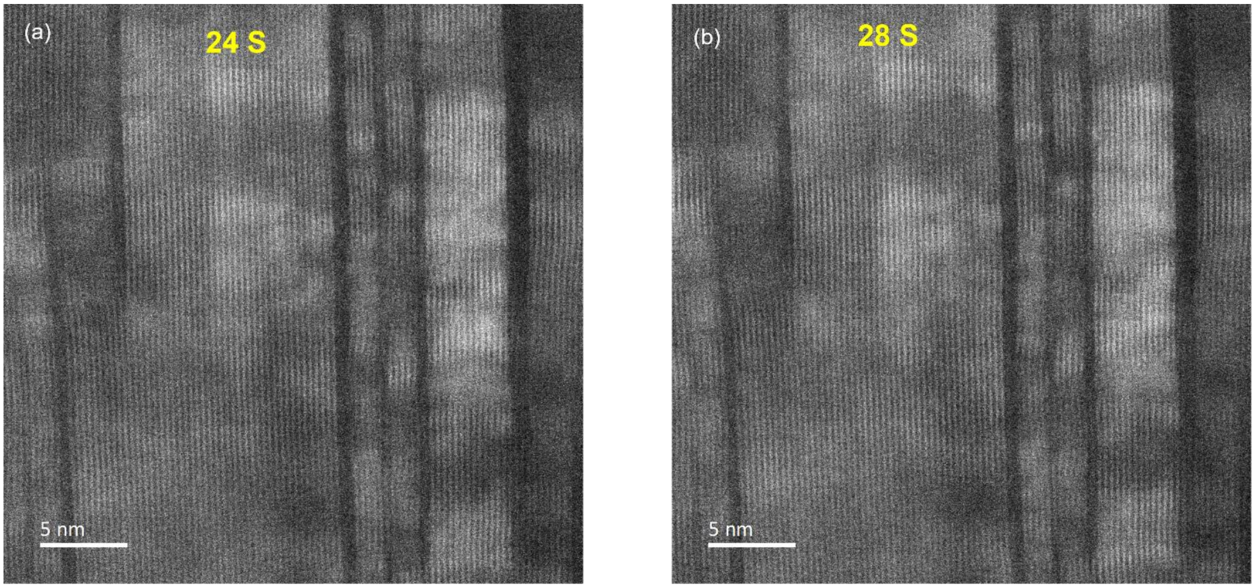

Fig. A 4 Consecutive scans, 24 S and 28 S (Probe size: 8C, Mag: 6M, Pixel time: 2 µs, Scan size: 1024*1024 pixels)

Note: It is worth noting that identification of Na via the Na-K edge in STEM-EELS is a well-established and widely accepted approach for probing Na distribution at the nanoscale and atomic scale, particularly in sodium battery systems (e.g., Nature Communications 12, 3066 (2021); Small Methods 1600063 (2017); Science Advances 3, e1700509 (2017)).
